# The Efficacy and Safety of Tirzepatide in Patients with Diabetes and/or Obesity: Systematic Review and Meta-Analysis of Randomized Clinical Trials

**DOI:** 10.3390/ph18050668

**Published:** 2025-04-30

**Authors:** Ligang Liu, Hekai Shi, Merilyn Xie, Yuxiao Sun, Milap C. Nahata

**Affiliations:** 1 Institute of Therapeutic Innovations and Outcomes (ITIO), College of Pharmacy, The Ohio State University, Columbus, OH 43210, USA; liu.10645@osu.edu; 2 Department of Bariatric and Metabolic Surgery, Fudan University Affiliated Huadong Hospital, Shanghai 200040, China; 22211280026@m.fudan.edu.cn; 3 Department of Pharmacy Practice, St. John’s University College of Pharmacy and Health Sciences, New York, NY 11439, USA; merilyn.xie18@my.stjohns.edu; 4 Department of Rehabilitation Medicine, West China Hospital, Sichuan University, Chengdu 610041, China; yuxiao.sun@wchscu.cn; 5 College of Medicine, The Ohio State University, 500 West 12th Ave., Columbus, OH 43210, USA

**Keywords:** tirzepatide, type 2 diabetes, obesity, GLP-1 receptor agonists, weight loss, glycemic control, meta-analysis, pharmacotherapy

## Abstract

**Background:** Obesity and type 2 diabetes (T2D) are major public health concerns. Tirzepatide has shown promise in recent clinical trials. This systematic review and meta-analysis aim to evaluate the efficacy and safety of tirzepatide in adults with obesity or type 2 diabetes, compared to placebo, GLP-1 receptor agonists (GLP-1 RAs), and insulin. **Method**: PubMed, Embase, and the Cochrane Library were searched on 17 January 2024, focusing on phase II and III randomized controlled trials (RCTs). We included studies involving adults with T2D or obesity, comparing tirzepatide to placebo, GLP-1 RAs, or insulin. The primary outcomes were the proportion of participants achieving ≥5%, ≥10%, and ≥15% weight loss targets. Secondary outcomes included changes in body weight, waist circumference, HbA1c levels, and blood pressure. Safety outcomes focused on adverse event rates. Meta-analyses were performed, and risk of bias was assessed using the Cochrane Risk-of-Bias tool version 2. **Results**: Fourteen RCTs involving 14,713 patients were included. Tirzepatide significantly increased the proportion of participants achieving weight loss targets, and reduced body weight, waist circumference, HbA1c, and blood pressure versus placebo and insulin. Compared with GLP-1 RAs, tirzepatide provided comparable or better outcomes in weight loss, waist circumference, and glycemic control. The incidence of gastrointestinal adverse events was significantly higher at all doses of tirzepatide compared to placebo and insulin. When compared with GLP-1 RAs, higher doses of tirzepatide were associated with increased risk of nausea, diarrhea, and decreased appetite, but not vomiting. **Conclusions:** Tirzepatide is an effective option for managing weight and improving metabolic outcomes in patients with T2D or obesity. However, it is associated with an increased risk of gastrointestinal adverse events, especially at higher doses. Therefore, close monitoring should be considered in clinical practice. **Registration**: PROSPERO CRD42021283449.

## 1. Introduction

Type 2 diabetes and obesity are closely linked to chronic conditions that impose a significant burden on global health systems [1]. Obesity is a risk factor for diabetes, heart failure, coronary heart disease, hypertension, and psychiatric comorbidities [2,3,4]. In the United States (US), the prevalence rates for obesity and diabetes were at 35.5% and 11.6% separately, and these numbers are on the rise [5,6,7]. Excess body fat, particularly abdominal fat, can lead to insulin resistance, progressing to diabetes eventually [8]. The economic implications of these conditions are profound, with the estimated costs reaching approximately USD 126 billion for obesity and USD 412.9 billion for diabetes in 2022 in the US [9,10].

To combat these issues, the evidence-based approach for the management of diabetes and obesity includes behavioral interventions, dietary modifications, increased physical activity, pharmacotherapy, and bariatric surgery [11,12]. Among the pharmacological options, glucagon-like peptide-1 receptor agonists (GLP-1 RAs) are increasingly used due to their multifaceted effects on glucose control and weight reduction [13]. These medications mimic the action of the glucagon-like peptide-1 hormone, which enhances insulin secretion, suppresses glucagon release, and slows gastric emptying, thereby promoting satiety and reducing appetite [14]. GLP-1 RAs, such as liraglutide, semaglutide, and dulaglutide, are important in the management of type 2 diabetes [15]. Semaglutide and liraglutide have also received approval for obesity management [16]. Moreover, GLP-1 RAs not only facilitate weight loss but also contribute to the reduction in cardiovascular disease risk factors, such as lowering blood pressure and improving lipid profile [17].

Tirzepatide, a novel medication for the treatment of type 2 diabetes, has gained attention due to its efficacy and safety [18]. It is a dual glucose-dependent insulinotropic polypeptide (GIP) and GLP-1 receptor agonist, enhancing its ability to manage diabetes. Recently, the US Food and Drug Administration (FDA) has approved tirzepatide for weight reduction in patients with obesity [19]. Some studies have demonstrated that tirzepatide achieves greater weight loss compared to existing GLP-1 RAs [20,21,22]. Tirzepatide has shown significant effects on weight management with the reductions in body weight, body mass index (BMI), and waist circumference (WC) in patients with obesity or diabetes [23]. However, previous meta-analyses included only a limited number of clinical trials. Recently, several large-scale randomized controlled trials (RCTs) specifically targeting patients with obesity but without diabetes have been published [24,25,26,27,28]. This expanded availability of clinical data allows for a more robust and comprehensive analysis of the impacts of tirzepatide beyond glycemic control.

This study aimed to evaluate the efficacy and safety of tirzepatide in patients with type 2 diabetes as well as in those with obesity who did not have diabetes. The primary objectives were to assess the efficacy and safety of tirzepatide. Secondary objectives were to compare the efficacy and safety of tirzepatide against placebo, insulin, and GLP-1 RAs on metabolic outcomes, weight reduction, and the frequency of adverse events and treatment discontinuation.

## 2. Results

**Figure 1:**
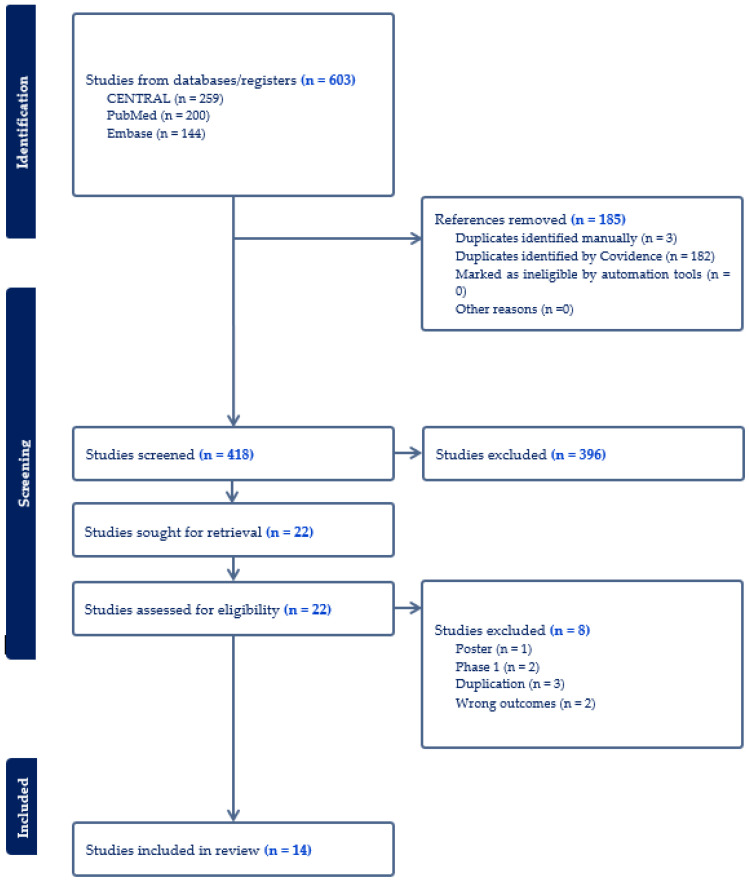
Flow diagram of the selection process of studies.

A total of 603 publications were identified in the initial search, among which 185 were excluded due to duplication. After an initial screening, 396 articles were further excluded, leading to a full-text review of 22 articles. Ultimately, 14 RCTs involving 14,713 patients were included in this meta-analysis (Figure 1) [24,25,26,27,28,29,30,31,32,33,34,35,36,37]. Of the RCTs included, twelve were multinational, while one was conducted solely in Japan and another in the US. Eleven trials included participants with diabetes, and three trials focused on participants with overweight or obesity without diabetes. Twelve trials examined the effect of 5 mg, 10 mg, or 15 mg tirzepatide, while two trials assessed the maximum tolerated dose of tirzepatide (10 mg or 15 mg). Of these trials, seven used placebos as control, four utilized insulin, and three used GLP-1 RAs as controls. A summary of the included studies and baseline characteristics of participants was in Table 1. Table 2 summarizes the change-from-baseline values for key efficacy and safety outcomes across included trials. These outcomes included reductions in A1c, proportions of participants achieving weight loss percentages, changes in waist circumference, blood pressure, and the frequency of common adverse events. Most of the included studies had a low risk of bias, and the detailed RoB-2 assessment was in Supplementary Table S1.

### 2.1. Proportion of Patients Achieving Body Weight Targets of 5%, 10%, 15%

All doses of tirzepatide demonstrated a higher proportion of patients achieving weight loss targets at least 5%, 10%, and 15% compared to placebo and insulin (Table 3 and Table 4; Supplementary Figures S1A,C–S3A,C). When compared with GLP-1RA, both tirzepatide 5 mg and 10 mg showed comparable or higher proportion of patients who achieved body weight reduction of 5%, 10%, with a significantly higher proportion of patients reaching the 15% weight loss target (Supplementary Figures S1B–S3B). Tirzepatide 15 mg consistently was associated with larger proportion of patients achieving reductions of 5% [OR 16.82 (95% CI 1.07–263.37), I^2^ = 98%], 10% [OR 12.20 (95% CI 2.60–57.27), I^2^ = 91%], and 15% [OR 20.25 (95% CI 3.64–112.56), I^2^ = 76%] (Table 4, Supplementary Figures S1B–S3B).

### 2.2. Change in Body Weight

Tirzepatide demonstrated greater body weight reduction across all doses when compared to placebo, starting with 5 mg [SMD −1.05 (95% CI −1.24, −0.87), I^2^ = 22%], followed by 10 mg [SMD −1.25 (95% CI −1.50, −1.00), I^2^ = 77%], and reaching the most substantial reduction with 15 mg [SMD −1.80 (95% CI −2.12, −1.49), I^2^ = 93%] (Supplementary Figure S4A). Compared to GLP-1RAs, tirzepatide also showed a more pronounced decrease in body weight (5 mg [SMD −0.55 (95% CI −1.07, −0.04), I^2^ = 93%], 10 mg [SMD −1.06 (95% CI −1.66, −0.46), I^2^ = 96%], and 15 mg [SMD −1.42 (95% CI −2.10, −0.74), I^2^ = 97%]). Additionally, tirzepatide achieved more significant reduction in body weight at doses of 5 mg [SMD −1.51 (95% CI −1.74, −1.29), I^2^ = 87%], 10 mg [SMD −1.96 (95% CI −2.24, −1.69), I^2^ = 89%], and 15 mg [SMD −2.23 (95% CI −2.58, −1.89), I^2^ = 97%] when compared with insulin (Table 4, Supplementary Figure S4C).

### 2.3. Change in Waist Circumference

Tirzepatide was associated with significantly greater reduction in WC compared to placebo. The reductions were observed at 5 mg [SMD −0.74 (95% CI −0.85, −0.63), I^2^ = 0%], 10 mg [SMD −0.91 (95% CI −1.08, −0.73), I^2^ = 63%], and 15 mg [SMD −1.41 (95% CI −1.81, −1.02), I^2^ = 96%]. When compared with GLP-1 RAs, tirzepatide also showed more pronounced reductions in WC for doses of 5 mg [SMD −0.71 (95% CI −1.27, −0.16), I^2^ = 84%], 10 mg [SMD −1.01 (95% CI −1.41, −0.61), I^2^ = 67%], and 15 mg [SMD −1.43 (95% CI −1.97, −0.88), I^2^ = 81%] (Table 4, Supplementary Figure S5).

### 2.4. Change in HbA1c

Compared to placebo, tirzepatide was associated with a significant reduction in HbA1c. The reductions observed were as follows: 5 mg [SMD −1.45 (95% CI −1.68, −1.21), I^2^ = 69%], 10 mg [SMD −1.59 (95% CI −1.82, −1.37), I^2^ = 62%], and 15 mg [SMD −1.56 (95% CI −1.85, −1.27), I^2^ = 91%] (Table 3, Supplementary Figure S6A). Compared with GLP-1 RAs, tirzepatide also demonstrated more significant reductions in HbA1c levels, starting from doses of 5 mg [SMD −0.60 (95% CI −1.13, −0.06), I^2^ = 93%], 10 mg [SMD −0.84 (95% CI −1.29, −0.39), I^2^ = 93%], and 15 mg [SMD −1.05 (95% CI −1.60, −0.50), I^2^ = 95%] (Table 4, Supplementary Figure S6B). Moreover, when compared with insulin, tirzepatide was more effective in reducing HbA1c across all doses: 5 mg [SMD −0.78 (95% CI −1.11, −0.44), I^2^ = 93%], 10 mg [SMD −0.98 (95% CI −1.33, −0.63), I^2^ = 94%], and 15 mg [SMD −1.09 (95% CI −1.44, −0.74), I^2^ = 94%] (Table 4, Supplementary Figure S6C).

### 2.5. Change in SBP

When compared with placebo, tirzepatide was associated with greater reduction in SBP, starting from the dose of 5 mg [SMD −0.38 (95% CI −0.53, −0.23), I^2^ = 37%], 10 mg [SMD −0.44 (95% CI −0.63, −0.25), I^2^ = 63%], and 15 mg [SMD −0.55 (95% CI −0.74, −0.36), I^2^ = 74%] (Supplementary Figure S6A). In comparison with GLP-1RA, tirzepatide did not exhibit a greater reduction in SBP across the doses 5 mg [SMD −0.28 (95% CI −0.60, 0.04), I^2^ = 55%], 10 mg [SMD −0.31 (95% CI −0.91, 0.29), I^2^ = 86%], and 15 mg [SMD −0.38 (95% CI −1.18, 0.41), I^2^ = 92%] (Supplementary Figure S6B). Tirzepatide showed superiority in reducing the SBP starting from the dose of 5 mg [SMD −0.43 (95% CI −0.59, −0.27), I^2^ = 76%], 10 mg [SMD −0.54 (95% CI −0.74, −0.34), I^2^ = 83%], and 15 mg [SMD −0.46 (95% CI −0.54, −0.38), I^2^ = 0%] versus basal insulin (Supplementary Figure S6C).

### 2.6. Change in DBP

Tirzepatide demonstrated a statistically significant reduction in SBP, starting from the dose of 5 mg [SMD −0.33 (95% CI −0.57, −0.09), I^2^ = 70%], 10 mg [SMD −0.29 (95% CI −0.51, −0.07), I^2^ = 77%], and 15 mg [SMD −0.42 (95% CI −0.54, −0.29), I^2^ = 55%] relative to placebo (Supplementary Figure S7A). When compared with GLP-1RA, tirzepatide did not achieve a greater reduction in SBP across the doses of 5 mg [SMD −0.18 (95% CI −0.65, 0.28), I^2^ = 78%], 10 mg [SMD −0.20 (95% CI −0.81, 0.42), I^2^ = 87%], and 15 mg [SMD −0.32 (95% CI −1.06, 0.43), I^2^ = 91%] (Supplementary Figure S7B). In contrast, tirzepatide showed superiority in lowering SBP across the dose of 5 mg [SMD −0.25 (95% CI −0.33, −0.17), I^2^ = 0%], 10 mg [SMD −0.30 (95% CI −0.42, −0.17), I^2^ = 59%], and 15 mg [SMD −0.19 (95% CI −0.32, −0.07), I^2^ = 56%] versus insulin (Supplementary Figure S7C).

### 2.7. Safety of Tirzepatide

#### 2.7.1. Any Adverse Events

Tirzepatide given at a dose of 5 mg [OR 1.54 (95% CI 1.24–1.90), I^2^ = 12%] and 15 mg [OR 1.53 (95% CI 1.06–2.218.52), I^2^ = 74%] were associated with higher incidence of any adverse events when compared with placebo (Table 5, Supplementary Figure S9A). However, 10 mg tirzepatide did not show any significant difference from the placebo [OR 1.36 (95% CI 0.98–1.89), I^2^ = 63%]. When compared with GLP-1 RAs, only tirzepatide 15 mg was associated with higher risk of any adverse events [OR 1.32 (95% CI 1.04–1.67), I^2^ = 0%], while tirzepatide 5 mg and 10 mg did not show significant differences (Table 6, Supplementary Figure S9B). All doses of tirzepatide showed a higher incidence of any adverse events when compared with insulin (Table 6, Supplementary Figure S9C).

#### 2.7.2. Serious Adverse Events

Tirzepatide, administered in doses of 5, 10, and 15 mg, did not show significant differences in the incidence of serious adverse events when compared with placebo and GLP-1 RAs. However, tirzepatide was associated with a significant reduction in serious adverse events relative to insulin (Table 6, Supplementary Figure S10).

#### 2.7.3. Treatment Discontinuation Due to Adverse Events

Tirzepatide, given at a dose of 5 mg [OR 1.76 (95% CI 1.07–2.91), I^2^ = 0%], 10 mg [OR 2.13 (95% CI 1.43–3.18), I^2^ = 17%] and 15 mg [OR 2.92 (95% CI 2.08–4.09), I^2^ = 23%], demonstrated a higher incidence of treatment discontinuation compared to placebo (Table 5, Supplementary Figure S11A). When compared with GLP-1RA, only 15 mg tirzepatide was associated with higher proportion of treatment discontinuation due to adverse events [OR 2.17 (95% CI 1.41–3.32), I^2^ = 0%], while 5 mg and 10 mg did not show any significant differences (Table 6, Supplementary Figure S11B). Relative to insulin, all doses of tirzepatide were associated with an increased rate of treatment discontinuation (Table 6, Supplementary Figure S11C).

#### 2.7.4. Gastrointestinal Adverse Events

The incidence of gastrointestinal adverse events, including nausea, vomiting, diarrhea, and decreased appetite, was significantly higher at all doses of tirzepatide compared to placebo (Table 5, Supplementary Figures S12A–S15A). When compared with GLP-1 RAs, only 15 mg tirzepatide was associated with significantly increased risk of nausea [OR 1.73 (95% CI 1.03–2.91), I^2^ = 57%]. There were no significant differences in vomiting rates across all tirzepatide doses compared to GLP-1 RAs. For diarrhea, both 10 mg and 15 mg tirzepatide were associated with higher risk [OR 1.48 (95% CI 1.07–2.04), I^2^ = 0%; OR 1.42 (95% CI 1.03–1.96), I^2^ = 0%]. Additionally, all doses of tirzepatide significantly increased the risk of decreased appetite (Table 6, Supplementary Figure S15B). Furthermore, all doses of tirzepatide demonstrated a higher risk of nausea, vomiting, diarrhea, and decreased appetite versus insulin (Table 6, Supplementary Figures S12–S15C).

## 3. Discussion

This systematic review and meta-analysis is the most current and comprehensive evaluation of phase 2 and phase 3 randomized clinical trials examining tirzepatide in patients with diabetes and/or obesity. It was observed that all doses of tirzepatide significantly increased the proportion of patients achieving weight loss targets of 5%, 10%, 15%, as well as reductions in waist circumference, HbA1c, and both systolic and diastolic blood pressure compared to placebo and insulin. Compared with GLP-1 RAs, all doses of tirzepatide led to significant reduction in body weight, waist circumference, and HbA1c. It also resulted in a similar or higher proportion of patients achieving 5%, 10%, and 15% weight loss. However, tirzepatide did not significantly decrease SBP and DBP compared to GLP-1 RAs. In terms of safety, tirzepatide was associated with more adverse events, serious adverse events, nausea, vomiting, diarrhea, and decreased appetite compared to both placebo and insulin. Higer doses of tirzepatide were associated with increased risk of overall adverse events, treatment discontinuation, nausea, diarrhea, and decreased appetite when compared to GLP-1 RAs.

Weight loss is paramount in the management of diabetes and obesity [38]. In individuals with type 2 diabetes, excess weight can compromise blood sugar regulation [39]. Conversely, weight reduction can markedly improve blood sugar control and decrease the risk of cardiovascular disease [40]. For those with obesity, weight loss offers numerous benefits, reducing the risk of developing diabetes and alleviating related complications like hypertension, sleep apnea, and joint pain [41]. Achieving weight loss targets of 5%, 10%, and 15% can significantly enhance health outcomes for individuals with obesity and type 2 diabetes. A weight reduction of between 5% and 10% can improve insulin sensitivity, lower blood pressure and triglycerides, and alleviate joint stress [42]. Reaching a 15% weight loss may further decrease the risk of heart attacks and strokes and enhance overall quality of life [42]. Previous studies have demonstrated that tirzepatide achieved a higher proportion of patients with body weight reduction goals of 5%, 10% or 15% [43,44]. Our findings showed that tirzepatide consistently led to a higher proportion of patients achieving substantial reductions in body weight, with marked success in reaching weight loss targets of 5%, 10%, and 15% across all doses compared to both placebo and basal insulin. Additionally, tirzepatide showed comparable or better outcomes relative to GLP-1RAs, particularly at the higher doses of 10 mg and 15 mg.

Tirzepatide demonstrated substantial reductions in waist circumference and HbA1c levels, surpassing the effects associated with alternative treatments, indicating that tirzepatide not only enhances glycemic control but also effectively reduces abdominal fat. The most significant decreases were observed at the highest dose of 15 mg. Previous studies have shown A1c reduction in tirzepatide was more significant than placebo, insulin, and GLP-1 RAs [43,45]. Moreover, tirzepatide was associated with notable improvements in blood pressure management [46]. While previous meta-analyses found significant decreases in blood pressure with tirzepatide compared to other therapies, they did not separate the comparisons against placebo, insulin, or GLP-1RAs [47]. Our results showed that tirzepatide provided greater reductions in both systolic and diastolic blood pressure compared to placebo and insulin. However, its blood pressure-lowering effects were similar to GLP-1RAs.

Tirzepatide has been recognized for its significant potential as a weight loss drug in patients with diabetes with or without obesity and shows increase in adverse events compared to other weight loss drugs. Tirzepatide at doses of 5 mg and 10 mg did not consistently demonstrate an increased risk of adverse events compared to placebo, insulin, and GLP-1RAs. However, 15 mg tirzepatide was associated with an increased risk of adverse events versus these treatments, suggesting a dose-dependent relationship in the occurrence of side effects. Importantly, there was no significant increase in the incidence of serious adverse events with tirzepatide compared to placebo, insulin, and GLP-1RAs. Nevertheless, higher doses of tirzepatide were associated with a greater likelihood of treatment discontinuation due to adverse events, especially at the 15 mg dose, both in comparison with placebo and GLP-1RAs. This pattern suggests potential tolerability issues for some patients at higher doses. Gastrointestinal adverse events such as nausea, vomiting, diarrhea, and decreased appetite were more prevalent in patients treated with tirzepatide compared to those receiving placebo and insulin. The highest dose of 15 mg showed an elevated risk of nausea and diarrhea when compared with GLP-1RAs. Although previous research has suggested that the overall safety profile of tirzepatide is comparable to GLP-1RAs [48,49], our findings indicated that higher doses of tirzepatide might lead to an increase in adverse events.

Overall, these results highlight the efficacy of tirzepatide in managing weight, improving glycemic control, and enhancing cardiovascular health for patients with diabetes or obesity. This positions tirzepatide as a compelling treatment option, particularly at higher doses which are associated with more significant benefits. However, the side effect profile at higher doses warrants careful consideration. Future research should continue to investigate the underlying mechanisms of these adverse effects and develop strategies to mitigate them, thus enhancing overall tolerability and improving patient quality of life. These advancements are crucial for the wider acceptance and utilization of tirzepatide in clinical practice, especially for the long-term management of type 2 diabetes and obesity.

This study represents the most current systematic review and meta-analysis on tirzepatide, encompassing fourteen phase II and III clinical trials. It provides a thorough evaluation of the efficacy and safety tirzepatide across various doses in treating obesity or type 2 diabetes. This study offered substantial insights into the dose-dependent effects of tirzepatide by conducting a subgroup analysis for different dose of tirzepatide. Moreover, by comparing tirzepatide with placebo, insulin, and GLP-1 RAs in separate analyses, this study offers a detailed evaluation of its relative efficacy and safety. However, it is not without limitations. First, the doses of GLP-1 RAs used in these clinical trials were generally lower than the approved therapeutic doses, which could affect the comparative effectiveness observed in the study. Future studies should include head-to-head trials comparing tirzepatide with appropriately dosed GLP-1 RAs. Additionally, the relatively short duration of the trials raises concerns about the long-term efficacy and safety of tirzepatide. Extended follow-up in future research is essential to ensure that tirzepatide remains safe for long-term use and does not exhibit delayed adverse effects. The study design of most clinical trials that compared insulin and GLP-1 RAs with tirzepatide were open-label trials, which might have introduced potential risks of performance and detection bias, particularly for subjective outcomes such as adverse events. Future trials with double-blind study design are needed to minimize this source of bias and strengthen the reliability of our findings. Lastly, all included studies were funded by Eli Lilly, the manufacturer of tirzepatide. It is unclear if that introduced any bias in reporting data.

## 4. Methods

The protocol for this systematic review and meta-analysis was registered with PROSPERO (registration no. CRD42021283449) and was reported in accordance with the Preferred Reporting Items for Systematic Reviews and Meta-Analyses (PRISMA) statement [50].

### 4.1. Search Strategy

A comprehensive search was conducted on 17 January 2024, using databases such as PubMed, Embase, and the Cochrane Library. The search terms included tirzepatide, LY3298176, “dual GIP and GLP-1RA”, “dual glucagon-like peptide and glucose-dependent insulinotropic polypeptide”, Zepbound, “Random”, “RCT”, “RCTs”, “randomized controlled trial”, “trial*”, and “clinical trial*”. Additionally, previously published meta-analyses and reviews were searched to identify relevant RCTs.

### 4.2. Eligibility Criteria and Exclusion Criteria

Participants who were adults with type 2 diabetes or those with obesity or overweight with one health-related condition (not diabetes) were included. The intervention was tirzepatide. The control included placebo, GLP-1 RAs, or insulin. Only phase II or phase III RCTs were eligible for inclusion. Studies excluded from this review were phase I clinical trials, pilot studies, observational studies, and abstracts at conferences.

### 4.3. Study Selection and Data Extraction

The results from the literature search were imported in Covidence for management. After the deduplication of records, the titles and abstracts underwent an initial screening for relevance, and potentially eligible records were then assessed in full text. Reasons for exclusion were carefully documented. The study selection process was independently carried out by two authors (L.L. and Y.S.), with any discrepancies resolved through discussion. Data extraction was carried out independently by the same authors (L.L. and Y.S.), including the author, publication year, trial name, study design, study population, sample size, gender distribution, mean age, intervention, comparator treatment, and study duration.

### 4.4. Outcomes

The primary efficacy outcomes were the proportion of participants achieving at least ≥5%, ≥10%, and ≥15% total weight loss. Secondary efficacy outcomes included changes in body weight, waist circumference, HbA1c, systolic blood pressure (SBP), and diastolic blood pressure (DBP). The safety outcomes included the incidence of any adverse events (AEs), serious adverse events, treatment discontinuation, and commonly reported gastrointestinal side effects such as nausea, vomiting, diarrhea, and decreased appetite.

### 4.5. Statistical Analysis

All analyses were conducted using R (version 4.0.3). Meta-analyses were performed when at least two studies provided relevant outcome data. For continuous outcomes, standard mean differences (SMD) and 95% confidence intervals (CIs) were calculated. For dichotomous outcomes, odds ratios (ORs) and 95% CIs were computed. Heterogeneity was assessed using I^2^, where an I^2^ value of less than 25% is low, 25% to 50% suggests low to moderate heterogeneity, and greater than 50% indicates moderate to high heterogeneity. Depending on the level of heterogeneity detected, a fixed-effects model was employed for analyses with low heterogeneity, while a random-effects model was used for high heterogeneity. Furthermore, subgroup analyses were carried out based on the type of comparator (placebo, GLP-1 RAs, or insulin) and the dose of tirzepatide (5 mg, 10 mg, or 15 mg). Funnel plots were generated to assess publication bias.

### 4.6. Quality and Risk-of-Bias Assessment

Two authors (L.L. and M.X.) independently assessed the risk of bias of included trials utilizing the Cochrane Risk-of-Bias tool for randomized trials (RoB-2). This evaluation tool has five domains, including randomization process, deviations from intended interventions, missing outcomes, measurement of the outcome, and selection of the reported result. Any disagreements were resolved by a third author (M.N.).

## 5. Conclusions

Tirzepatide has demonstrated efficacy in managing weight and improving metabolic outcomes in patients with type 2 diabetes or those with obesity. However, tirzepatide, particularly at higher doses, is associated with an increased risk of adverse events. These findings highlight the need for careful consideration of dose-related tolerability in clinical practice, ensuring that the benefits of tirzepatide outweigh the potential risks for each patient.

## Figures and Tables

**Table 1:** Baseline characteristics of the included studies.

Author (Year)	Trial Name	Design/Phase	Patient Population	Sample Size	BMI (kg/m^2^)	HbA1c (%)	Intervention	Comparator	Duration
Aronne, 2024 [24]	SURMOUNT-4	Phase 3, randomized, double-blind, placebo-controlled	≥18 years old, obesity or overweight without diabetes	670	38.4	5.5	The maximum tolerated dose of tirzepatide	Placebo	88-week
Wadden, 2023 [25]	SURMOUNT-3	Phase 3, randomized, parallel-arm, double-blind, placebo-controlled	≥18 years old, obesity or overweight without diabetes	579	35.9	5.4	The maximum tolerated dose of tirzepatide	Placebo	72-week
Garvey, 2023 [26]	SURMOUNT-2	Phase 3, double-blind, randomized, placebo-controlled	≥18 years old, with T2DM for at least 3 months, BMI ≥ 27 kg/m^2^	938	36·1	8·02	Tirzepatide (10 mg or 15 mg)	Placebo	72-week
Jastreboff, 2022 [29]	SURMOUNT-1	Phase 3, double-blind, randomized, placebo-controlled	≥18 years old, obesity or overweight without diabetes	2539	38.0	5.6	Tirzepatide (5 mg, 10 mg or 15 mg)	Placebo	72-week
Gao, 2023 [27]	SURPASS-AP-Combo	Phase 3, randomized controlled, open label	≥18 years old, with T2DM for at least 2 months, BMI ≥ 23 kg/m^2^	917	27.9	8.71	Tirzepatide (5 mg, 10 mg or 15 mg)	Insulin glargine	40-week
Inagaki, 2022 [30]	SURPASS J-mono	Phase 3, double-blinded, randomized, active-controlled	≥20 years old, with T2DM for at least 8 months, BMI ≥ 23 kg/m^2^	636	28·1	8·2	Tirzepatide (5 mg, 10 mg or 15 mg)	Dulaglutide 0.75 mg	52-week
Rosenstock, 2023 [28]	SURPASS-6	Phase 3b, randomized, open-label, parallel-group	≥18 years old, with T2DM inadequately controlled with basal insulin, BMI 23–45 kg/m^2^	1428	33	8.8	Tirzepatide (5 mg, 10 mg or 15 mg)	Insulin lispro	52-week
Dahl, 2022 [37]	SURPASS-5	Phase 3, randomized controlled, double-blind	With T2DM, BMI ≥ 23 kg/m^2^	475	33.4	8.3	Tirzepatide (5 mg, 10 mg or 15 mg)	Placebo	40-week
Del Prato, 2021 [32]	SURPASS-4	Phase 3, randomized, open-label, active-controlled, parallel-group	≥18 years old, with T2DM, BMI ≥ 25 kg/m^2^	1995	32·6	8·5	Tirzepatide (5 mg, 10 mg or 15 mg)	Insulin glargine 100 U/mL	52-week
Ludvik, 2021 [33]	SURPASS-3	Phase 3, randomized, active-controlled, open-label, parallel-group	≥18 years old, with T2DM for at least 3 months, BMI ≥ 25 kg/m^2^	1437	33.5	8.2	Tirzepatide (5 mg, 10 mg or 15 mg)	Insulin degludec U100/mL	52-week
Frías, 2021 [31]	SURPASS-2	Phase 3, open-label, randomized, active-controlled	≥18 years old, with T2DM, BMI ≥ 25 kg/m^2^	1878	34.2	8.3	Tirzepatide (5 mg, 10 mg or 15 mg)	Semaglutide 1 mg	40-week
Rosenstock, 2021 [36]	SURPASS-1	Phase 3, double-blind, randomized, placebo-controlled	≥18 years old, with T2DM, BMI ≥ 23 kg/m^2^	705	31.9	7.9	Tirzepatide (5 mg, 10 mg or 15 mg)	Placebo	40-week
Frias, 2020 [35]	NCT03311724	Phase 2, randomized, controlled, double-blind	With T2DM for at least 3 months, BMI 23–45 kg/m^2^	198	31.9	8.4	Tirzepatide (12 mg or 15 mg)	Placebo	12-week
Frias, 2018 [34]	NCT03131687	Phase 2, double-blind, randomized	18–75 years old, with T2DM for at least 3 months, BMI 23–50 kg/m^2^	318	32.4	8.1	Tirzepatide (1 mg, 5 mg, 10 mg or 15 mg)	Placebo or 1.5 mg dulaglutide	26-week

Abbreviations: T2DM: Type 2 Diabetes Mellitus; TZP: Tirzepatide; BMI: Body Mass Index. Note: All trials were funded by Eli Lilly and Company.

**Table 2:** Summary of Efficacy and Safety Outcomes Across Tirzepatide Clinical Trials.

	Trials													
Parameters	SURPASS-1	SURPASS-2	SURPASS-3	SURPASS-4	SURPASS-5	SURPASS-6	SURPASS-J-Mono	SURPASS-AP-Combo	NCT03131687	NCT03311724	SURMOUNT-1	SURMOUNT-2	SURMOUNT-3	SURMOUNT-4
Interventions	TZP 5 mg (121) vs. 10 mg (121) vs. 15 mg, (121) vs. placebo (115)	TZP 5 mg (470) vs. 10 mg (469) vs. 15 mg, (470) vs. semaglutide 1 mg (469)	TZP 5 mg (358) vs. 10 mg (360) vs. 15 mg, (359) vs. insulin degludec (360)	TZP 5 mg (329) vs. 10 mg (328) vs. 15 mg, (338) vs. insulin glargine (1000)	TZP 5 mg (116) vs. 10 mg (119) vs. 15 mg, (120) vs. placebo (120)	TZP 5 mg (243) vs. 10 mg (238) vs. 15 mg, (236) vs. insulin lispro (708)	TZP 5 mg (159) vs. 10 mg (158) vs. 15 mg, (160) vs. dulaglutide 0.75 mg (159)	TZP 5 mg (230) vs. 10 mg (228) vs. 15 mg, (229) vs. insulin glargine (230)	TZP 1 mg (52) vs. 5 mg (55) vs. 10 mg (51) vs. 15 mg, (53) vs. dulaglutide 1.5 mg (54) vs. placebo (51)	TZP 12 mg (29) vs. 15 mg, (28) vs. 15 mg (28) vs. placebo (26)	TZP 5 mg (630) vs. 10 mg (636) vs. 15 mg (630) vs. placebo (643)	TZP 10 mg (312) vs. 15 mg (311) vs. placebo (315)	MTD (287) vs. placebo (292)	MTD (335) vs. Placebo (335)
(number of participants)														
**Efficacy Outcomes**														
Change in A1c (%)	−1.87 vs. −1.89 vs. −2.07 vs. 0.04	−2.01 vs. −2.24 vs. −2.30 vs. −1.86	−1.93 vs. −2.20 vs. −2.37 vs. −1.34	−2.24 vs. −2.43 vs. −2.58 vs. −1.44	−2.11 vs. −2.40 vs. −2.34 vs. −0.86	−1.92 vs. −2.15 vs. −2.27 vs. −1.13	−2.4 vs. −2.6 vs. −2.8 vs. −1.3	−2.24 vs. −2.44 vs. −2.49 vs. −0.95	−0.7 vs. −1.6 vs. −2.0 vs. −2.4 vs. −1.1 vs. −0.1	−1.7 vs. −2.0 vs. −1.8 vs. 0.2	−0.40 vs. −0.49 vs. −0.51 vs. −0.07	−2.1 vs. −2.1 vs. −0.5	−0.5 vs. 0.0	−0.57 vs. −0.22
WL (kg)	−7.0 vs. −7.8 vs. −9.5 vs. −0.7	−7.8 vs. −10.3 vs. −12.4 vs. −6.2	−7.5 vs. −10.7 vs. −12.9 vs. 2.3	−7.1 vs. −9.5 vs. −11.7 vs. 1.9	−5.4 vs. −7.5 vs. −8.8 vs. 1.6	−6.7 vs. −9.2 vs. −11.0 vs. 3.2	−5.8 vs. −8.5 vs. −10.7 vs. −0.5	−5.0 vs. −7.0 vs. −7.2 vs. 1.5	−0.9 vs. −4.8 vs. −8.7 vs. −11.3 vs. −2.7 vs. −0.4	−5.3 vs. −5.5 vs. −5.7 vs. −0.5	n/a	n/a	n/a	n/a
≥5% WL, %	67 vs. 78 vs. 77 vs. 14	65 vs. 76 vs. 80 vs. 54	66 vs. 84 vs. 88 vs. 6	63 vs. 78 vs. 85 vs. 8	47.9 vs. 57.9 vs. 71.6 vs. 6.0	64 vs. 79 vs. 83 vs. 6	61 vs. 82 vs. 89 vs. 11	55.7 vs. 71.6 vs. 74.1 vs. 5.6	13.5 vs. 47.3 vs. 70.6 vs. 62.3 vs. 22.2 vs. 0	n/a	85.1 vs. 88.9 vs. 90.9 vs. 34.5	79.2 vs. 82.8 vs. 32.5	87.5 vs. 16.5	98.5 vs. 69.0
≥10% WL, %	31 vs. 40 vs. 47 vs. 1	34 vs. 47 vs. 57 vs. 24	37 vs. 56 vs. 69 vs. 3	36 vs. 53 vs. 66 vs. 2	20.7 vs. 41.6 vs. 40.7 vs. 0.8	33 vs. 52 vs. 61 vs. 2	34 vs. 50 vs. 67 vs. 3	26.8 vs. 41.9 vs. 45.1 vs. 0.5	5.8 vs. 16.4 vs. 39.2 vs. 37.7 vs. 9.3 vs. 0	n/a	73.4 vs. 85.9 vs. 90.1 vs. 13.5	63.4 vs. 69.6 vs. 8.7	88.0 vs. 4.8	94.0 vs. 44.4
≥15% WL, %	13 vs. 17 vs. 27 vs. 0	15 vs. 24 vs. 36 vs. 8	13 vs. 28 vs. 43 vs. 0	14 vs. 24 vs. 37 vs. <1	6.9 vs. 23.7 vs. 22.9 vs. 0	14 vs. 30 vs. 41 vs. 0	16 vs. 26 vs. 45 vs. <1	10.1 vs. 17.1 vs. 17.9 vs. 0	0 vs. 5.5 vs. 21.6 vs. 24.5 vs. 1.9 vs. 0	n/a	50.2 vs. 73.6 vs. 78.2 vs. 6.0	41.4 vs. 51.8 vs. 2.6	73.9 vs. 2.1	87.1 vs. 24.0
Change in WC, cm	−5.7 vs. −6.9 vs. −7.2 vs. −2.0	−6.9 vs. −9.6 vs. −9.9 vs. −5.6	n/a	n/a	−3.8 vs. −7.4 vs. −8.9 vs. +1.0	−5.7 vs. −7.8 vs. −9.6 vs. 2.1	n/a	n/a	−2.1 vs. −5.1 vs. −7.4 vs. −10.2 vs. −2.5 vs. −1.3	−4.8 vs. −4.9 vs. −4.9 vs. −2.5	−14.0 vs. −17.7 vs. −18.5 vs. −4.0	−10.8 vs. −13.1 vs. −3.3	−14.6 vs. 0.2	−22.5 vs. −9.3
Change in SBP, mmHg	−4.7 vs. −4.7 vs. −5.2 vs. −2.0	−4.8 vs. −5.3 vs. −6.5 vs. −3.6	−4.9 vs. −6.6 vs. −5.5 vs. 0.5	n/a	−6.1 vs. −8.3 vs. −12.6 vs. −1.7	−7.4 vs. −9.0 vs. −5.9 vs. −0.4	n/a	−6.7 vs. −7.2 vs. −7.3 vs. 1.1	0 vs. −2.6 vs. −1.3 vs. −1.0 vs. −1.5 vs. 1.7	n/a	−7.0 vs. −8.2 vs. −7.6 vs. −1.2	−6.3 vs. −1.2	−5.1 vs. 4.1	−9.3 vs. −2.4
Change in DBP, mmHg	−2.9 vs. −3.1 vs. −3.4 vs. −1.4	−1.9 vs. −2.5 vs. −2.9 vs. −1.0	−2.0 vs. −2.5 vs. −1.9 vs. 0.4	n/a	−2.0 vs. −3.3 vs. −4.5 vs. −2.1	−2.3 vs. −3.3 vs. −1.0 vs. −0.4	n/a	−4.0 vs. −3.6 vs. −3.4 vs. 0.9	−0.5 vs. −0.7 vs. −0.2 vs. −0.7 vs. −1.4 vs. 0.8	n/a	−5.2 vs. −5.5 vs. −4.6 vs. −1.0	n/a	−3.2 vs. 2.3	−5.5 vs. −1.7
Change in BMI (kg/m^2^)	−2.6 vs. −2.9 vs. −3.6 vs. −0.2	−2.9 vs. −3.8 vs. −4.6 vs. −2.3	n/a	n/a	−2.2 vs. −2.9 vs. −3.8 vs. +0.6	−2.6 vs. −3.8 vs. −4.5 vs. 1.4	n/a	n/a	−0.3 vs. −1.7 vs. −3.1 vs. −4.1 vs. −1.0 vs. −0.1	n/a	n/a	−4.7 vs. −5.4 vs. −1.2	−7.7 vs. 1.2	−10.0 vs. −3.6
**Safety Outcomes**														
Overall AE (%)	69 vs. 67 vs. 64 vs. 66	63.6 vs. 68.7 vs. 68.9 vs. 64.2	61 vs. 69 vs. 73 vs. 54	71 vs. 74 vs. 77 vs. 68	73.3 vs. 68.1 vs. 78.3 vs. 67.5	70.0 vs. 70.6 vs. 75 vs. 55.6	82 vs. 77 vs. 84 vs. 77	87 vs. 94.7 vs. 93 vs. 71.4	50 vs. 72.7 vs. 78.4 vs. 84.9 vs. 74.1 vs. 52.9	79.3 vs. 67.9 vs. 85.7 vs. 50.0	81.0 vs 81.8 vs 78.9 vs 72.0	78 vs 71 vs 76	87.1 vs 76.7	60.3 vs 55.8
SAE (%)	4 vs. 2 vs. 1 vs. 3	7.0 vs. 5.3 vs. 5.7 vs. 2.8	8 vs. 6 vs. 7 vs. 6	15 vs. 17 vs. 12 vs. 19	7.8 vs. 10.9 vs. 7.5 vs. 8.3	6.2 vs. 5.9 vs. 6.4 vs. 10.9	5 vs. 6 vs. 4 vs. 9	6.5 vs. 6.1 vs. 6.6 vs. 9.1	3.8 vs. 1.8 vs. 5.9 vs. 3.8 vs. 5.6 vs. 3.9	3.4 vs. 0 vs. 0 vs. 0	6.3 vs 6.9 vs 5.1 vs 6.8	6 vs 9 vs 7	5.9 vs 4.8	3 vs 3
AE leading to discontinuation (%)	3 vs. 5 vs. 7 vs. 3	6.0 vs. 8.5 vs. 8.5 vs. 4.1	7 vs. 10 vs. 11 vs. 1	11 vs. 9 vs. 11 vs. 5	6.0 vs. 8.4 vs. 10.8 vs. 2.5	4.1 vs. 4.6 vs. 9.3 vs. 2.4	8 vs. 10 vs. 10 vs. 6	4.3 vs. 13.2 vs. 12.2 vs. 2.7	3.8 vs. 9.1 vs. 5.9 vs. 24.5 vs. 11.1 vs. 3.9	3.4 vs. 3.6 vs. 0 vs. 3.8	4.3 vs 7.1 vs 6.2 vs 2.6	4 vs 7 vs 4	10.5 vs 2.1	1.8 vs 0.9
Nausea (%)	12 vs. 13 vs. 18 vs. 6	17.4 vs. 19.2 vs. 22.1 vs. 17.9	12 vs. 23 vs. 24 vs. 2	12 vs. 16 vs. 23 vs. 2	12.9 vs. 17.6 vs. 18.3 vs. 2.5	13.6 vs. 20.6 vs. 25.8 vs. 1.1	12 vs. 20 vs. 20 vs. 8	20.0 vs. 32.9 vs. 31.9 vs. 2.3	3.8 vs. 20.0 vs. 21.6 vs. 39.6 vs. 29.6 vs. 5.9	24.1 vs. 39.3 vs. 35.7 vs. 7.7	24.6 vs 33.3 vs 31.0 vs 9.5	20 vs 22 vs 6	39.7 vs 14.0	8.1 vs 2.7
Vomiting (%)	3 vs. 2 vs. 6 vs. 2	5.7 vs. 8.5 vs. 9.8 vs. 8.3	6 vs. 9 vs. 10 vs. 1	5 vs. 8 vs. 9 vs. 2	6.9 vs. 7.6 vs. 12.5 vs. 2.5	4.5 vs. 8.8 vs. 12.7 vs. 0.6	8 vs. 5 vs. 12 vs. 1	9.1 vs. 14.9 vs. 12.7 vs. 1.4	3.8 vs. 7.3 vs. 15.7 vs. 26.4 vs. 9.3 vs. 2.0	17.2 vs. 17.9 vs. 17.9 vs. 3.8	8.3 vs 10.7 vs 12.2 vs 1.7	11 vs 13 vs 3	18.1 vs 1.4	5.7 vs 1.2
Diarrhea (%)	12 vs. 14 vs. 12 vs. 8	13.2 vs. 16.4 vs. 13.8 vs. 11.5	15 vs. 17 vs. 16 vs. 4	13 vs. 20 vs. 22 vs. 4	12.1 vs. 12.6 vs. 20.8 vs. 10.0	11.9 vs. 15.1 vs. 11.0 vs. 2.4	17 vs. 9 vs. 11 vs. 7	33.5 vs. 45.2 vs. 44.1 vs. 1.4	13.5 vs. 23.6 vs. 23.5 vs. 32.1 vs. 16.7 vs. 3.9	31.0 vs. 35.7 vs. 32.1 vs. 7.7	18.7 vs 21.2 vs 23.0 vs 7.3	20 vs 22 vs 9	31.0 vs 9.2	10.7 vs 4.8
Constipation (%)	6 vs. 5 vs. 7 vs. 1	6.8 vs. 4.5 vs. 4.5 vs. 5.8	n/a	5 vs. 4 vs. 4 vs. <1	6.0 vs. 6.7 vs. 6.7 vs. 1.7	2.5 vs. 3.4 vs. 5.9 vs. 0.6	15 vs. 18 vs. 14 vs. 11	5.2 vs. 7.5 vs. 10.0 vs. 1.4	1.9 vs. 3.6 vs. 11.8 vs. 3.8 vs. 5.6 vs. 0	3.4 vs. 10.7 vs. 17.9 vs. 0	16.8 vs 17.1 vs 11.7 vs 5.8	8 vs 9 vs 4	23.0 vs 6.8	n/a

Abbreviations: MTD: maximum tolerated dose; WL: weight loss; BMI: body mass index; WC: waist circumference; A1c: hemoglobin A1c; SBP: systolic blood pressure; DBP: diastolic blood pressure; TZP: Tirzepatide; AE: adverse events; SAE: serious adverse events. n/a = not applicable.

**Table 3:** Meta-analysis results for tirzepatide versus placebo for efficacy outcomes.

Intervention	Comparator	Odds Ratio or SMD (95% CI)	I^2^ (%)
Weight loss ≥ 5%			
Tirzepatide 5 mg	Placebo	11.89 (9.36–15.11)	0
Tirzepatide 10 mg	Placebo	15.08 (9.26–24.57)	73
Tirzepatide 15 mg	Placebo	20.48 (13.38–31.34)	71
Weight loss ≥ 10%			
Tirzepatide 5 mg	Placebo	10.69 (8.29–13.79)	27
Tirzepatide 10 mg	Placebo	17.47 (13.90–21.96)	33
Tirzepatide 15 mg	Placebo	22.19 (18.27–26.96)	50
Weight loss ≥ 15%			
Tirzepatide 5 mg	Placebo	9.94 (7.30–13.54)	0
Tirzepatide 10 mg	Placebo	22.88 (17.06 –30.68)	0
Tirzepatide 15 mg	Placebo	25.61 (20.59–31.85)	48
Weight loss (kg)			
Tirzepatide 5 mg	Placebo	−1.05 (−1.24–−0.87)	22
Tirzepatide 10 mg	Placebo	−1.25 (−1.50–−1.00)	77
Tirzepatide 15 mg	Placebo	−1.80 (−2.12–−1.49)	93
WC (cm)			
Tirzepatide 5 mg	Placebo	−0.74 (−0.85–−0.63)	0
Tirzepatide 10 mg	Placebo	−0.91 (−1.08–−0.73)	63
Tirzepatide 15 mg	Placebo	−1.41 (−1.81–−1.02)	96
HbA1c (%)			
Tirzepatide 5 mg	Placebo	−1.45 (−1.68–−1.21)	69
Tirzepatide 10 mg	Placebo	−1.59 (−1.82–−1.37)	62
Tirzepatide 15 mg	Placebo	−1.56 (−1.85–−1.27)	91
SBP (mmHg)			
Tirzepatide 5 mg	Placebo	−0.38 (−0.53–−0.23)	37
Tirzepatide 10 mg	Placebo	−0.44 (−0.63–−0.25)	63
Tirzepatide 15 mg	Placebo	−0.55 (−0.74–−0.36)	74
DBP (mmHg)			
Tirzepatide 5 mg	Placebo	−0.33 (−0.57–−0.09)	70
Tirzepatide 10 mg	Placebo	−0.29 (−0.51–−0.07)	77
Tirzepatide 15 mg	Placebo	−0.42 (−0.54–−0.29)	55

Abbreviations: CI: confidence interval; WC: waist circumference; HbA1c: hemoglobin A1c; SBP: systolic blood pressure; DBP: diastolic blood pressure. Notes: Odds ratios (ORs) are reported for categorical outcomes (≥5%, ≥10%, and ≥15% weight loss). Standardized mean differences (SMDs) are reported for continuous outcomes including weight change, WC, HbA1c, SBP, and DBP. Higher I^2^ values indicate greater heterogeneity among the included studies.

**Table 4:** Meta-analysis results for tirzepatide versus GLP-1 RAs, and basal insulin for efficacy outcomes.

Intervention	Comparator	Odds Ratio (95% CI)	I^2^ (%)
Weight loss ≥ 5%			
Tirzepatide 5 mg	GLP-1 RAs	3.89 (1.10–13.77)	95
	Basal insulin	19.94 (16.25–24.47)	36
Tirzepatide 10 mg	GLP-1 RAs	10.63 (0.90–125.99)	98
	Basal insulin	40.62 (28.28–58.32)	64
Tirzepatide 15 mg	GLP-1 RAs	16.82 (1.07–263.37)	98
	Basal insulin	54.77 (33.35–89.95)	79
Weight loss ≥ 10%			
Tirzepatide 5 mg	GLP-1 RAs	3.59 (0.85–15.14)	90
	Basal insulin	21.81 (10.09– 47.15)	82
Tirzepatide 10 mg	GLP-1 RAs	8.15 (2.15–30.96)	90
	Basal insulin	43.70 (20.44– 93.41)	82
Tirzepatide 15 mg	GLP-1 RAs	12.20 (2.60–57.27)	91
	Basal insulin	68.64 (29.50–159.68)	86
Weight loss ≥ 15%			
Tirzepatide 5 mg	GLP-1 RAs	4.72 (0.86 –25.85)	73
	Basal insulin	56.38 (24.19–131.42)	0
Tirzepatide 10 mg	GLP-1 RAs	11.43 (2.31–56.54)	74
	Basal insulin	126.42 (54.73–292.04)	2
Tirzepatide 15 mg	GLP-1 RAs	20.25 (3.64–112.56)	76
	Basal insulin	208.36 (90.58–479.32)	0
Weight loss (kg)			
Tirzepatide 5 mg	GLP-1 RAs	−0.55 (−1.07–−0.04)	93
	Basal insulin	−1.51 (−1.74–−1.29)	87
Tirzepatide 10 mg	GLP-1 RAs	−1.06 (−1.66– −0.46)	96
	Basal insulin	−1.96 (−2.24–−1.69)	89
Tirzepatide 15 mg	GLP-1 RAs	−1.42 (−2.10–−0.74)	97
	Basal insulin	−2.23 (−2.58–−1.89)	94
WC (cm)			
Tirzepatide 5 mg	GLP-1 RAs	−0.71 (−1.27–−0.16)	84
	Basal insulin	NA	0
Tirzepatide 10 mg	GLP-1 RAs	−1.01 (−1.41–−0.61)	67
	Basal insulin	NA	31
Tirzepatide 15 mg	GLP-1 RAs	−1.43 (−1.97–−0.88)	81
	Basal insulin	NA	33
HbA1c (%)			
Tirzepatide 5 mg	GLP-1 RAs	−0.60 (−1.13–−0.06)	95
	Basal insulin	−0.78 (−1.11–−0.44)	93
Tirzepatide 10 mg	GLP-1 RAs	−0.84 (−1.29–−0.39)	93
	Basal insulin	−0.98 (−1.33–−0.63)	94
Tirzepatide 15 mg	GLP-1 RAs	−1.05 (−1.60–−0.50)	95
	Basal insulin	−1.09 (−1.44–−0.74)	94
SBP (mmHg)			
Tirzepatide 5 mg	GLP-1 RAs	−0.28 (−0.60–0.04)	55
	Basal insulin	−0.43 (−0.59–−0.27)	76
Tirzepatide 10 mg	GLP-1 RAs	−0.31 (−0.91–0.29)	86
	Basal insulin	−0.54 (−0.74–−0.34)	83
Tirzepatide 15 mg	GLP-1 RAs	−0.38 (−1.18–0.41)	92
	Basal insulin	−0.46 (−0.54–−0.38)	0
DBP (mmHg)			
Tirzepatide 5 mg	GLP-1 RAs	−0.18 (−0.65–0.28)	78
	Basal insulin	−0.25 (−0.33–−0.17)	0
Tirzepatide 10 mg	GLP-1 RAs	−0.20 (−0.81–0.42)	87
	Basal insulin	−0.30 (−0.42–−0.17)	59
Tirzepatide 15 mg	GLP-1 RAs	−0.32 (−1.06–0.43)	91
	Basal insulin	−0.19 (−0.32–−0.07)	56

Abbreviations: CI: confidence interval; GLP-1 RAs: glucagon-like peptide-1 receptor agonists; WC: waist circumference; HbA1c: hemoglobin A1c; SBP: systolic blood pressure; DBP: diastolic blood pressure, NA: not available. Notes: Odds ratios are reported for categorical outcomes (≥5%, ≥10%, and ≥15% weight loss). Standardized mean differences (SMDs) are reported for continuous outcomes including weight change, WC, HbA1c, SBP, and DBP. Higher I^2^ values indicate greater heterogeneity among included studies.

**Table 5:** Meta-analysis results for tirzepatide versus placebo for safety outcomes.

Intervention	Comparator	Odds Ratio (95% CI)	I^2^ (%)
Adverse events lead to treatment discontinuation			
Tirzepatide 5 mg	Placebo	1.75 (1.06–2.90)	0
Tirzepatide 10 mg	Placebo	2.00 (1.15–3.47)	17
Tirzepatide 15 mg	Placebo	2.84 (1.99–4.05)	23
Serious adverse events			
Tirzepatide 5 mg	Placebo	0.94 (0.64–1.38)	0
Tirzepatide 10 mg	Placebo	0.98 (0.71–1.35)	0
Tirzepatide 15 mg	Placebo	0.94 (0.70–1.24)	0
Any adverse events			
Tirzepatide 5 mg	Placebo	1.53 (1.23–1.90)	12
Tirzepatide 10 mg	Placebo	1.36 (0.98–1.89)	63
Tirzepatide 15 mg	Placebo	1.53 (1.06–2.21)	74
Nausea			
Tirzepatide 5 mg	Placebo	3.12 (2.34–4.16)	0
Tirzepatide 10 mg	Placebo	4.38 (3.42–5.61)	0
Tirzepatide 15 mg	Placebo	4.27 (3.49–5.23)	0
Vomiting			
Tirzepatide 5 mg	Placebo	4.19 (2.43–7.21)	0
Tirzepatide 10 mg	Placebo	4.61 (2.83–7.49)	4
Tirzepatide 15 mg	Placebo	6.76 (4.71–9.70)	0
Diarrhea			
Tirzepatide 5 mg	Placebo	2.25 (1.28–3.97)	56
Tirzepatide 10 mg	Placebo	2.59 (1.79–3.76)	45
Tirzepatide 15 mg	Placebo	3.26 (2.57–4.14)	33
Decreased appetite			
Tirzepatide 5 mg	Placebo	3.45 (2.18–5.48)	0
Tirzepatide 10 mg	Placebo	4.60 (3.09–6.84)	0
Tirzepatide 15 mg	Placebo	4.00 (2.49–6.41)	23

Abbreviations: CI: confidence interval. Notes: Higher I^2^ values indicate greater heterogeneity among included studies.

**Table 6:** Meta-analysis results for tirzepatide versus GLP-1 RAs, and basal insulin for safety outcomes.

Intervention	Comparator	Odds Ratio (95% CI)	I^2^ (%)
Adverse events lead to treatment discontinuation			
Tirzepatide 5 mg	GLP-1 RAs	1.34 (0.85–2.13)	0
	Basal insulin	2.28 (1.63–3.20)	21
Tirzepatide 10 mg	GLP-1 RAs	1.73 (1.00–2.99)	44
	Basal insulin	3.22 (1.50–6.88)	76
Tirzepatide 15 mg	GLP-1 RAs	2.17 (1.41–3.32)	0
	Basal insulin	4.00 (2.19–7.32)	68
Serious adverse events			
Tirzepatide 5 mg	GLP-1 RAs	0.96 (0.26–3.50)	79
	Basal insulin	0.77 (0.54–1.10)	45
Tirzepatide 10 mg	GLP-1 RAs	1.20 (0.56–2.57)	44
	Basal insulin	0.75 (0.59–0.96)	0
Tirzepatide 15 mg	GLP-1 RAs	0.97 (0.33–2.81)	71
	Basal insulin	0.70 (0.49–0.98)	38
Any adverse events			
Tirzepatide 5 mg	GLP-1 RAs	1.03 (0.82–1.30)	0
	Basal insulin	1.61 (1.14–2.27)	75
Tirzepatide 10 mg	GLP-1 RAs	1.17 (0.93–1.47)	0
	Basal insulin	2.32 (1.18–4.56)	87
Tirzepatide 15 mg	GLP-1 RAs	1.32 (1.04–1.67)	0
	Basal insulin	2.50 (1.58–3.94)	80
Nausea			
Tirzepatide 5 mg	GLP-1 RAs	1.00 (0.73–1.36)	35
	Basal insulin	8.32 (5.33–12.99)	20
Tirzepatide 10 mg	GLP-1 RAs	1.30 (0.58–2.95)	77
	Basal insulin	14.92 (8.67–25.69)	55
Tirzepatide 15 mg	GLP-1 RAs	1.73 (1.03–2.91)	57
	Basal insulin	17.99 (11.52–28.10)	28
Vomiting			
Tirzepatide 5 mg	GLP-1 RAs	1.37 (0.34–5.56)	76
	Basal insulin	4.92 (2.89–8.36)	0
Tirzepatide 10 mg	GLP-1 RAs	1.51 (0.72–3.17)	40
	Basal insulin	9.00 (5.11–15.86)	22
Tirzepatide 15 mg	GLP-1 RAs	3.03 (0.88–10.42)	80
	Basal insulin	10.23 (5.27–19.88)	49
Diarrhea			
Tirzepatide 5 mg	GLP-1 RAs	1.60 (0.93–2.77)	51
	Basal insulin	6.53 (2.53–16.86)	80
Tirzepatide 10 mg	GLP-1 RAs	1.48 (1.07–2.04)	0
	Basal insulin	9.36 (3.37–25.98)	81
Tirzepatide 15 mg	GLP-1 RAs	1.44 (1.01–2.06)	0
	Basal insulin	8.60 (3.03–24.46)	80
Decreased appetite			
Tirzepatide 5 mg	GLP-1 RAs	2.38 (1.15–4.94)	53
	Basal insulin	23.25 (11.34–47.70)	31
Tirzepatide 10 mg	GLP-1 RAs	2.57 (1.12–5.89)	64
	Basal insulin	38.06 (15.43–93.90)	40
Tirzepatide 15 mg	GLP-1 RAs	3.21 (1.37–7.48)	70
	Basal insulin	46.87 (15.57–141.07)	46

Abbreviations: CI: confidence interval; GLP-1 RAs: glucagon-like peptide-1 receptor agonists. Notes: Higher I^2^ values indicate greater heterogeneity among included studies.

## Supplementary Materials

The following supporting information can be downloaded at: https://www.mdpi.com/article/10.3390/ph18050668/s1, Table S1: Risk of Bias version 2 (RoB v2) for assessment of clinical trial studies; Figure S1: Forest plot which demonstrates the proportion of patients achieving at least 5% weight loss in different dose of tirzepatide when compared with placebo (A), glucagon like peptide-1 receptor agonist (GLP-1 RAs) (B), and insulin (C); Figure S2: Forest plot which demonstrates the proportion of patients achieving at least 10% weight loss in different dose of tirzepatide when compared with placebo (A), glucagon like peptide-1 receptor agonist (GLP-1 RAs) (B), and insulin (C); Figure S3: Forest plot which demonstrates the proportion of patients achieving at least 15% weight loss in different dose of tirzepatide when compared with placebo (A), glucagon like peptide-1 receptor agonist (GLP-1 RAs) (B), and insulin (C); Figure S4: Forest plot which demonstrates the change of body weight (kg) in different dose of tirzepatide when compared with placebo (A), glucagon like peptide-1 receptor agonist (GLP-1 RAs) (B), and insulin (C); Figure S5: Forest plot that demonstrates the change of waist circumference in different dose of tirzepatide when compared with placebo (A), glucagon like peptide-1 receptor agonist (GLP-1 RAs) (B); Figure S6: Forest plot which demonstrates the change of HbA1c in different dose of tirzepatide when compared with placebo (A), glucagon like peptide-1 receptor agonist (GLP-1 RAs) (B), and insulin (C); Figure S7: Forest plot which demonstrates the change of DBP in different dose of tirzepatide when compared with placebo (A), glucagon like peptide-1 receptor agonist (GLP-1 RAs) (B), and insulin (C); Figure S8: Forest plot which demonstrates the change of SBP in different dose of tirzepatide when compared with placebo (A), glucagon like peptide-1 receptor agonist (GLP-1 RAs) (B), and insulin (C); Figure S9: Forest plot which demonstrates the proportion of patients with any adverse events in different dose of tirzepatide when compared with placebo (A), glucagon like peptide-1 receptor agonist (GLP-1 RAs) (B), and insulin (C); Figure S10: Forest plot which demonstrates the proportion of patients with severe adverse events in different dose of tirzepatide when compared with placebo (A), glucagon like peptide-1 receptor agonist (GLP-1 RAs) (B), and insulin (C); Figure S11: Forest plot which demonstrates the proportion of patients with discontinuation of treatment due to adverse events in different dose of tirzepatide when compared with placebo (A), glucagon like peptide-1 receptor agonist (GLP-1 RAs) (B), and insulin (C); Figure S12: Forest plot which demonstrates the proportion of patients with nausea in different dose of tirzepatide when compared with placebo (A), glucagon like peptide-1 receptor agonist (GLP-1 RAs) (B), and insulin (C); Figure S13: Forest plot which demonstrates the proportion of patients with vomiting in different dose of tirzepatide when compared with placebo (A), glucagon like peptide-1 receptor agonist (GLP-1 RAs) (B), and insulin (C); Figure S14: Forest plot which demonstrates the proportion of patients with diarrhea in different dose of tirzepatide when compared with placebo (A), glucagon like peptide-1 receptor agonist (GLP-1 RAs) (B), and insulin (C); Figure S15: Forest plot which demonstrates the proportion of patients with decreased appetite in different dose of tirzepatide when compared with placebo (A), glucagon like peptide-1 receptor agonist (GLP-1 RAs) (B), and insulin (C).
