# Supplementary material for: The Efficacy and Safety of Tirzepatide in Patients with Diabetes and/or Obesity: Systematic Review and Meta-Analysis of Randomized Clinical Trials
Source: Pharmaceuticals (Basel). 2025 Apr 30;18(5):668. doi: 10.3390/ph18050668 (PMC12114739; doi:10.3390/ph18050668)
Supplement: Supplementary file 1 [file pharmaceuticals-18-00668-s001.zip › pharmaceuticals-3553651-supplementary.pdf]

Supplemental table S1. Risk of Bias version 2 (RoB v2) for assessment of clinical trial studies.

Study	D1	D2	D3	D4	D5	Overall
SURMOUNT-4	+	+	+	+	+	+
SURMOUNT-3	+	+	+	+	+	+
SURMOUNT-2	+	+	+	+	+	+
SURMOUNT-1	+	+	+	+	+	+
SURPASS-AP-Combo	+	+	+	+	+	+
SURPASS J-mono	+	+	+	+	+	+
SURPASS-6	+	+	+	+	+	+
SURPASS-5	+	+	+	+	+	+
SURPASS-4	+	+	+	+	+	+
SURPASS-3	?	+	+	+	+	?
SURPASS-2	-	+	+	+	+	-
SURPASS-1	+	+	+	+	+	+
NCT03311724	?	+	+	+	+	?
NCT03131687	+	+	-	+	+	-

+	Low risk
?	Some concerns
-	High risk

(D1) process of randomization; (D2) deviations from intended interventions; (D3) missing outcome data; (D4) outcome measurement; and (D5) selection of the reported result.

Supplementary Figure S1. Forest plot which demonstrates the proportion of patients achieving at least 5% weight loss in different dose of tirzepatide when compared with placebo (A), glucagon like peptide-1 receptor agonist (GLP-1 RAs) (B), and insulin (C).

A.

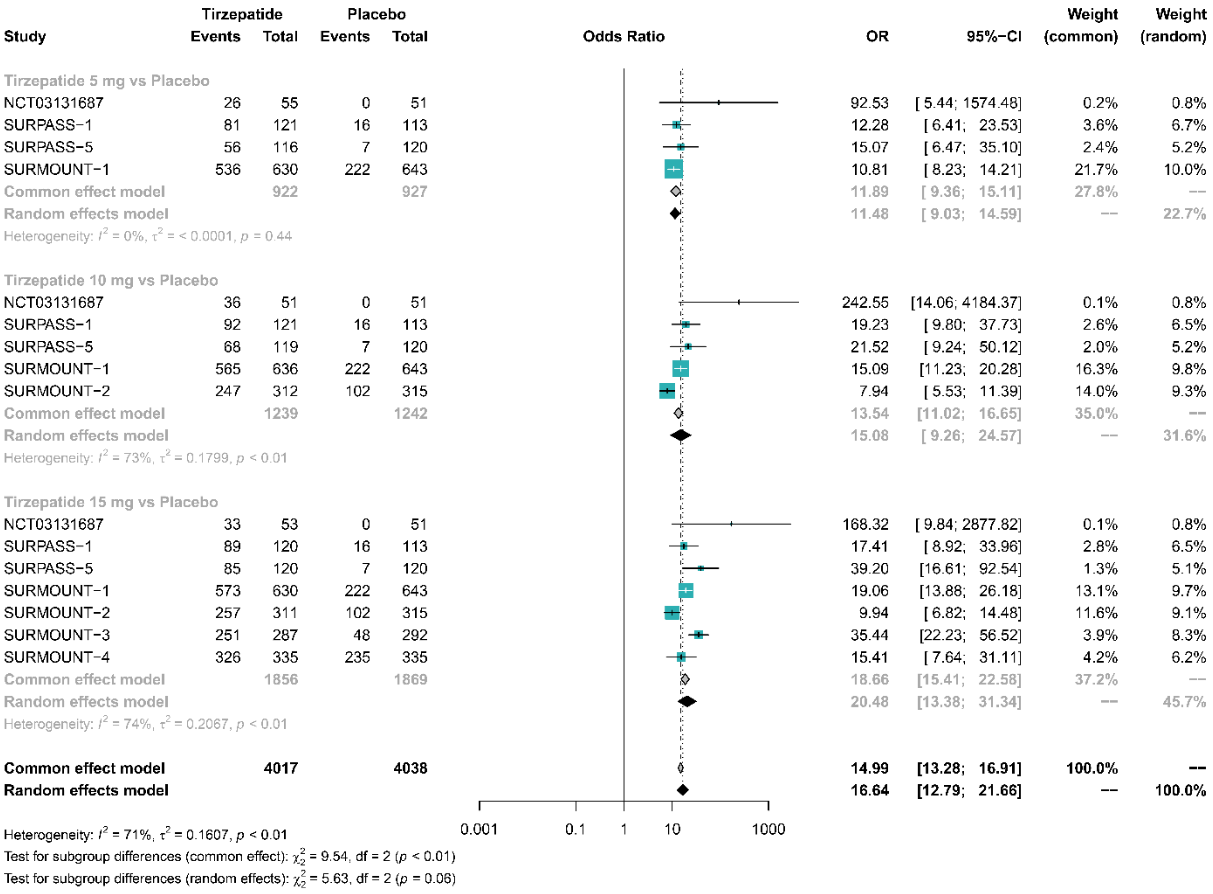

B.

Study	Tirzepatide Events	Tirzepatide Total	GLP-1 RAs Events	GLP-1 RAs Total	OR	95%-CI	Weight (common)	Weight (random)
**Tirzepatide 5 mg vs GLP-1 RAs**								
NCT03131687	26	55	12	54	3.14	[1.37; 7.21]	3.2%	13.6%
SURPASS-2	316	470	270	469	1.51	[1.16; 1.97]	44.5%	14.7%
SURPASS J-mono	96	158	17	159	12.93	[7.13; 23.47]	3.3%	14.2%
Common effect model		683		682	2.36	[1.89; 2.95]	51.0%	--
Random effects model					3.89	[1.10; 13.77]	--	42.6%
Heterogeneity: $I^2 = 95\%$, $\tau^2 = 1.1565$, $p < 0.01$								
**Tirzepatide 10 mg vs GLP-1 RAs**								
NCT03131687	36	51	54	12			0.0%	0.0%
SURPASS-2	378	469	270	469	3.06	[2.28; 4.10]	26.3%	14.7%
SURPASS J-mono	128	156	17	159	38.18	[19.97; 73.02]	1.5%	14.1%
Common effect model		676		640	4.98	[3.86; 6.41]	27.8%	--
Random effects model					10.63	[0.90; 125.99]	--	28.8%
Heterogeneity: $I^2 = 98\%$, $\tau^2 = 3.1182$, $p < 0.01$								
**Tirzepatide 15 mg vs GLP-1 RAs**								
NCT03131687	33	53	54	12			0.0%	0.0%
SURPASS-2	400	470	270	469	4.21	[3.08; 5.76]	20.2%	14.7%
SURPASS J-mono	142	159	17	159	69.77	[34.26; 142.11]	0.9%	13.9%
Common effect model		682		640	7.04	[5.37; 9.23]	21.1%	--
Random effects model					16.82	[1.07; 263.37]	--	28.6%
Heterogeneity: $I^2 = 98\%$, $\tau^2 = 3.8621$, $p < 0.01$								
**Common effect model**		**2041**		**1962**	**4.08**	**[3.55; 4.69]**	**100.0%**	**--**
**Random effects model**					**7.86**	**[2.73; 22.62]**	**--**	**100.0%**

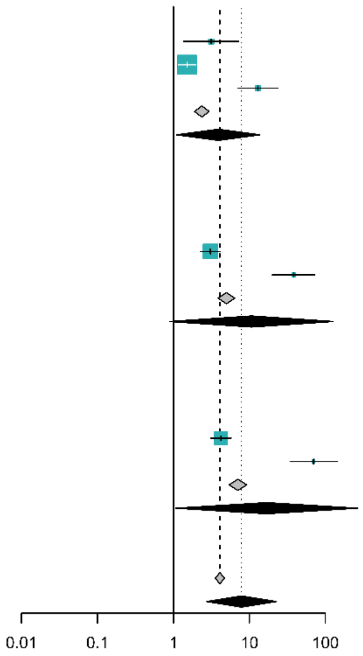

Heterogeneity: $I^2 = 97\%$, $\tau^2 = 1.9571$, $p < 0.01$
Test for subgroup differences (common effect): $\chi^2_2 = 41.13$, df = 2 ($p < 0.01$)
Test for subgroup differences (random effects): $\chi^2_2 = 1.19$, df = 2 ($p = 0.55$)

C.

Study	Tirzepatide Events	Tirzepatide Total	Insulin Events	Insulin Total	OR	95%-CI	Weight (common)	Weight (random)
**Tirzepatide 5 mg vs Insulin**								
SURPASS-3	233	358	22	360	28.64	[17.67; 46.42]	8.9%	8.3%
SURPASS-4	205	326	78	978	19.55	[14.16; 26.99]	16.7%	9.2%
SURPASS-6	151	242	71	708	14.89	[10.41; 21.28]	15.7%	9.0%
SURPASS-AP-Combo	127	230	12	220	21.37	[11.30; 40.42]	6.3%	7.3%
Common effect model		1156		2266	19.94	[16.25; 24.47]	47.6%	--
Random effects model					19.75	[15.07; 25.90]	--	33.8%
Heterogeneity: $I^2 = 36\%$, $\tau^2 = 0.0292$, $p = 0.20$								
**Tirzepatide 10 mg vs Insulin**								
SURPASS-3	293	360	22	360	67.19	[40.49; 111.48]	4.7%	8.1%
SURPASS-4	249	321	78	978	39.90	[28.13; 56.61]	10.0%	9.1%
SURPASS-6	180	238	71	708	27.84	[18.96; 40.89]	10.1%	8.9%
SURPASS-AP-Combo	159	228	12	220	39.94	[20.92; 76.27]	4.3%	7.2%
Common effect model		1147		2266	40.18	[32.33; 49.93]	29.1%	--
Random effects model					40.62	[28.28; 58.32]	--	33.3%
Heterogeneity: $I^2 = 59\%$, $\tau^2 = 0.0807$, $p = 0.06$								
**Tirzepatide 15 mg vs Insulin**								
SURPASS-3	310	359	22	360	97.20	[57.44; 164.49]	3.5%	8.0%
SURPASS-4	285	334	78	978	67.11	[45.84; 98.26]	6.7%	8.9%
SURPASS-6	183	236	71	708	30.98	[20.93; 45.84]	9.2%	8.8%
SURPASS-AP-Combo	166	229	12	220	45.67	[23.84; 87.50]	3.9%	7.2%
Common effect model		1158		2266	53.72	[42.81; 67.41]	23.3%	--
Random effects model					54.77	[33.35; 89.95]	--	32.9%
Heterogeneity: $I^2 = 79\%$, $\tau^2 = 0.1945$, $p < 0.01$								
**Common effect model**		**3461**		**6798**	**33.69**	**[29.77; 38.13]**	**100.0%**	**--**
**Random effects model**					**35.65**	**[25.89; 49.09]**	**--**	**100.0%**

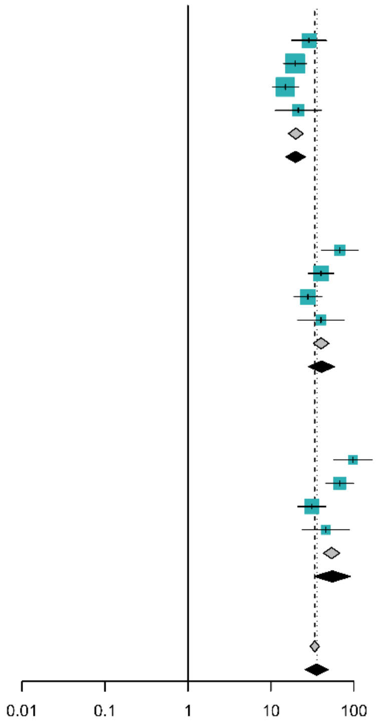

Heterogeneity: $I^2 = 85\%$, $\tau^2 = 0.2612$, $p < 0.01$
Test for subgroup differences (common effect): $\chi^2_2 = 43.97$, df = 2 ($p < 0.01$)
Test for subgroup differences (random effects): $\chi^2_2 = 17.35$, df = 2 ($p < 0.01$)

Supplementary Figure S2. Forest plot which demonstrates the proportion of patients achieving at least 10% weight loss in different dose of tirzepatide when compared with placebo (A), glucagon like peptide-1 receptor agonist (GLP-1 RAs) (B), and insulin (C).

A.

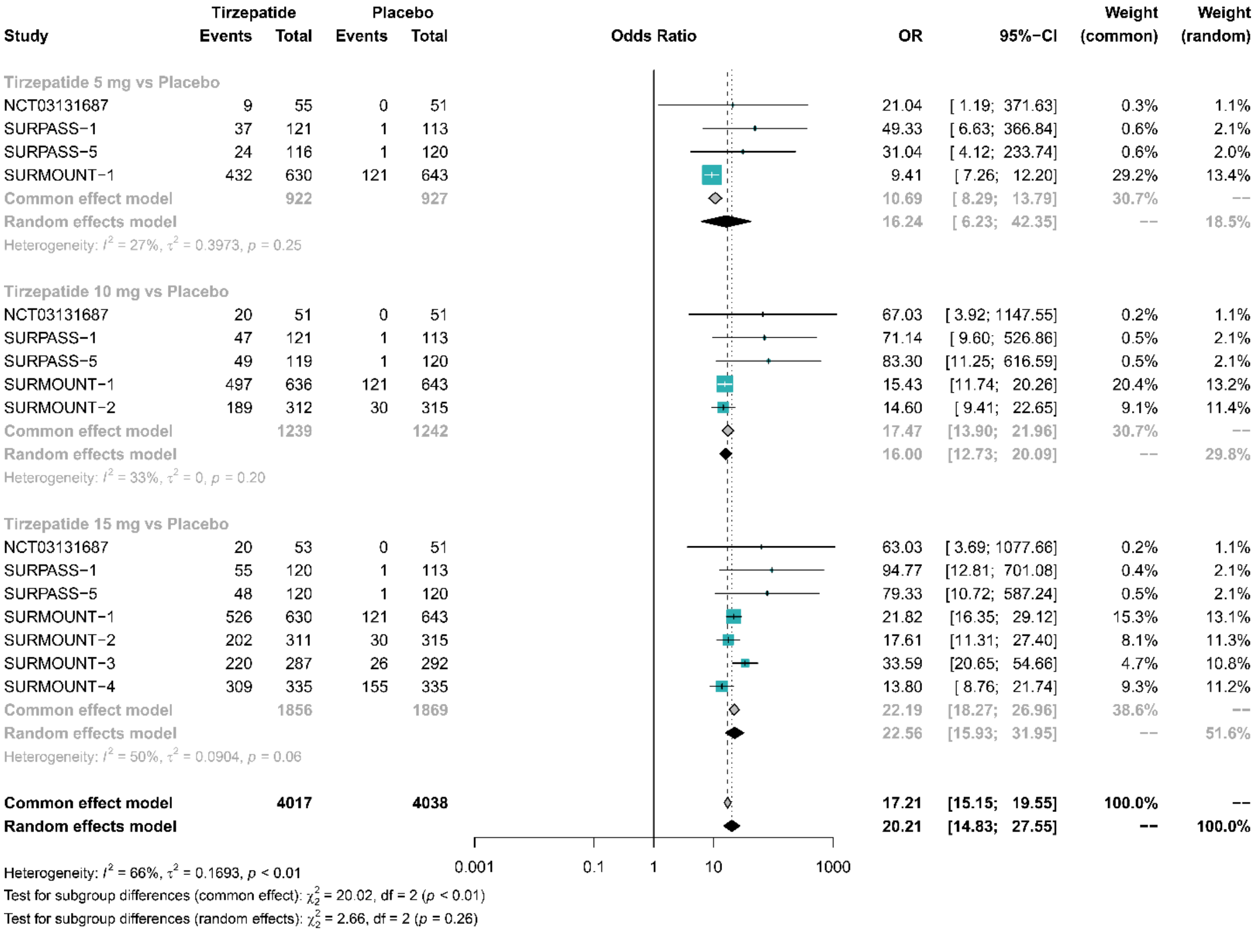

B.

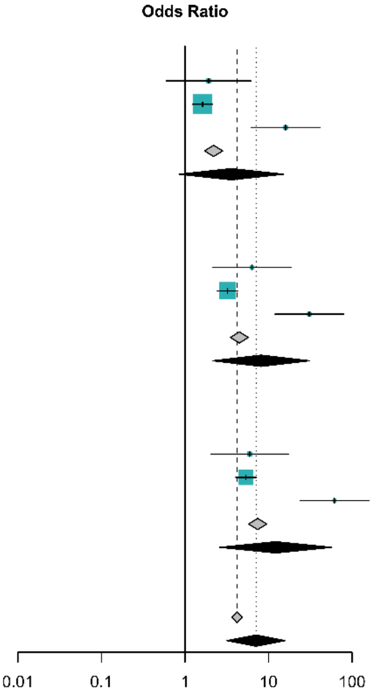

Study	Tirzepatide Events	Tirzepatide Total	GLP-1 RAs Events	GLP-1 RAs Total	OR	95%-CI	Weight (common)	Weight (random)
**Tirzepatide 5 mg vs GLP-1 RAs**								
NCT03131687	9	55	5	54	1.92	[0.60; 6.15]	2.2%	10.0%
SURPASS-2	165	470	117	468	1.62	[1.22; 2.15]	39.6%	12.4%
SURPASS J-mono	54	158	5	159	15.99	[6.19; 41.33]	1.7%	10.7%
Common effect model		683		681	2.20	[1.71; 2.83]	43.5%	--
Random effects model					3.59	[0.85; 15.14]	--	33.1%
Heterogeneity: $I^2 = 90\%$, $\tau^2 = 1.4292$, $p < 0.01$								
**Tirzepatide 10 mg vs GLP-1 RAs**								
NCT03131687	20	51	5	54	6.32	[2.15; 18.59]	1.5%	10.3%
SURPASS-2	243	469	117	468	3.23	[2.45; 4.25]	29.4%	12.4%
SURPASS J-mono	78	156	5	159	30.80	[11.98; 79.19]	1.3%	10.7%
Common effect model		676		681	4.48	[3.49; 5.74]	32.2%	--
Random effects model					8.15	[2.15; 30.96]	--	33.4%
Heterogeneity: $I^2 = 90\%$, $\tau^2 = 1.2164$, $p < 0.01$								
**Tirzepatide 15 mg vs GLP-1 RAs**								
NCT03131687	20	53	5	54	5.94	[2.03; 17.40]	1.6%	10.3%
SURPASS-2	301	469	117	468	5.38	[4.06; 7.12]	21.8%	12.4%
SURPASS J-mono	106	159	5	159	61.60	[23.83; 159.23]	0.9%	10.7%
Common effect model		681		681	7.42	[5.77; 9.54]	24.3%	--
Random effects model					12.20	[2.60; 57.27]	--	33.4%
Heterogeneity: $I^2 = 91\%$, $\tau^2 = 1.6890$, $p < 0.01$								
**Common effect model**		**2040**		**2043**	**4.20**	**[3.64; 4.85]**	**100.0%**	**--**
**Random effects model**					**7.10**	**[3.19; 15.79]**	**--**	**100.0%**

Heterogeneity: $I^2 = 92\%$, $\tau^2 = 1.3201$, $p < 0.01$
Test for subgroup differences (common effect): $\chi^2_2 = 45.22$, df = 2 ($p < 0.01$)
Test for subgroup differences (random effects): $\chi^2_2 = 1.37$, df = 2 ($p = 0.50$)

C.

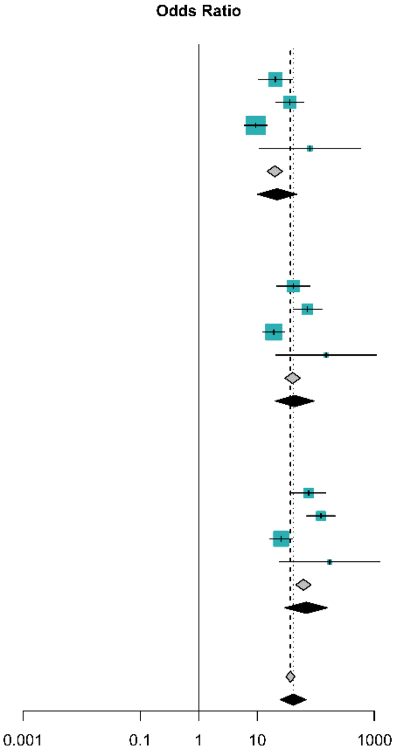

Study	Tirzepatide Events	Tirzepatide Total	Insulin Events	Insulin Total	OR	95%-CI	Weight (common)	Weight (random)
**Tirzepatide 5 mg vs Insulin**								
SURPASS-3	132	358	10	359	20.38	[10.49; 39.61]	11.5%	9.3%
SURPASS-4	117	326	15	978	35.94	[20.57; 62.79]	8.8%	9.7%
SURPASS-6	78	242	34	708	9.43	[6.09; 14.60]	21.4%	10.2%
SURPASS-AP-Combo	61	230	1	220	79.05	[10.85; 576.04]	1.4%	4.1%
Common effect model		1156		2265	19.97	[14.75; 27.03]	43.1%	--
Random effects model					21.81	[10.09; 47.15]	--	33.3%
Heterogeneity: $I^2 = 82\%$, $\tau^2 = 0.4383$, $p < 0.01$								
**Tirzepatide 10 mg vs Insulin**								
SURPASS-3	195	360	10	359	41.25	[21.28; 79.95]	8.4%	9.3%
SURPASS-4	170	321	15	978	72.28	[41.49; 125.92]	6.4%	9.8%
SURPASS-6	117	238	34	708	19.17	[12.49; 29.41]	15.9%	10.2%
SURPASS-AP-Combo	93	228	1	220	150.87	[20.79; 1095.00]	1.1%	4.1%
Common effect model		1147		2265	40.22	[29.73; 54.42]	31.7%	--
Random effects model					43.70	[20.44; 93.41]	--	33.4%
Heterogeneity: $I^2 = 82\%$, $\tau^2 = 0.4238$, $p < 0.01$								
**Tirzepatide 15 mg vs Insulin**								
SURPASS-3	245	358	10	359	75.67	[38.84; 147.42]	5.8%	9.3%
SURPASS-4	219	334	15	978	122.26	[70.00; 213.54]	4.8%	9.7%
SURPASS-6	133	236	34	708	25.60	[16.65; 39.35]	13.6%	10.2%
SURPASS-AP-Combo	101	229	1	220	172.80	[23.82; 1253.68]	1.0%	4.1%
Common effect model		1157		2265	61.61	[45.54; 83.35]	25.2%	--
Random effects model					68.64	[29.50; 159.68]	--	33.3%
Heterogeneity: $I^2 = 86\%$, $\tau^2 = 0.5508$, $p < 0.01$								
**Common effect model**		**3460**		**6795**	**36.87**	**[31.00; 43.86]**	**100.0%**	**--**
**Random effects model**					**41.14**	**[24.87; 68.06]**	**--**	**100.0%**

Heterogeneity: $I^2 = 87\%$, $\tau^2 = 0.5958$, $p < 0.01$
Test for subgroup differences (common effect): $\chi^2_2 = 27.15$, df = 2 ($p < 0.01$)
Test for subgroup differences (random effects): $\chi^2_2 = 3.99$, df = 2 ($p = 0.14$)

Supplementary Figure S3. Forest plot which demonstrates the proportion of patients achieving at least 15% weight loss in different dose of tirzepatide when compared with placebo (A), glucagon like peptide-1 receptor agonist (GLP-1 RAs) (B), and insulin (C).

A.

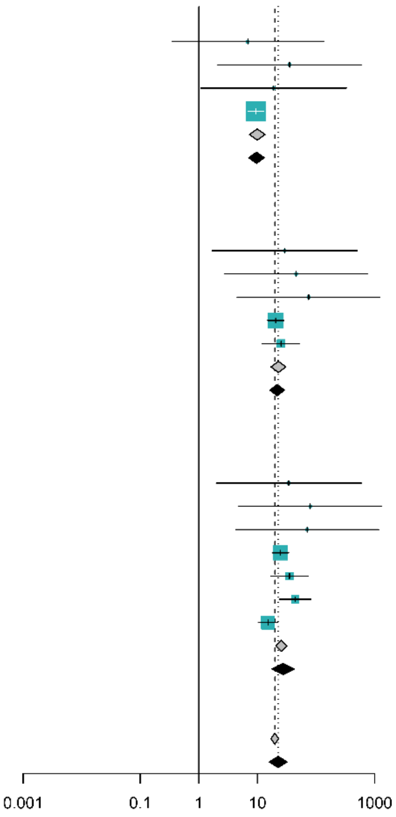

Study	Tirzepatide Events	Tirzepatide Total	Placebo Events	Placebo Total	OR	95%-CI	Weight (common)	Weight (random)
**Tirzepatide 5 mg vs Placebo**								
NCT03131687	3	55	0	51	6.87	[0.35; 136.28]	0.5%	1.3%
SURPASS−1	16	121	0	113	35.50	[2.10; 599.17]	0.5%	1.4%
SURPASS−5	8	116	0	120	18.88	[1.08; 330.98]	0.5%	1.4%
SURMOUNT−1	302	630	57	643	9.47	[6.92; 12.95]	30.8%	14.6%
Common effect model		922		927	9.94	[7.30; 13.54]	32.3%	--
Random effects model					9.66	[7.10; 13.15]	--	18.7%
Heterogeneity: $I^2 = 0\%$, $\tau^2 = 0$, $p = 0.78$								
**Tirzepatide 10 mg vs Placebo**								
NCT03131687	11	51	0	51	29.25	[1.67; 511.30]	0.4%	1.4%
SURPASS−1	20	121	0	113	45.85	[2.74; 767.76]	0.5%	1.4%
SURPASS−5	28	119	0	120	75.07	[4.52; 1245.83]	0.4%	1.4%
SURMOUNT−1	424	636	57	643	20.56	[14.96; 28.26]	19.8%	14.6%
SURMOUNT−2	124	312	8	315	25.31	[12.10; 52.93]	5.0%	9.5%
Common effect model		1239		1242	22.88	[17.06; 30.68]	26.1%	--
Random effects model					21.77	[16.33; 29.02]	--	28.3%
Heterogeneity: $I^2 = 0\%$, $\tau^2 = 0$, $p = 0.85$								
**Tirzepatide 15 mg vs Placebo**								
NCT03131687	13	53	0	51	34.33	[1.98; 595.05]	0.4%	1.4%
SURPASS−1	31	120	0	113	79.89	[4.82; 1323.62]	0.4%	1.4%
SURPASS−5	27	120	0	120	70.88	[4.27; 1177.24]	0.4%	1.4%
SURMOUNT−1	445	630	57	643	24.73	[17.93; 34.11]	17.4%	14.5%
SURMOUNT−2	149	311	8	315	35.30	[16.90; 73.71]	4.3%	9.6%
SURMOUNT−3	188	287	12	292	44.31	[23.67; 82.94]	4.3%	10.8%
SURMOUNT−4	282	335	87	335	15.17	[10.35; 22.22]	14.4%	13.8%
Common effect model		1856		1869	25.61	[20.59; 31.85]	41.6%	--
Random effects model					27.47	[17.70; 42.64]	--	53.0%
Heterogeneity: $I^2 = 48\%$, $\tau^2 = 0.1483$, $p = 0.07$								
**Common effect model**		**4017**		**4038**	**19.84**	**[17.05; 23.09]**	**100.0%**	**--**
**Random effects model**					**22.65**	**[15.96; 32.14]**	**--**	**100.0%**

Heterogeneity: $I^2 = 60\%$, $\tau^2 = 0.1923$, $p < 0.01$

Test for subgroup differences (common effect): $\chi^2_2 = 25.37$, df = 2 ($p < 0.01$)

Test for subgroup differences (random effects): $\chi^2_2 = 20.30$, df = 2 ($p < 0.01$)

B.

Study	Tirzepatide Events	Tirzepatide Total	GLP-1 RAs Events	GLP-1 RAs Total	OR	95%-CI	Weight (common)	Weight (random)
**Tirzepatide 5 mg vs GLP-1 RAs**								
NCT03131687	3	55	1	54	3.06	[0.31; 30.35]	1.0%	8.1%
SURPASS−2	70	470	40	468	1.87	[1.24; 2.83]	37.0%	15.6%
SURPASS J−mono	26	158	1	159	31.12	[4.17; 232.41]	0.9%	9.1%
Common effect model		683		681	2.58	[1.77; 3.77]	39.0%	--
Random effects model					4.72	[0.86; 25.85]	--	32.7%
Heterogeneity: $I^2 = 73\%$, $\tau^2 = 1.5873$, $p = 0.03$								
**Tirzepatide 10 mg vs GLP-1 RAs**								
NCT03131687	11	51	1	54	14.57	[1.81; 117.59]	0.8%	8.8%
SURPASS−2	127	469	40	468	3.97	[2.71; 5.83]	31.7%	15.6%
SURPASS J−mono	40	156	1	159	54.48	[7.38; 402.07]	0.8%	9.2%
Common effect model		676		681	5.45	[3.81; 7.79]	33.3%	--
Random effects model					11.43	[2.31; 56.54]	--	33.6%
Heterogeneity: $I^2 = 74\%$, $\tau^2 = 1.3955$, $p = 0.02$								
**Tirzepatide 15 mg vs GLP-1 RAs**								
NCT03131687	13	53	1	54	17.23	[2.16; 137.18]	0.8%	8.9%
SURPASS−2	185	469	40	468	6.97	[4.80; 10.12]	26.3%	15.6%
SURPASS J−mono	71	159	1	159	127.48	[17.41; 933.37]	0.6%	9.2%
Common effect model		681		681	9.88	[6.97; 14.00]	27.7%	--
Random effects model					20.25	[3.64; 112.56]	--	33.7%
Heterogeneity: $I^2 = 76\%$, $\tau^2 = 1.6850$, $p = 0.01$								
**Common effect model**		**2040**		**2043**	**5.56**	**[4.53; 6.82]**	**100.0%**	**--**
**Random effects model**					**10.17**	**[4.04; 25.59]**	**--**	**100.0%**

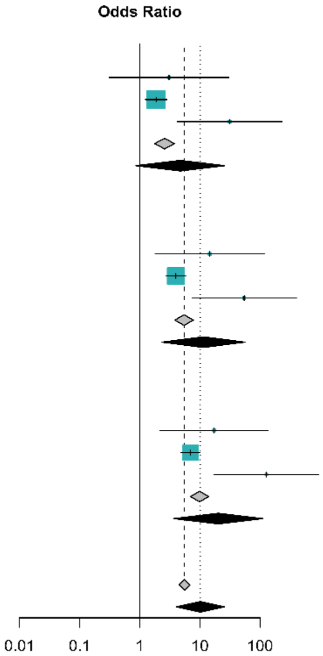

Heterogeneity: $I^2 = 83\%$, $\tau^2 = 1.3797$, $p < 0.01$
Test for subgroup differences (common effect): $\chi^2_2 = 26.10$, df = 2 ($p < 0.01$)
Test for subgroup differences (random effects): $\chi^2_2 = 1.42$, df = 2 ($p = 0.49$)

C.

Study	Tirzepatide Events	Tirzepatide Total	Insulin Events	Insulin Total	OR	95%-CI	Weight (common)	Weight (random)
**Tirzepatide 5 mg vs Insulin**								
SURPASS−3	44	358	0	359	101.73	[6.24; 1658.73]	5.1%	4.4%
SURPASS−4	45	326	5	978	31.16	[12.25; 79.26]	25.1%	19.7%
SURPASS−6	33	242	0	708	226.58	[13.82; 3713.71]	2.6%	4.4%
SURPASS−AP−Combo	23	230	0	220	49.94	[3.01; 827.54]	5.3%	4.4%
Common effect model		1156		2265	56.38	[24.19; 131.42]	38.1%	--
Random effects model					44.15	[18.38; 106.10]	--	33.0%
Heterogeneity: $I^2 = 0\%$, $\tau^2 = 0.0537$, $p = 0.53$								
**Tirzepatide 10 mg vs Insulin**								
SURPASS−3	99	360	0	359	273.58	[16.92; 4424.49]	4.2%	4.5%
SURPASS−4	77	321	5	978	61.41	[24.59; 153.38]	21.9%	20.1%
SURPASS−6	71	238	0	708	604.87	[37.28; 9814.21]	2.1%	4.5%
SURPASS−AP−Combo	38	228	0	220	89.13	[5.44; 1460.49]	4.9%	4.4%
Common effect model		1147		2265	126.42	[54.73; 292.04]	33.1%	--
Random effects model					106.67	[35.97; 316.31]	--	33.4%
Heterogeneity: $I^2 = 2\%$, $\tau^2 = 0.2929$, $p = 0.38$								
**Tirzepatide 15 mg vs Insulin**								
SURPASS−3	150	358	0	359	518.99	[32.14; 8379.39]	3.4%	4.5%
SURPASS−4	122	334	5	978	111.99	[45.23; 277.26]	18.8%	20.2%
SURPASS−6	96	236	0	708	973.24	[60.09; 15764.26]	1.7%	4.5%
SURPASS−AP−Combo	40	229	0	220	94.25	[5.76; 1543.14]	4.9%	4.4%
Common effect model		1157		2265	208.36	[90.58; 479.32]	28.8%	--
Random effects model					167.49	[62.97; 445.54]	--	33.6%
Heterogeneity: $I^2 = 0\%$, $\tau^2 = 0.1646$, $p = 0.39$								
**Common effect model**		**3460**		**6795**	**123.35**	**[76.44; 199.06]**	**100.0%**	**--**
**Random effects model**					**99.34**	**[52.94; 186.41]**	**--**	**100.0%**

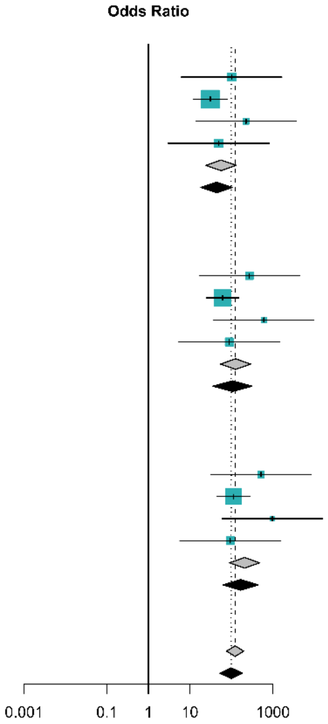

Heterogeneity: $I^2 = 16\%$, $\tau^2 = 0.2954$, $p = 0.29$
Test for subgroup differences (common effect): $\chi^2_2 = 4.73$, df = 2 ($p = 0.09$)
Test for subgroup differences (random effects): $\chi^2_2 = 4.16$, df = 2 ($p = 0.13$)

Supplementary Figure S4. Forest plot which demonstrates the change of body weight (kg) in different dose of tirzepatide when compared with placebo (A), glucagon like peptide-1 receptor agonist (GLP-1 RAs) (B), and insulin (C).

A.

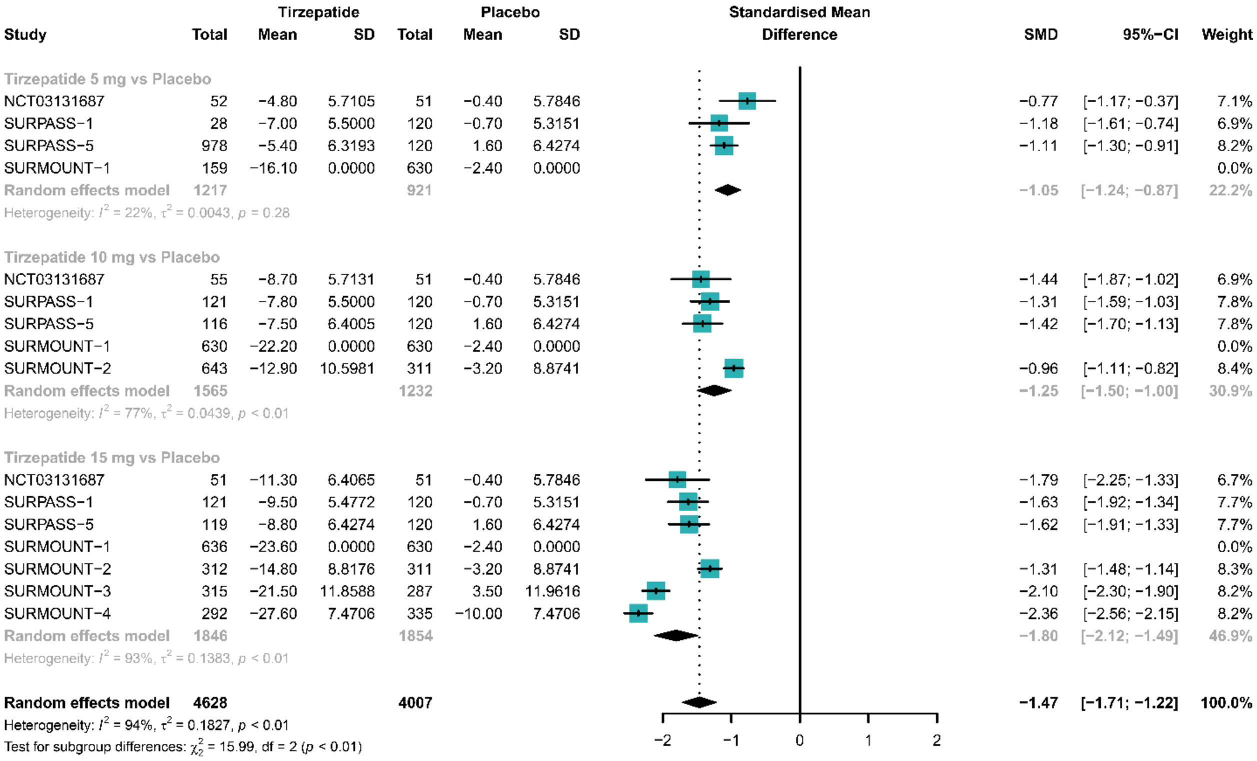

B.

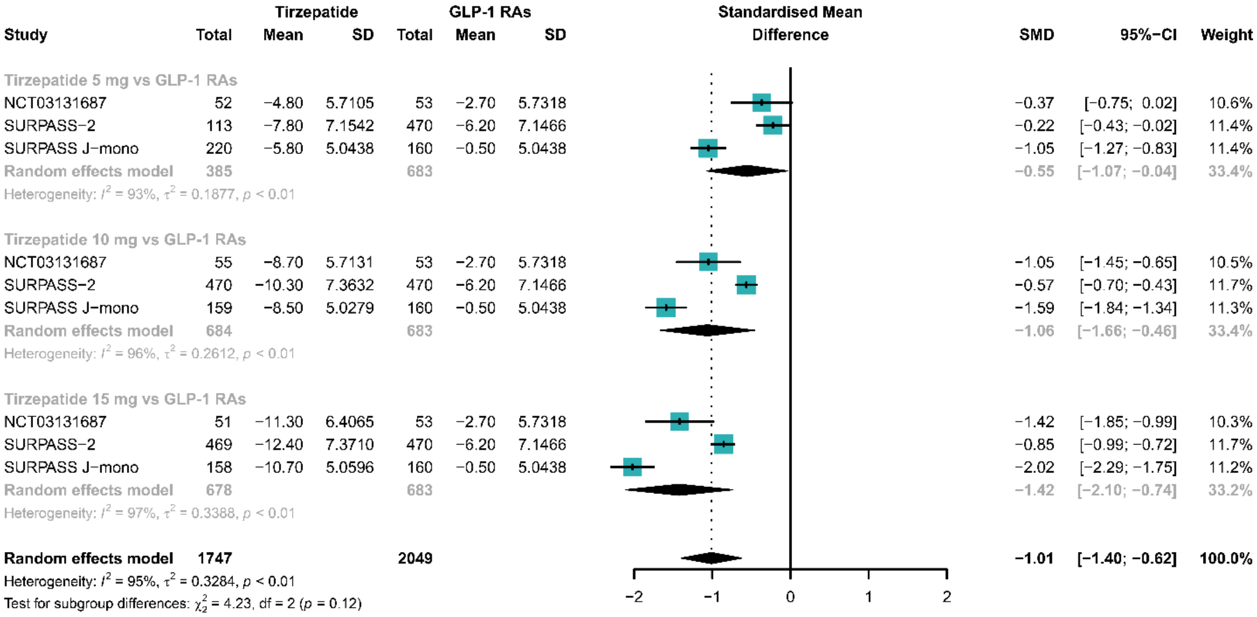
Tirzepatide
GLP-1 RAs
Standardised Mean Difference
Study Total Mean SD Total Mean SD SMD 95%−CI Weight
Tirzepatide 5 mg vs GLP-1 RAs
NCT03131687 52 −4.80 5.7105 53 −2.70 5.7318 −0.37 [−0.75; 0.02] 10.6%
SURPASS−2 113 −7.80 7.1542 470 −6.20 7.1466 −0.22 [−0.43; −0.02] 11.4%
SURPASS J−mono 220 −5.80 5.0438 160 −0.50 5.0438 −1.05 [−1.27; −0.83] 11.4%
Random effects model 385 683 −0.55 [−1.07; −0.04] 33.4%
Heterogeneity: I^2 = 93%, τ^2 = 0.1877, p < 0.01
Tirzepatide 10 mg vs GLP-1 RAs
NCT03131687 55 −8.70 5.7131 53 −2.70 5.7318 −1.05 [−1.45; −0.65] 10.5%
SURPASS−2 470 −10.30 7.3632 470 −6.20 7.1466 −0.57 [−0.70; −0.43] 11.7%
SURPASS J−mono 159 −8.50 5.0279 160 −0.50 5.0438 −1.59 [−1.84; −1.34] 11.3%
Random effects model 684 683 −1.06 [−1.66; −0.46] 33.4%
Heterogeneity: I^2 = 96%, τ^2 = 0.2612, p < 0.01
Tirzepatide 15 mg vs GLP-1 RAs
NCT03131687 51 −11.30 6.4065 53 −2.70 5.7318 −1.42 [−1.85; −0.99] 10.3%
SURPASS−2 469 −12.40 7.3710 470 −6.20 7.1466 −0.85 [−0.99; −0.72] 11.7%
SURPASS J−mono 158 −10.70 5.0596 160 −0.50 5.0438 −2.02 [−2.29; −1.75] 11.2%
Random effects model 678 683 −1.42 [−2.10; −0.74] 33.2%
Heterogeneity: I^2 = 97%, τ^2 = 0.3388, p < 0.01
Random effects model 1747 2049 −1.01 [−1.40; −0.62] 100.0%
Heterogeneity: I^2 = 95%, τ^2 = 0.3284, p < 0.01
Test for subgroup differences: χ^2_2 = 4.23, df = 2 (p = 0.12)
−2 −1 0 1 2

C.

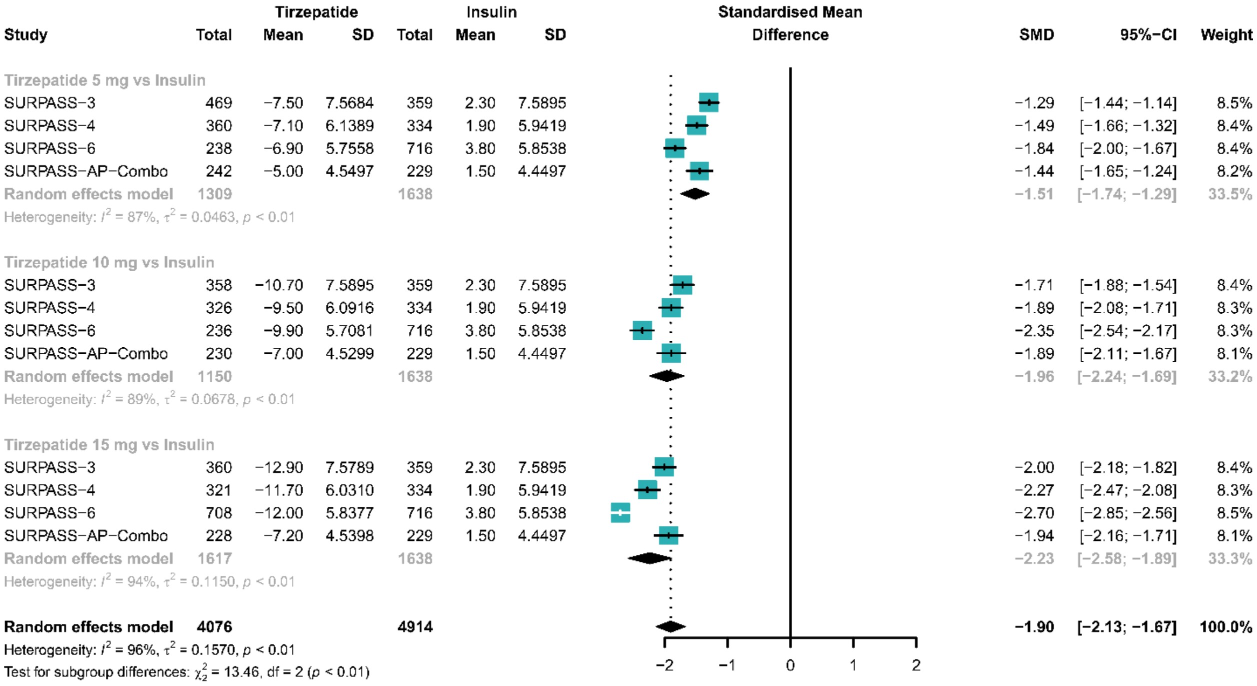
Tirzepatide
Insulin
Standardised Mean Difference
Study Total Mean SD Total Mean SD SMD 95%−CI Weight
Tirzepatide 5 mg vs Insulin
SURPASS−3 469 −7.50 7.5684 359 2.30 7.5895 −1.29 [−1.44; −1.14] 8.5%
SURPASS−4 360 −7.10 6.1389 334 1.90 5.9419 −1.49 [−1.66; −1.32] 8.4%
SURPASS−6 238 −6.90 5.7558 716 3.80 5.8538 −1.84 [−2.00; −1.67] 8.4%
SURPASS−AP−Combo 242 −5.00 4.5497 229 1.50 4.4497 −1.44 [−1.65; −1.24] 8.2%
Random effects model 1309 1638 −1.51 [−1.74; −1.29] 33.5%
Heterogeneity: I^2 = 87%, τ^2 = 0.0463, p < 0.01
Tirzepatide 10 mg vs Insulin
SURPASS−3 358 −10.70 7.5895 359 2.30 7.5895 −1.71 [−1.88; −1.54] 8.4%
SURPASS−4 326 −9.50 6.0916 334 1.90 5.9419 −1.89 [−2.08; −1.71] 8.3%
SURPASS−6 236 −9.90 5.7081 716 3.80 5.8538 −2.35 [−2.54; −2.17] 8.3%
SURPASS−AP−Combo 230 −7.00 4.5299 229 1.50 4.4497 −1.89 [−2.11; −1.67] 8.1%
Random effects model 1150 1638 −1.96 [−2.24; −1.69] 33.2%
Heterogeneity: I^2 = 89%, τ^2 = 0.0678, p < 0.01
Tirzepatide 15 mg vs Insulin
SURPASS−3 360 −12.90 7.5789 359 2.30 7.5895 −2.00 [−2.18; −1.82] 8.4%
SURPASS−4 321 −11.70 6.0310 334 1.90 5.9419 −2.27 [−2.47; −2.08] 8.3%
SURPASS−6 708 −12.00 5.8377 716 3.80 5.8538 −2.70 [−2.85; −2.56] 8.5%
SURPASS−AP−Combo 228 −7.20 4.5398 229 1.50 4.4497 −1.94 [−2.16; −1.71] 8.1%
Random effects model 1617 1638 −2.23 [−2.58; −1.89] 33.3%
Heterogeneity: I^2 = 94%, τ^2 = 0.1150, p < 0.01
Random effects model 4076 4914 −1.90 [−2.13; −1.67] 100.0%
Heterogeneity: I^2 = 96%, τ^2 = 0.1570, p < 0.01
Test for subgroup differences: χ^2_2 = 13.46, df = 2 (p < 0.01)
−2 −1 0 1 2

Supplementary Figure S5. Forest plot that demonstrates the change of waist circumference in different dose of tirzepatide when compared with placebo (A), glucagon like peptide-1 receptor agonist (GLP-1 RAs) (B).

A.

	Tirzepatide			Placebo					
**Study**	**Total**	**Mean**	**SD**	**Total**	**Mean**	**SD**	**SMD**	**95%-CI**	**Weight**
Tirzepatide 5 mg vs Placebo									
NCT03131687	55	−5.10	6.3800	51	−1.30	6.5000	−0.59	[−0.98; −0.20]	9.0%
SURMOUNT−1	630	−14.00	11.5300	643	−4.00	14.8800	−0.75	[−0.86; −0.64]	10.7%
Random effects model	685			694			−0.74	[−0.85; −0.63]	19.6%
Heterogeneity: $I^2 = 0\%$, $\tau^2 = 0$, $p = 0.44$									
Tirzepatide 10 mg vs Placebo									
NCT03131687	51	−7.40	6.2800	51	−1.30	6.5000	−0.95	[−1.36; −0.54]	8.8%
SURMOUNT−1	636	−17.70	12.2200	643	−4.00	14.8800	−1.01	[−1.12; −0.89]	10.7%
SURMOUNT−2	312	−10.80	10.6000	315	−3.30	8.8700	−0.77	[−0.93; −0.61]	10.5%
Random effects model	999			1009			−0.91	[−1.08; −0.73]	29.9%
Heterogeneity: $I^2 = 63\%$, $\tau^2 = 0.0146$, $p = 0.06$									
Tirzepatide 15 mg vs Placebo									
NCT03131687	53	−10.20	7.2800	51	−1.30	6.5000	−1.29	[−1.71; −0.86]	8.7%
SURMOUNT−1	630	−18.50	10.8900	643	−4.00	14.8800	−1.11	[−1.23; −0.99]	10.6%
SURMOUNT−2	311	−13.10	8.8200	315	−3.30	8.8700	−1.11	[−1.28; −0.94]	10.4%
SURMOUNT−3	287	−14.60	10.6000	292	0.20	10.6000	−1.40	[−1.58; −1.21]	10.4%
SURMOUNT−4	335	−22.80	6.3400	335	−9.10	6.3400	−2.16	[−2.35; −1.97]	10.3%
Random effects model	1616			1636			−1.41	[−1.81; −1.02]	50.5%
Heterogeneity: $I^2 = 96\%$, $\tau^2 = 0.1860$, $p < 0.01$									
**Random effects model**	**3300**			**3339**			**−1.12**	**[−1.40; −0.84]**	**100.0%**
Heterogeneity: $I^2 = 95\%$, $\tau^2 = 0.1856$, $p < 0.01$									
Test for subgroup differences: $\chi^2_2 = 11.87$, df = 2 ($p < 0.01$)									

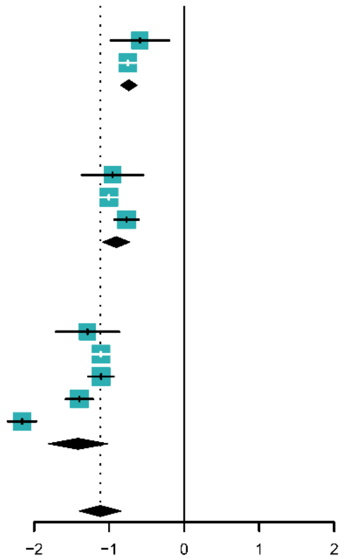

B.

	Tirzepatide			GLP-1 RAs					
**Study**	**Total**	**Mean**	**SD**	**Total**	**Mean**	**SD**	**SMD**	**95%-CI**	**Weight**
Tirzepatide 5 mg vs GLP-1 RAs									
SURPASS J−mono	159	−5.50	5.9300	159	0.30	5.9300	−0.98	[−1.21; −0.75]	17.9%
NCT03131687	55	−5.10	6.3800	54	−2.50	6.3900	−0.41	[−0.79; −0.03]	15.8%
Random effects model	214			213			−0.71	[−1.27; −0.16]	33.7%
Heterogeneity: $I^2 = 84\%$, $\tau^2 = 0.1372$, $p = 0.01$									
Tirzepatide 10 mg vs GLP-1 RAs									
SURPASS J−mono	158	−6.80	6.0300	159	0.30	5.9300	−1.19	[−1.43; −0.95]	17.9%
NCT03131687	51	−7.40	6.2800	54	−2.50	6.3900	−0.77	[−1.17; −0.38]	15.5%
Random effects model	209			213			−1.01	[−1.41; −0.61]	33.3%
Heterogeneity: $I^2 = 67\%$, $\tau^2 = 0.0578$, $p = 0.08$									
Tirzepatide 15 mg vs GLP-1 RAs									
SURPASS J−mono	160	−9.70	5.9500	159	0.30	5.9300	−1.68	[−1.94; −1.43]	17.6%
NCT03131687	53	−10.20	7.2800	54	−2.50	6.3900	−1.12	[−1.53; −0.72]	15.3%
Random effects model	213			213			−1.43	[−1.97; −0.88]	32.9%
Heterogeneity: $I^2 = 81\%$, $\tau^2 = 0.1258$, $p = 0.02$									
**Random effects model**	**636**			**639**			**−1.04**	**[−1.38; −0.70]**	**100.0%**
Heterogeneity: $I^2 = 86\%$, $\tau^2 = 0.1569$, $p < 0.01$									
Test for subgroup differences: $\chi^2_2 = 3.28$, df = 2 ($p = 0.19$)									

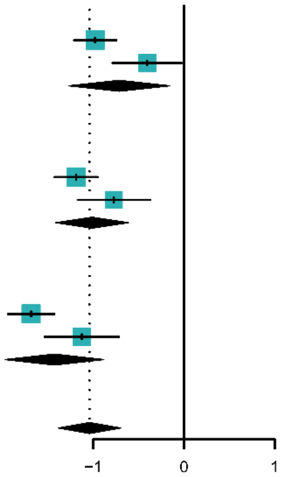

Supplementary Figure S6. Forest plot which demonstrates the change of HbA1c in different dose of tirzepatide when compared with placebo (A), glucagon like peptide-1 receptor agonist (GLP-1 RAs) (B), and insulin (C). A.

Study	Tirzepatide Total	Tirzepatide Mean	Tirzepatide SD	Placebo Total	Placebo Mean	Placebo SD	SMD	95%-CI	Weight
**Tirzepatide 5 mg vs Placebo**									
NCT03131687	55	−1.60	1.1100	51	0.10	1.1400	−1.51	[−1.94; −1.08]	5.2%
SURPASS−1	121	−1.87	0.9900	113	0.04	1.1700	−1.77	[−2.07; −1.46]	6.8%
SURPASS−5	116	−2.11	0.9300	120	−0.86	0.9200	−1.35	[−1.63; −1.07]	7.1%
SURMOUNT−1	630	−0.40	0.2600	643	−0.07	0.2600	−1.27	[−1.39; −1.15]	9.0%
Random effects model	922			927			−1.45	[−1.68; −1.21]	28.2%
Heterogeneity: $I^2 = 69\%$, $\tau^2 = 0.0359$, $p = 0.02$									
**Tirzepatide 10 mg vs Placebo**									
NCT03131687	51	−2.00	1.1400	51	0.10	1.1400	−1.84	[−2.31; −1.38]	4.9%
SURPASS−1	121	−1.89	1.1000	113	0.04	1.1700	−1.70	[−2.00; −1.40]	6.9%
SURPASS−5	119	−2.40	0.9200	120	−0.86	0.9200	−1.67	[−1.97; −1.38]	6.9%
SURMOUNT−1	636	−0.49	−6.3000	643	−0.07	0.2600			0.0%
SURMOUNT−2	312	−2.07	1.0600	315	−0.51	1.2400	−1.35	[−1.53; −1.18]	8.5%
Random effects model	1239			1242			−1.59	[−1.82; −1.37]	27.2%
Heterogeneity: $I^2 = 62\%$, $\tau^2 = 0.0302$, $p = 0.05$									
**Tirzepatide 15 mg vs Placebo**									
NCT03131687	53	−2.40	1.2400	51	0.10	1.1400	−2.10	[−2.58; −1.62]	4.7%
SURPASS−1	120	−2.07	1.1000	113	0.04	1.1700	−1.86	[−2.17; −1.55]	6.8%
SURPASS−5	120	−2.34	0.9800	120	−0.86	0.9200	−1.56	[−1.85; −1.27]	7.0%
SURMOUNT−1	630	−0.51	0.2600	643	−0.07	0.2600	−1.69	[−1.82; −1.56]	9.0%
SURMOUNT−2	311	−2.08	1.2300	315	−0.51	1.2400	−1.27	[−1.44; −1.10]	8.5%
SURMOUNT−3	287	−0.50	0.0000	292	0.00	0.0000			0.0%
SURMOUNT−4	335	−0.57	0.2800	335	−0.22	0.3700	−1.07	[−1.23; −0.90]	8.6%
Random effects model	1856			1869			−1.56	[−1.85; −1.27]	44.6%
Heterogeneity: $I^2 = 91\%$, $\tau^2 = 0.1143$, $p < 0.01$									
**Random effects model**	**4017**			**4038**			**−1.53**	**[−1.68; −1.39]**	**100.0%**

Heterogeneity: $I^2 = 83\%$, $\tau^2 = 0.0586$, $p < 0.01$
Test for subgroup differences: $\chi^2_2 = 0.84$, df = 2 ($p = 0.66$)

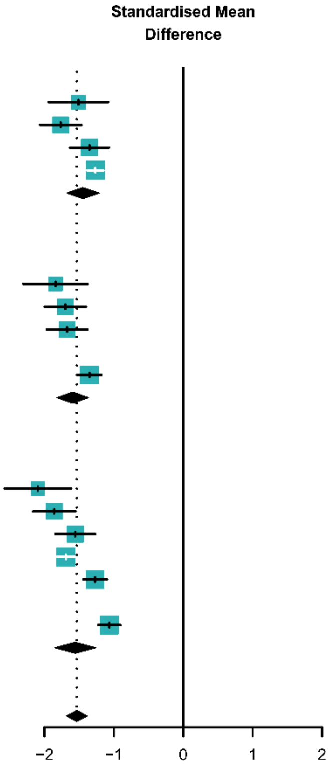

B.

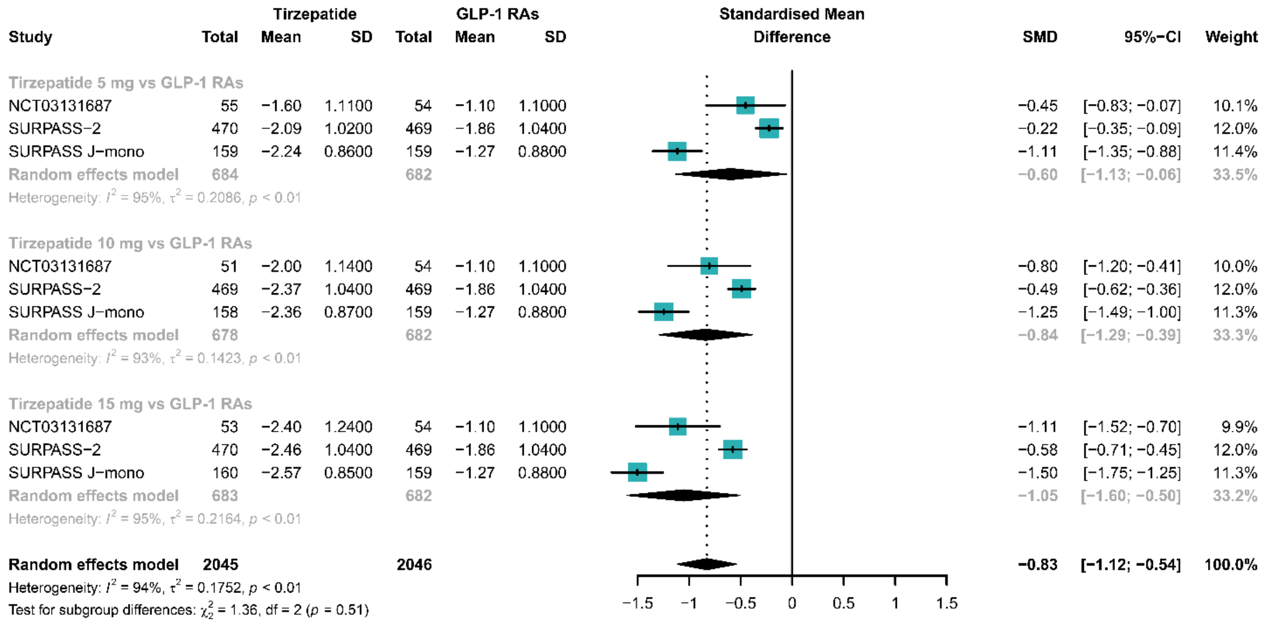

C.

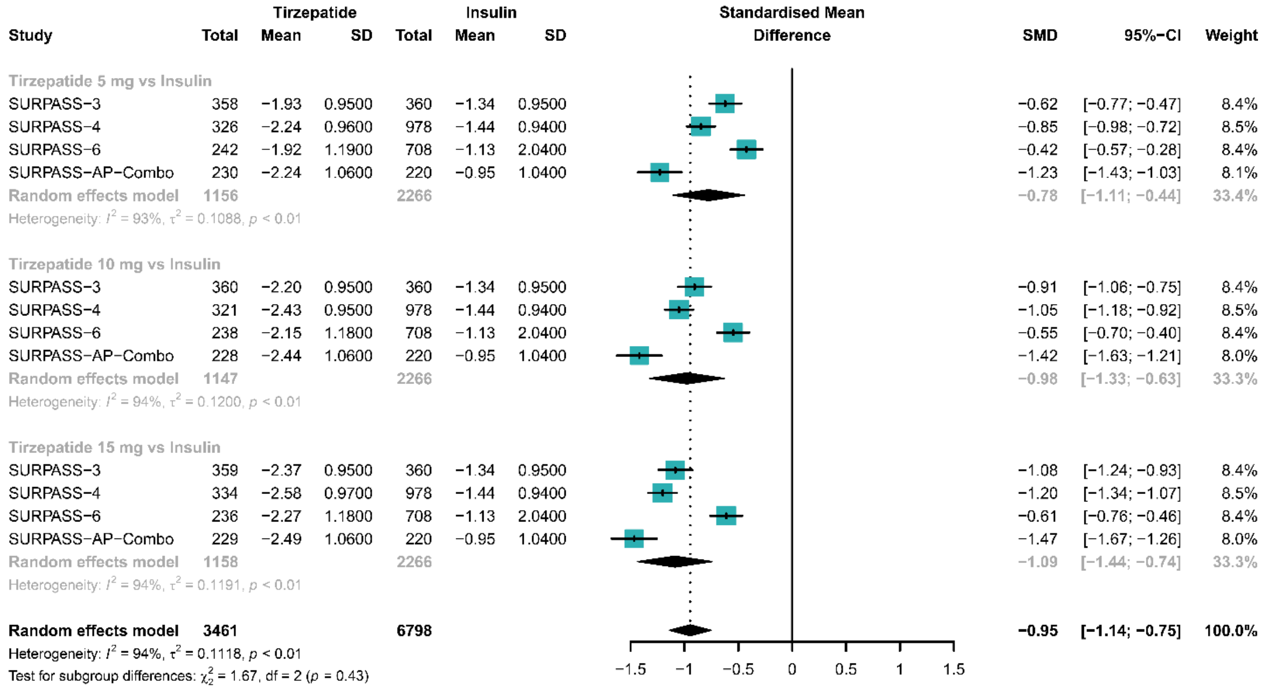

Supplementary Figure S7. Forest plot which demonstrates the change of DBP in different dose of tirzepatide when compared with placebo (A), glucagon like peptide-1 receptor agonist (GLP-1 RAs) (B), and insulin (C).

A.

Study	Tirzepatide Total	Tirzepatide Mean	Tirzepatide SD	Placebo Total	Placebo Mean	Placebo SD	SMD	95%-CI	Weight
**Tirzepatide 5 mg vs Placebo**									
NCT03131687	55	−0.70	8.7500	51	0.80	9.0000	−0.17	[−0.55; 0.21]	4.3%
SURPASS−1	121	−2.90	7.8100	113	−1.40	8.0800	−0.19	[−0.45; 0.07]	6.8%
SURPASS−5	116	−2.00	−7.8600	120	−2.10	7.7800			0.0%
SURMOUNT−1	630	−5.20	7.6800	643	−1.00	9.0600	−0.50	[−0.61; −0.39]	11.2%
Random effects model	922			927			−0.33	[−0.57; −0.09]	22.3%
Heterogeneity: I^2 = 70%, τ^2 = 0.0290, p = 0.04									
**Tirzepatide 10 mg vs Placebo**									
NCT03131687	51	−0.20	8.7100	51	0.80	9.0000	−0.11	[−0.50; 0.28]	4.2%
SURPASS−1	121	−3.10	7.9200	113	−1.40	8.0800	−0.21	[−0.47; 0.04]	6.8%
SURPASS−5	119	−3.30	7.8500	120	−2.10	7.7800	−0.15	[−0.41; 0.10]	6.9%
SURMOUNT−1	636	−5.50	7.7200	643	−1.00	9.0600	−0.53	[−0.65; −0.42]	11.2%
Random effects model	927			927			−0.29	[−0.51; −0.07]	29.1%
Heterogeneity: I^2 = 77%, τ^2 = 0.0339, p < 0.01									
**Tirzepatide 15 mg vs Placebo**									
NCT03131687	53	−0.70	9.2500	51	0.80	9.0000	−0.16	[−0.55; 0.22]	4.3%
SURPASS−1	120	−3.40	8.1100	113	−1.40	8.0800	−0.25	[−0.50; 0.01]	6.8%
SURPASS−5	120	−4.50	8.0000	120	−2.10	7.7800	−0.30	[−0.56; −0.05]	6.9%
SURMOUNT−1	630	−4.60	7.6800	643	−1.00	9.0600	−0.43	[−0.54; −0.32]	11.2%
SURMOUNT−3	287	−3.20	8.4700	292	2.30	8.5400	−0.65	[−0.81; −0.48]	9.5%
SURMOUNT−4	335	−5.50	8.4000	335	−1.70	8.4000	−0.45	[−0.61; −0.30]	9.9%
Random effects model	1545			1554			−0.42	[−0.54; −0.29]	48.5%
Heterogeneity: I^2 = 55%, τ^2 = 0.0130, p = 0.05									
**Random effects model**	**3394**			**3408**			**−0.36**	**[−0.46; −0.27]**	**100.0%**
Heterogeneity: I^2 = 61%, τ^2 = 0.0185, p < 0.01									
Test for subgroup differences: χ^2_2 = 1.15, df = 2 (p = 0.56)									

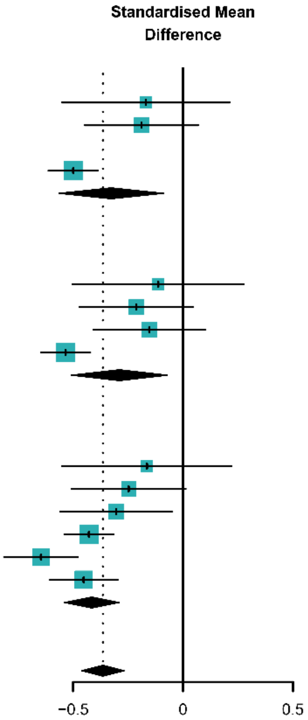

B.

Study	Tirzepatide Total	Tirzepatide Mean	Tirzepatide SD	GLP-1 RAs Total	GLP-1 RAs Mean	GLP-1 RAs SD	SMD	95%-CI	Weight
**Tirzepatide 5 mg vs GLP-1 RAs**									
NCT03131687	55	−0.70	8.7500	54	−1.40	8.8200	0.08	[−0.30; 0.46]	15.1%
SURPASS J−mono	159	−3.20	8.3200	159	0.10	8.3200	−0.40	[−0.62; −0.17]	18.3%
Random effects model	214			213			−0.18	[−0.65; 0.28]	33.4%
Heterogeneity: I^2 = 78%, τ^2 = 0.0887, p = 0.03									
**Tirzepatide 10 mg vs GLP-1 RAs**									
NCT03131687	51	−0.20	8.7100	54	−1.40	8.8200	0.14	[−0.25; 0.52]	15.0%
SURPASS J−mono	158	−4.00	8.3000	159	0.10	8.3200	−0.49	[−0.72; −0.27]	18.3%
Random effects model	209			213			−0.20	[−0.81; 0.42]	33.3%
Heterogeneity: I^2 = 87%, τ^2 = 0.1730, p < 0.01									
**Tirzepatide 15 mg vs GLP-1 RAs**									
NCT03131687	53	−0.70	9.2500	54	−1.40	8.8200	0.08	[−0.30; 0.46]	15.1%
SURPASS J−mono	160	−5.60	8.3500	159	0.10	8.3200	−0.68	[−0.91; −0.46]	18.2%
Random effects model	213			213			−0.32	[−1.06; 0.43]	33.3%
Heterogeneity: I^2 = 91%, τ^2 = 0.2645, p < 0.01									
**Random effects model**	**636**			**639**			**−0.24**	**[−0.53; 0.04]**	**100.0%**
Heterogeneity: I^2 = 81%, τ^2 = 0.1007, p < 0.01									
Test for subgroup differences: χ^2_2 = 0.10, df = 2 (p = 0.95)									

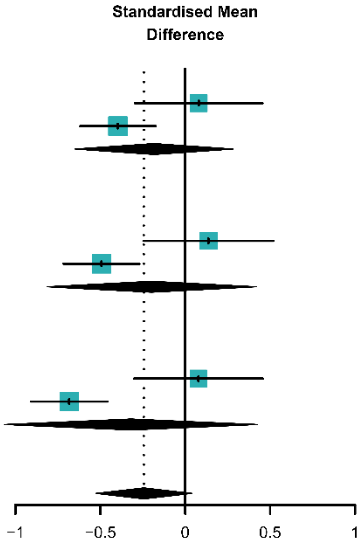

C.

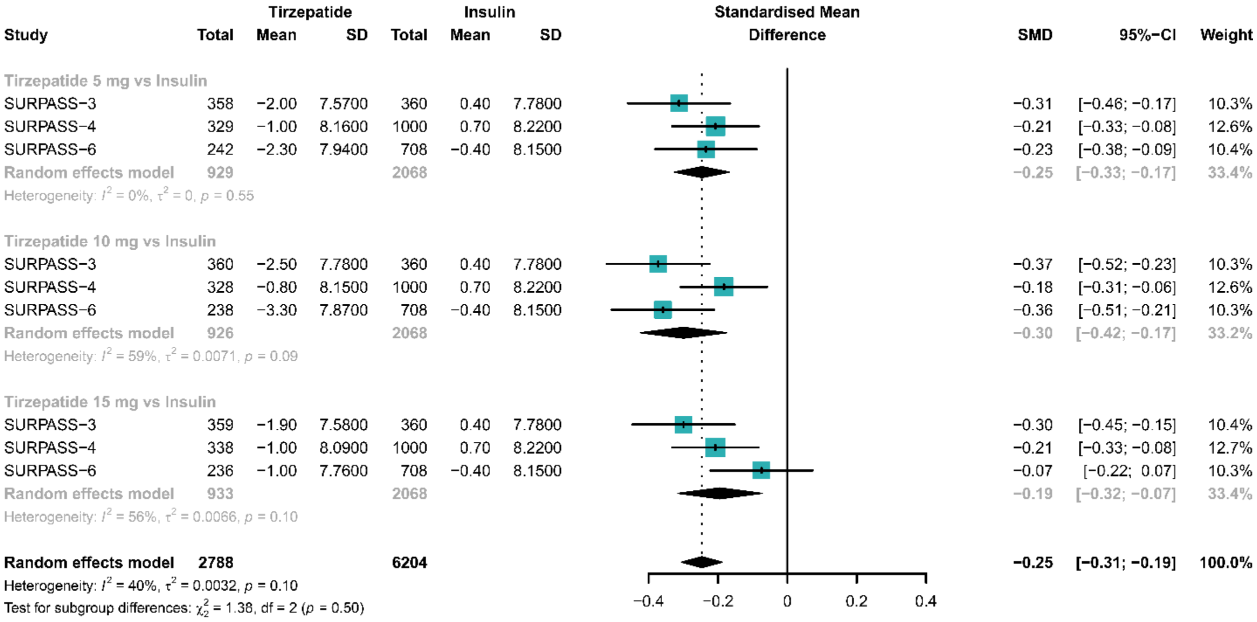

Supplementary Figure S8. Forest plot which demonstrates the change of SBP in different dose of tirzepatide when compared with placebo (A), glucagon like peptide-1 receptor agonist (GLP-1 RAs) (B), and insulin (C).

A.

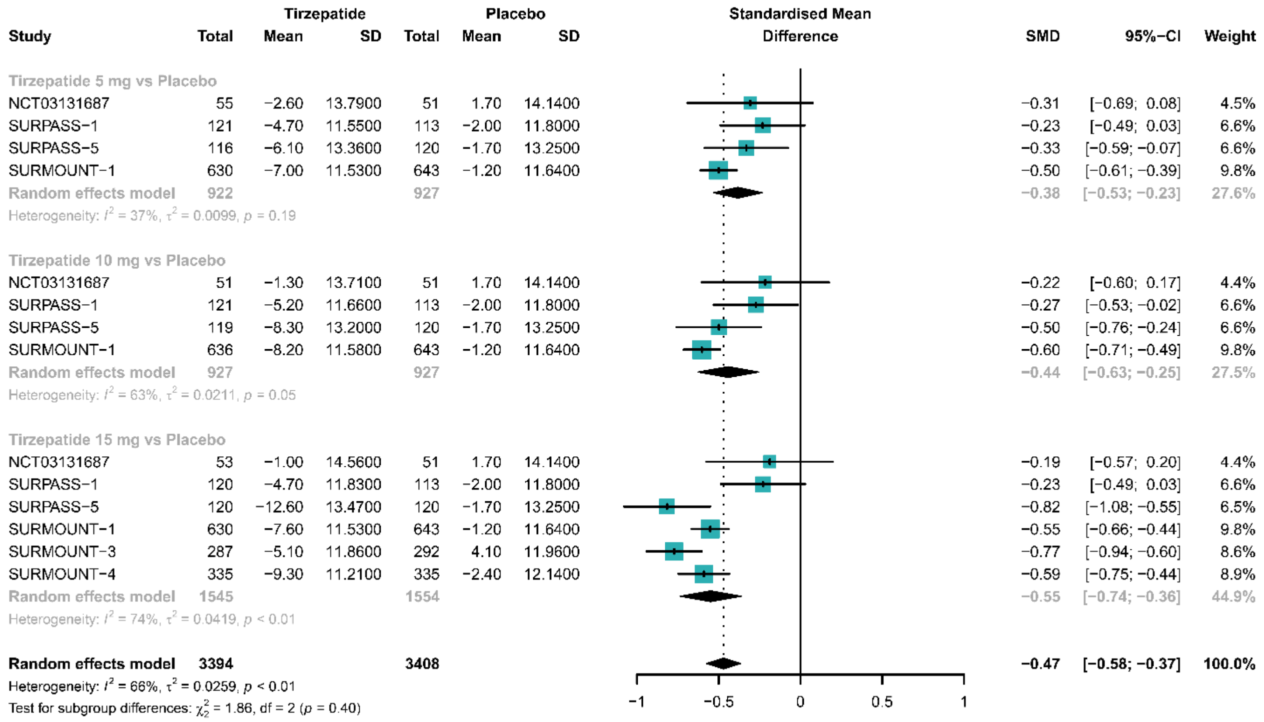

Tirzepatide
Placebo
Standardised Mean Difference
Study Total Mean SD Total Mean SD SMD 95%−CI Weight
Tirzepatide 5 mg vs Placebo
NCT03131687 55 −2.60 13.7900 51 1.70 14.1400 −0.31 [−0.69; 0.08] 4.5%
SURPASS−1 121 −4.70 11.5500 113 −2.00 11.8000 −0.23 [−0.49; 0.03] 6.6%
SURPASS−5 116 −6.10 13.3600 120 −1.70 13.2500 −0.33 [−0.59; −0.07] 6.6%
SURMOUNT−1 630 −7.00 11.5300 643 −1.20 11.6400 −0.50 [−0.61; −0.39] 9.8%
Random effects model 922 927 −0.38 [−0.53; −0.23] 27.6%
Heterogeneity: I^2 = 37%, τ^2 = 0.0099, p = 0.19
Tirzepatide 10 mg vs Placebo
NCT03131687 51 −1.30 13.7100 51 1.70 14.1400 −0.22 [−0.60; 0.17] 4.4%
SURPASS−1 121 −5.20 11.6600 113 −2.00 11.8000 −0.27 [−0.53; −0.02] 6.6%
SURPASS−5 119 −8.30 13.2000 120 −1.70 13.2500 −0.50 [−0.76; −0.24] 6.6%
SURMOUNT−1 636 −8.20 11.5800 643 −1.20 11.6400 −0.60 [−0.71; −0.49] 9.8%
Random effects model 927 927 −0.44 [−0.63; −0.25] 27.5%
Heterogeneity: I^2 = 63%, τ^2 = 0.0211, p = 0.05
Tirzepatide 15 mg vs Placebo
NCT03131687 53 −1.00 14.5600 51 1.70 14.1400 −0.19 [−0.57; 0.20] 4.4%
SURPASS−1 120 −4.70 11.8300 113 −2.00 11.8000 −0.23 [−0.49; 0.03] 6.6%
SURPASS−5 120 −12.60 13.4700 120 −1.70 13.2500 −0.82 [−1.08; −0.55] 6.5%
SURMOUNT−1 630 −7.60 11.5300 643 −1.20 11.6400 −0.55 [−0.66; −0.44] 9.8%
SURMOUNT−3 287 −5.10 11.8600 292 4.10 11.9600 −0.77 [−0.94; −0.60] 8.6%
SURMOUNT−4 335 −9.30 11.2100 335 −2.40 12.1400 −0.59 [−0.75; −0.44] 8.9%
Random effects model 1545 1554 −0.55 [−0.74; −0.36] 44.9%
Heterogeneity: I^2 = 74%, τ^2 = 0.0419, p < 0.01
Random effects model 3394 3408 −0.47 [−0.58; −0.37] 100.0%
Heterogeneity: I^2 = 66%, τ^2 = 0.0259, p < 0.01
Test for subgroup differences: χ^2_2 = 1.86, df = 2 (p = 0.40)
−1 −0.5 0 0.5 1

B.

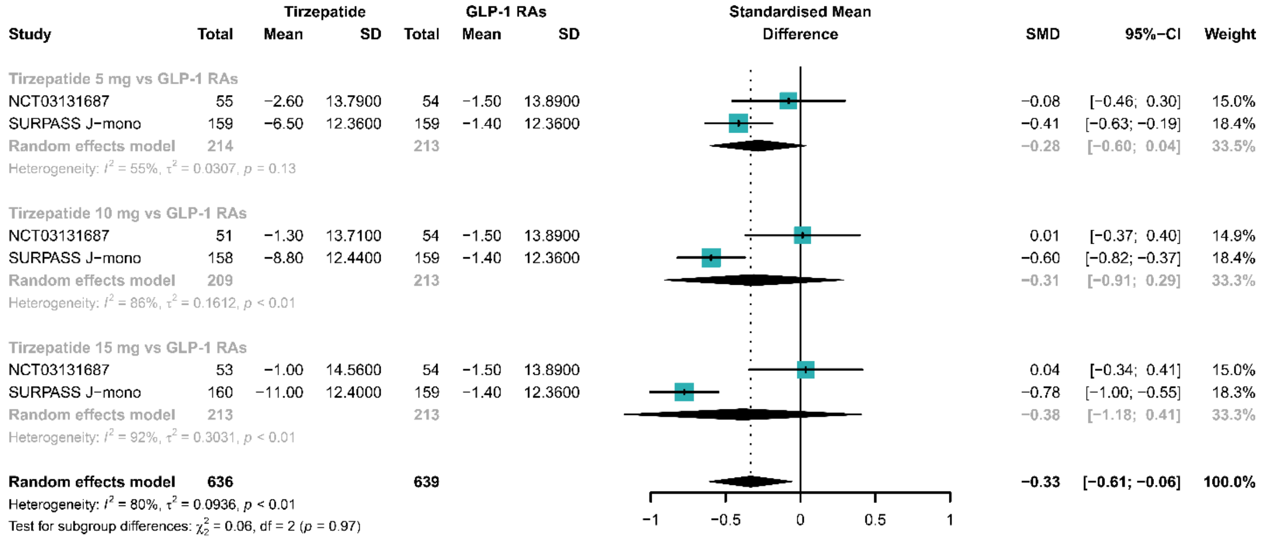

Tirzepatide
GLP-1 RAs
Standardised Mean Difference
Study Total Mean SD Total Mean SD SMD 95%−CI Weight
Tirzepatide 5 mg vs GLP-1 RAs
NCT03131687 55 −2.60 13.7900 54 −1.50 13.8900 −0.08 [−0.46; 0.30] 15.0%
SURPASS J−mono 159 −6.50 12.3600 159 −1.40 12.3600 −0.41 [−0.63; −0.19] 18.4%
Random effects model 214 213 −0.28 [−0.60; 0.04] 33.5%
Heterogeneity: I^2 = 55%, τ^2 = 0.0307, p = 0.13
Tirzepatide 10 mg vs GLP-1 RAs
NCT03131687 51 −1.30 13.7100 54 −1.50 13.8900 0.01 [−0.37; 0.40] 14.9%
SURPASS J−mono 158 −8.80 12.4400 159 −1.40 12.3600 −0.60 [−0.82; −0.37] 18.4%
Random effects model 209 213 −0.31 [−0.91; 0.29] 33.3%
Heterogeneity: I^2 = 86%, τ^2 = 0.1612, p < 0.01
Tirzepatide 15 mg vs GLP-1 RAs
NCT03131687 53 −1.00 14.5600 54 −1.50 13.8900 0.04 [−0.34; 0.41] 15.0%
SURPASS J−mono 160 −11.00 12.4000 159 −1.40 12.3600 −0.78 [−1.00; −0.55] 18.3%
Random effects model 213 213 −0.38 [−1.18; 0.41] 33.3%
Heterogeneity: I^2 = 92%, τ^2 = 0.3031, p < 0.01
Random effects model 636 639 −0.33 [−0.61; −0.06] 100.0%
Heterogeneity: I^2 = 80%, τ^2 = 0.0936, p < 0.01
Test for subgroup differences: χ^2_2 = 0.06, df = 2 (p = 0.97)
−1 −0.5 0 0.5 1

C.

Study	Tirzepatide Total	Tirzepatide Mean	Tirzepatide SD	Insulin Total	Insulin Mean	Insulin SD	SMD	95%−CI	Weight
**Tirzepatide 5 mg vs Insulin**									
SURPASS−3	358	−4.90	12.3000	360	0.50	12.3300	−0.44	[−0.59; −0.29]	10.7%
SURPASS−4	329	−2.80	13.9700	1000	1.30	13.9100	−0.29	[−0.42; −0.17]	12.0%
SURPASS−6	242	−7.40	11.9100	708	−0.40	12.2200	−0.58	[−0.72; −0.43]	10.7%
**Random effects model**	**929**			**2068**			**−0.43**	**[−0.59; −0.27]**	**33.4%**
Heterogeneity: $I^2 = 76\%$, $\tau^2 = 0.0153$, $p = 0.02$									
**Tirzepatide 10 mg vs Insulin**									
SURPASS−3	360	−6.60	12.5200	360	0.50	12.3300	−0.57	[−0.72; −0.42]	10.7%
SURPASS−4	328	−3.70	13.7600	1000	1.30	13.9100	−0.36	[−0.49; −0.23]	12.0%
SURPASS−6	238	−9.00	12.5900	708	−0.40	12.2200	−0.70	[−0.85; −0.55]	10.6%
**Random effects model**	**926**			**2068**			**−0.54**	**[−0.74; −0.34]**	**33.2%**
Heterogeneity: $I^2 = 83\%$, $\tau^2 = 0.0247$, $p < 0.01$									
**Tirzepatide 15 mg vs Insulin**									
SURPASS−3	359	−5.50	12.3200	360	0.50	12.3300	−0.49	[−0.64; −0.34]	10.7%
SURPASS−4	338	−4.80	13.6000	1000	1.30	13.9100	−0.44	[−0.57; −0.32]	12.0%
SURPASS−6	236	−5.90	12.5400	708	−0.40	12.2200	−0.45	[−0.60; −0.30]	10.7%
**Random effects model**	**933**			**2068**			**−0.46**	**[−0.54; −0.38]**	**33.4%**
Heterogeneity: $I^2 = 0\%$, $\tau^2 = 0$, $p = 0.89$									
**Random effects model**	**2788**			**6204**			**−0.47**	**[−0.55; −0.39]**	**100.0%**
Heterogeneity: $I^2 = 66\%$, $\tau^2 = 0.0099$, $p < 0.01$									
Test for subgroup differences: $\chi^2_2 = 0.75$, df = 2 ($p = 0.69$)									

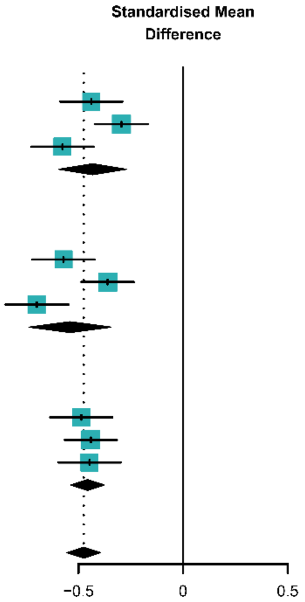

Supplementary Figure S9. Forest plot which demonstrates the proportion of patients with any adverse events in different dose of tirzepatide when compared with placebo (A), glucagon like peptide-1 receptor agonist (GLP-1 RAs) (B), and insulin (C).

A.

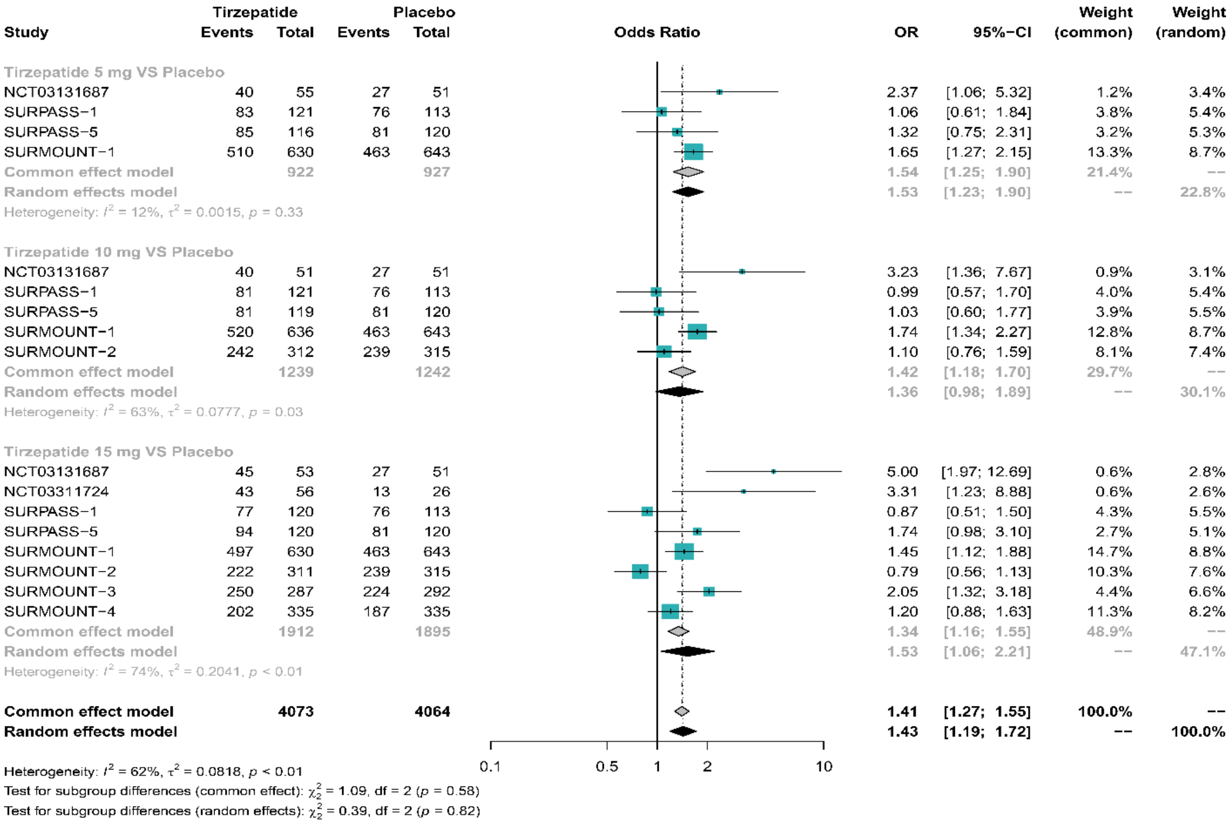

B.

Study	Tirzepatide Events	Tirzepatide Total	GLP-1 RAs Events	GLP-1 RAs Total	OR	95%−CI	Weight (common)	Weight (random)
**Tirzepatide 5 mg VS GLP-1 RAs**								
NCT03131687	40	55	40	54	0.93	[0.40; 2.18]	2.8%	2.5%
SURPASS−2	299	470	301	469	0.98	[0.75; 1.27]	27.9%	25.6%
SURPASS J−mono	131	159	123	159	1.37	[0.79; 2.38]	5.5%	6.0%
Common effect model		684		682	1.03	[0.82; 1.30]	36.2%	−−
Random effects model					1.03	[0.82; 1.30]	−−	34.1%
Heterogeneity: $I^2 = 0\%$, $\tau^2 = 0$, $p = 0.54$								
**Tirzepatide 10 mg VS GLP-1 RAs**								
NCT03131687	40	51	40	54	1.27	[0.52; 3.14]	2.1%	2.2%
SURPASS−2	322	469	301	469	1.22	[0.93; 1.60]	24.0%	24.7%
SURPASS J−mono	121	158	123	159	0.96	[0.57; 1.61]	7.3%	6.6%
Common effect model		678		682	1.17	[0.93; 1.47]	33.4%	−−
Random effects model					1.17	[0.93; 1.47]	−−	33.5%
Heterogeneity: $I^2 = 0\%$, $\tau^2 = 0$, $p = 0.70$								
**Tirzepatide 15 mg VS GLP-1 RAs**								
NCT03131687	45	53	40	54	1.97	[0.75; 5.18]	1.5%	1.9%
SURPASS−2	324	470	301	469	1.24	[0.94; 1.63]	23.8%	24.6%
SURPASS J−mono	134	160	123	159	1.51	[0.86; 2.64]	5.1%	5.8%
Common effect model		683		682	1.32	[1.04; 1.67]	30.4%	−−
Random effects model					1.32	[1.04; 1.67]	−−	32.4%
Heterogeneity: $I^2 = 0\%$, $\tau^2 = 0$, $p = 0.58$								
**Common effect model**		**2045**		**2046**	**1.17**	**[1.02; 1.33]**	**100.0%**	**−−**
**Random effects model**					**1.16**	**[1.02; 1.33]**	**−−**	**100.0%**

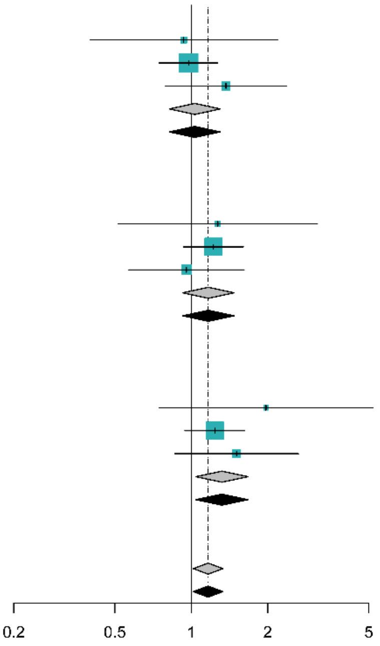

Heterogeneity: $I^2 = 0\%$, $\tau^2 = 0$, $p = 0.74$
Test for subgroup differences (common effect): $\chi^2_2 = 2.13$, df = 2 ($p = 0.34$)
Test for subgroup differences (random effects): $\chi^2_2 = 2.11$, df = 2 ($p = 0.35$)

C.

Study	Tirzepatide Events	Tirzepatide Total	Insulin Events	Insulin Total	OR	95%−CI	Weight (common)	Weight (random)
**Tirzepatide 5 mg VS Insulin**								
SURPASS−3	219	358	193	360	1.36	[1.01; 1.83]	11.3%	8.8%
SURPASS−4	232	329	679	1000	1.13	[0.86; 1.48]	14.9%	9.0%
SURPASS−6	170	243	394	708	1.86	[1.36; 2.53]	9.1%	8.7%
SURPASS−AP−Combo	200	230	157	220	2.68	[1.65; 4.33]	3.2%	7.6%
Common effect model		1160		2288	1.50	[1.28; 1.75]	38.5%	−−
Random effects model					1.61	[1.14; 2.27]	−−	34.1%
Heterogeneity: $I^2 = 75\%$, $\tau^2 = 0.0933$, $p < 0.01$								
**Tirzepatide 10 mg VS Insulin**								
SURPASS−3	248	360	193	360	1.92	[1.41; 2.60]	9.1%	8.8%
SURPASS−4	241	328	679	1000	1.31	[0.99; 1.73]	13.4%	8.9%
SURPASS−6	168	238	394	708	1.91	[1.39; 2.62]	8.8%	8.7%
SURPASS−AP−Combo	216	228	157	220	7.22	[3.77; 13.84]	1.3%	6.3%
Common effect model		1154		2288	1.87	[1.59; 2.21]	32.5%	−−
Random effects model					2.32	[1.18; 4.56]	−−	32.8%
Heterogeneity: $I^2 = 87\%$, $\tau^2 = 0.4343$, $p < 0.01$								
**Tirzepatide 15 mg VS Insulin**								
SURPASS−3	263	359	193	360	2.37	[1.73; 3.24]	7.8%	8.7%
SURPASS−4	259	338	679	1000	1.55	[1.17; 2.06]	12.1%	8.9%
SURPASS−6	177	236	394	708	2.39	[1.72; 3.33]	7.4%	8.6%
SURPASS−AP−Combo	213	229	157	220	5.34	[2.97; 9.60]	1.7%	6.8%
Common effect model		1162		2288	2.21	[1.86; 2.61]	29.0%	−−
Random effects model					2.50	[1.58; 3.94]	−−	33.1%
Heterogeneity: $I^2 = 80\%$, $\tau^2 = 0.1797$, $p < 0.01$								
**Common effect model**		**3476**		**6864**	**1.82**	**[1.66; 2.01]**	**100.0%**	**−−**
**Random effects model**					**2.08**	**[1.58; 2.74]**	**−−**	**100.0%**

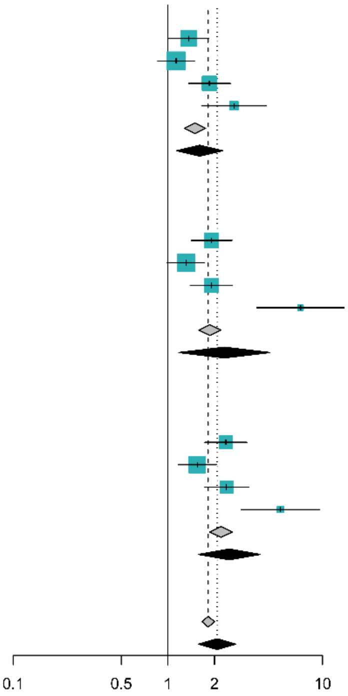

Heterogeneity: $I^2 = 82\%$, $\tau^2 = 0.1996$, $p < 0.01$
Test for subgroup differences (common effect): $\chi^2_2 = 10.90$, df = 2 ($p < 0.01$)
Test for subgroup differences (random effects): $\chi^2_2 = 2.59$, df = 2 ($p = 0.27$)

Supplementary Figure S10. Forest plot which demonstrates the proportion of patients with severe adverse events in different dose of tirzepatide when compared with placebo (A), glucagon like peptide-1 receptor agonist (GLP-1 RAs) (B), and insulin (C).

A.

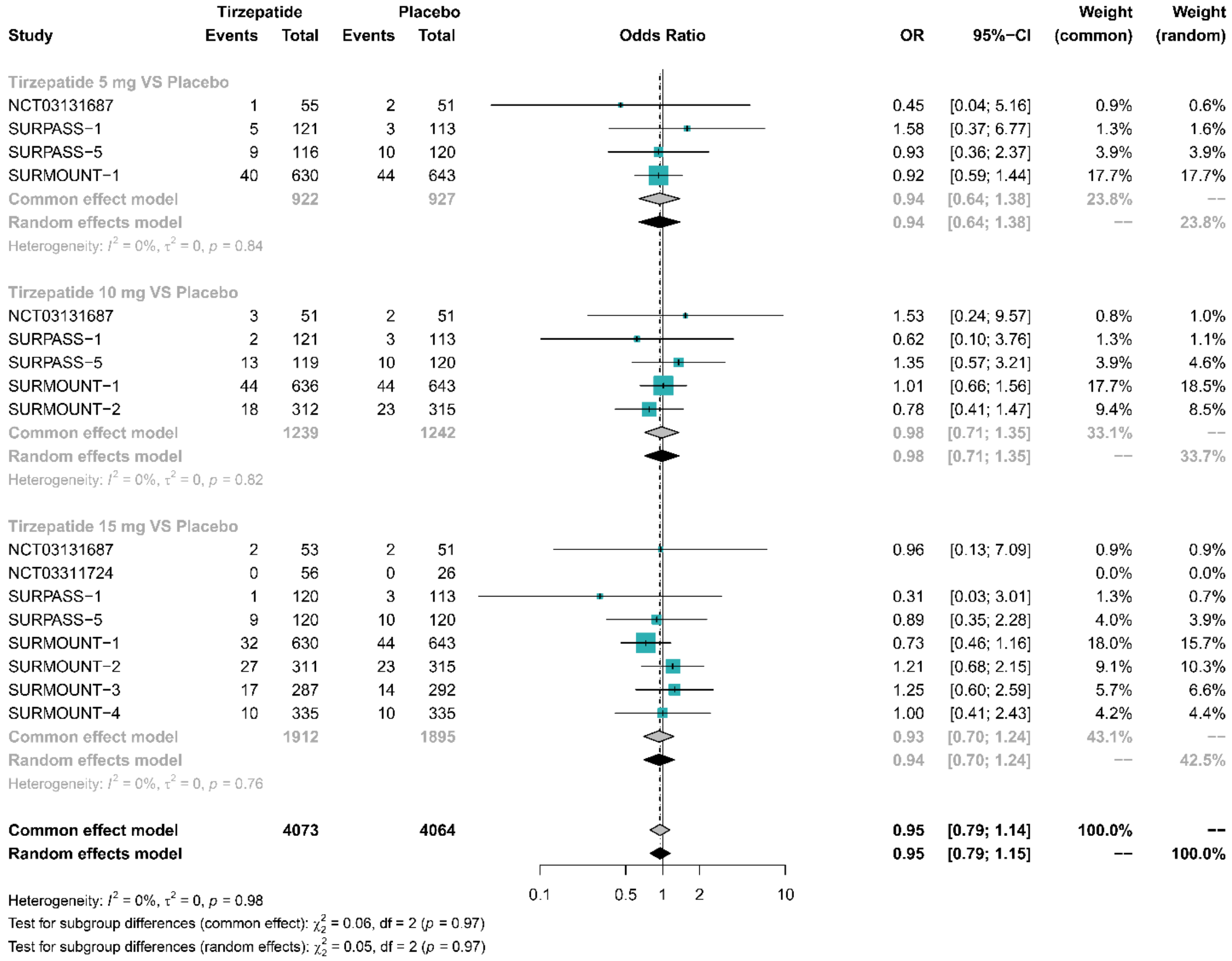
Tirzepatide
Placebo
Study Events Total Events Total
Odds Ratio
OR 95%−CI
Weight (common)
Weight (random)
Tirzepatide 5 mg VS Placebo
NCT03131687 1 55 2 51 0.45 [0.04; 5.16] 0.9% 0.6%
SURPASS−1 5 121 3 113 1.58 [0.37; 6.77] 1.3% 1.6%
SURPASS−5 9 116 10 120 0.93 [0.36; 2.37] 3.9% 3.9%
SURMOUNT−1 40 630 44 643 0.92 [0.59; 1.44] 17.7% 17.7%
Common effect model 922 927 0.94 [0.64; 1.38] 23.8% --
Random effects model 0.94 [0.64; 1.38] -- 23.8%
Heterogeneity: I^2 = 0%, τ^2 = 0, p = 0.84
Tirzepatide 10 mg VS Placebo
NCT03131687 3 51 2 51 1.53 [0.24; 9.57] 0.8% 1.0%
SURPASS−1 2 121 3 113 0.62 [0.10; 3.76] 1.3% 1.1%
SURPASS−5 13 119 10 120 1.35 [0.57; 3.21] 3.9% 4.6%
SURMOUNT−1 44 636 44 643 1.01 [0.66; 1.56] 17.7% 18.5%
SURMOUNT−2 18 312 23 315 0.78 [0.41; 1.47] 9.4% 8.5%
Common effect model 1239 1242 0.98 [0.71; 1.35] 33.1% --
Random effects model 0.98 [0.71; 1.35] -- 33.7%
Heterogeneity: I^2 = 0%, τ^2 = 0, p = 0.82
Tirzepatide 15 mg VS Placebo
NCT03131687 2 53 2 51 0.96 [0.13; 7.09] 0.9% 0.9%
NCT03311724 0 56 0 26 0.0% 0.0%
SURPASS−1 1 120 3 113 0.31 [0.03; 3.01] 1.3% 0.7%
SURPASS−5 9 120 10 120 0.89 [0.35; 2.28] 4.0% 3.9%
SURMOUNT−1 32 630 44 643 0.73 [0.46; 1.16] 18.0% 15.7%
SURMOUNT−2 27 311 23 315 1.21 [0.68; 2.15] 9.1% 10.3%
SURMOUNT−3 17 287 14 292 1.25 [0.60; 2.59] 5.7% 6.6%
SURMOUNT−4 10 335 10 335 1.00 [0.41; 2.43] 4.2% 4.4%
Common effect model 1912 1895 0.93 [0.70; 1.24] 43.1% --
Random effects model 0.94 [0.70; 1.24] -- 42.5%
Heterogeneity: I^2 = 0%, τ^2 = 0, p = 0.76
Common effect model 4073 4064 0.95 [0.79; 1.14] 100.0% --
Random effects model 0.95 [0.79; 1.15] -- 100.0%
0.1 0.5 1 2 10
Heterogeneity: I^2 = 0%, τ^2 = 0, p = 0.98
Test for subgroup differences (common effect): χ^2_2 = 0.06, df = 2 (p = 0.97)
Test for subgroup differences (random effects): χ^2_2 = 0.05, df = 2 (p = 0.97)

B.

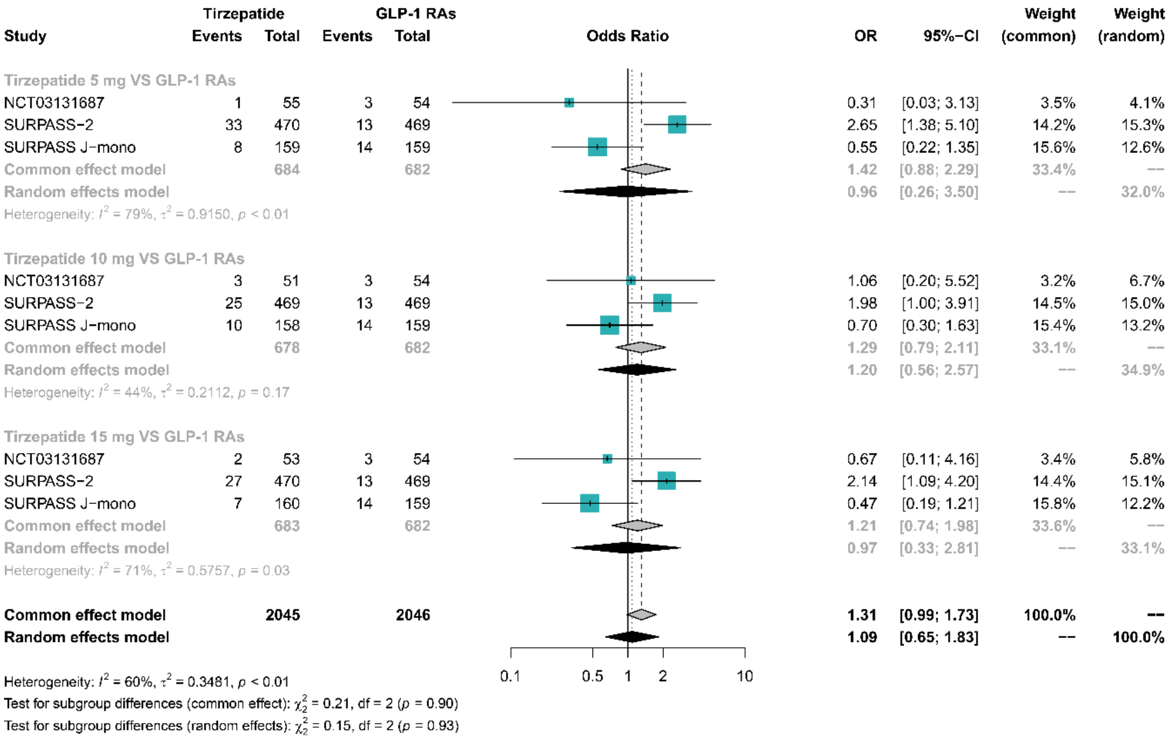

C.

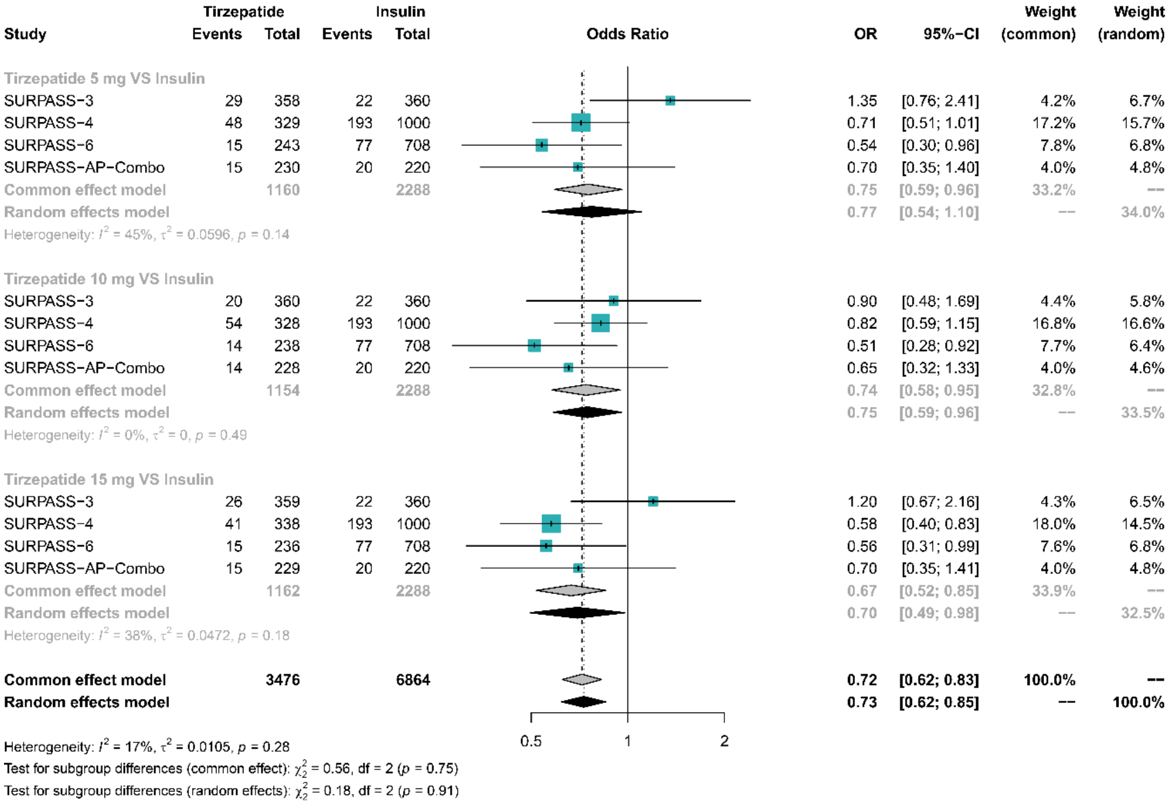

Supplementary Figure S11. Forest plot which demonstrates the proportion of patients with discontinuation of treatment due to adverse events in different dose of tirzepatide when compared with placebo (A), glucagon like peptide-1 receptor agonist (GLP-1 RAs) (B), and insulin (C).

A.

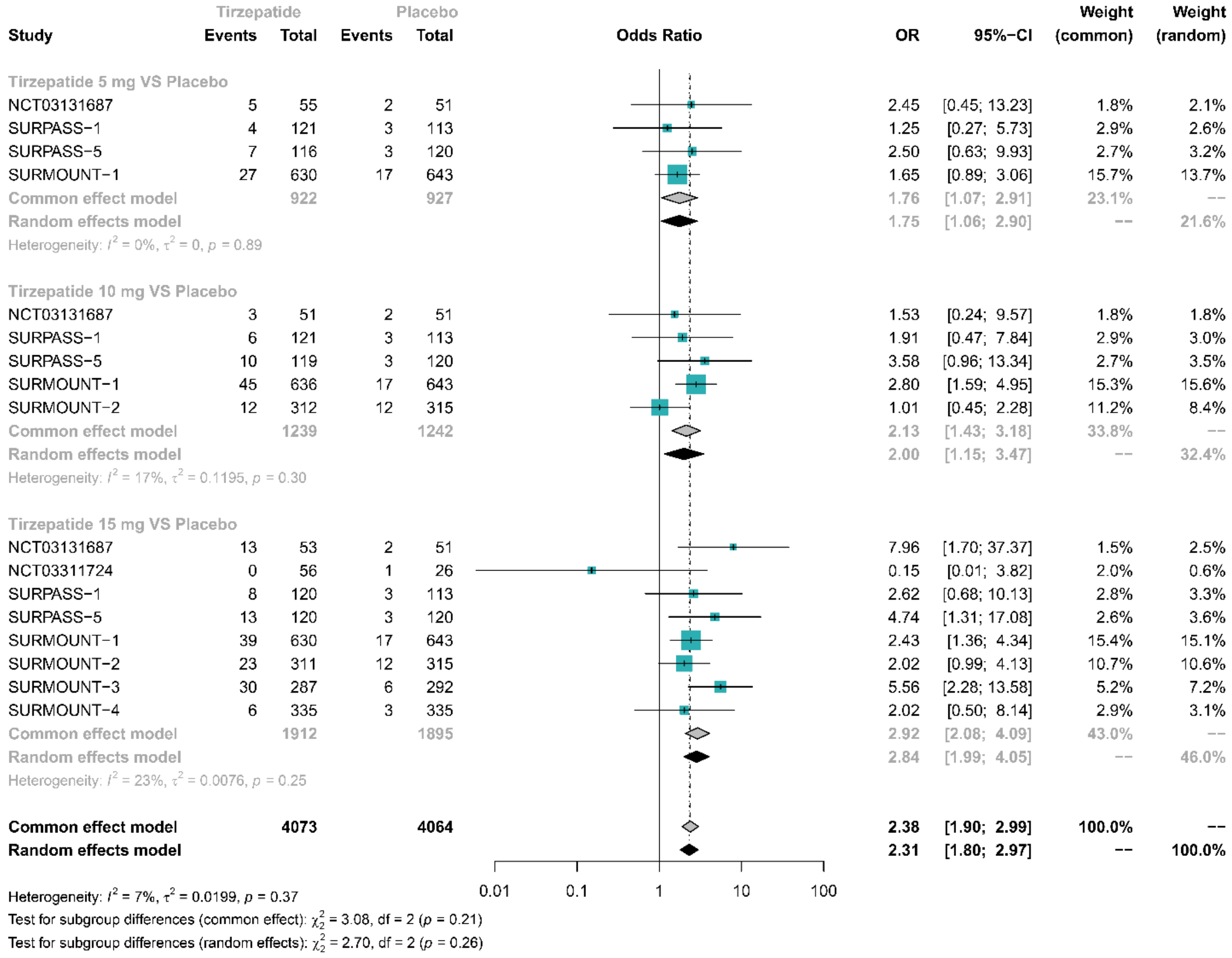
Tirzepatide
Placebo
Study Events Total Events Total
Odds Ratio
OR 95%−CI
Weight (common)
Weight (random)
Tirzepatide 5 mg VS Placebo
NCT03131687 5 55 2 51 2.45 [0.45; 13.23] 1.8% 2.1%
SURPASS−1 4 121 3 113 1.25 [0.27; 5.73] 2.9% 2.6%
SURPASS−5 7 116 3 120 2.50 [0.63; 9.93] 2.7% 3.2%
SURMOUNT−1 27 630 17 643 1.65 [0.89; 3.06] 15.7% 13.7%
Common effect model 922 927 1.76 [1.07; 2.91] 23.1% --
Random effects model 1.75 [1.06; 2.90] -- 21.6%
Heterogeneity: I^2 = 0%, τ^2 = 0, p = 0.89
Tirzepatide 10 mg VS Placebo
NCT03131687 3 51 2 51 1.53 [0.24; 9.57] 1.8% 1.8%
SURPASS−1 6 121 3 113 1.91 [0.47; 7.84] 2.9% 3.0%
SURPASS−5 10 119 3 120 3.58 [0.96; 13.34] 2.7% 3.5%
SURMOUNT−1 45 636 17 643 2.80 [1.59; 4.95] 15.3% 15.6%
SURMOUNT−2 12 312 12 315 1.01 [0.45; 2.28] 11.2% 8.4%
Common effect model 1239 1242 2.13 [1.43; 3.18] 33.8% --
Random effects model 2.00 [1.15; 3.47] -- 32.4%
Heterogeneity: I^2 = 17%, τ^2 = 0.1195, p = 0.30
Tirzepatide 15 mg VS Placebo
NCT03131687 13 53 2 51 7.96 [1.70; 37.37] 1.5% 2.5%
NCT03311724 0 56 1 26 0.15 [0.01; 3.82] 2.0% 0.6%
SURPASS−1 8 120 3 113 2.62 [0.68; 10.13] 2.8% 3.3%
SURPASS−5 13 120 3 120 4.74 [1.31; 17.08] 2.6% 3.6%
SURMOUNT−1 39 630 17 643 2.43 [1.36; 4.34] 15.4% 15.1%
SURMOUNT−2 23 311 12 315 2.02 [0.99; 4.13] 10.7% 10.6%
SURMOUNT−3 30 287 6 292 5.56 [2.28; 13.58] 5.2% 7.2%
SURMOUNT−4 6 335 3 335 2.02 [0.50; 8.14] 2.9% 3.1%
Common effect model 1912 1895 2.92 [2.08; 4.09] 43.0% --
Random effects model 2.84 [1.99; 4.05] -- 46.0%
Heterogeneity: I^2 = 23%, τ^2 = 0.0076, p = 0.25
Common effect model 4073 4064 2.38 [1.90; 2.99] 100.0% --
Random effects model 2.31 [1.80; 2.97] -- 100.0%
0.01 0.1 1 10 100
Heterogeneity: I^2 = 7%, τ^2 = 0.0199, p = 0.37
Test for subgroup differences (common effect): χ^2_2 = 3.08, df = 2 (p = 0.21)
Test for subgroup differences (random effects): χ^2_2 = 2.70, df = 2 (p = 0.26)

B.

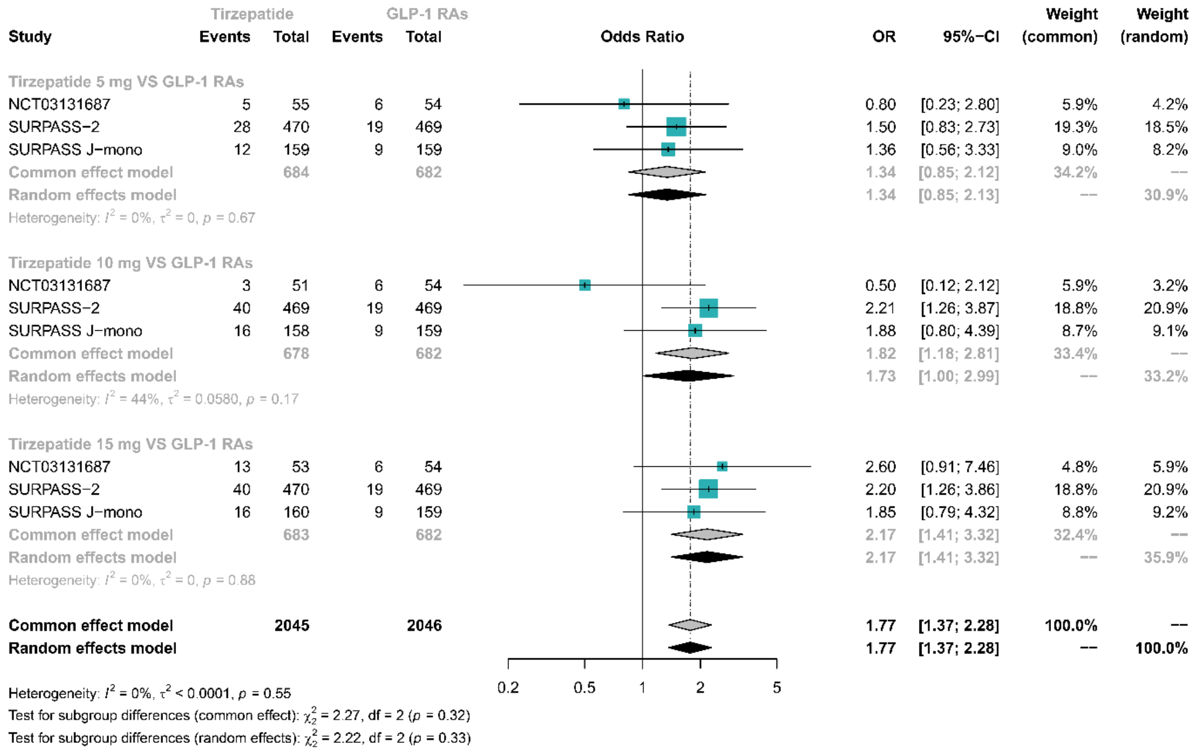

C.

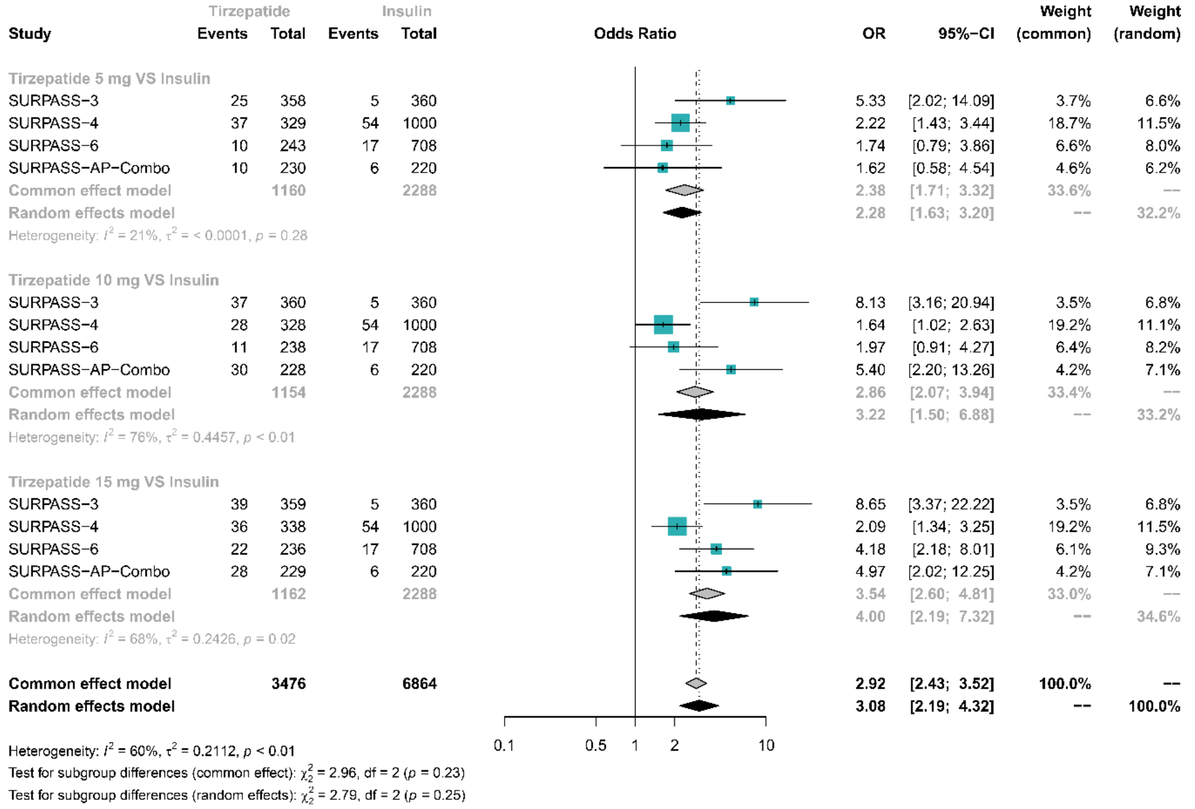

Supplementary Figure S12. Forest plot which demonstrates the proportion of patients with nausea in different dose of tirzepatide when compared with placebo (A), glucagon like peptide-1 receptor agonist (GLP-1 RAs) (B), and insulin (C).

A.

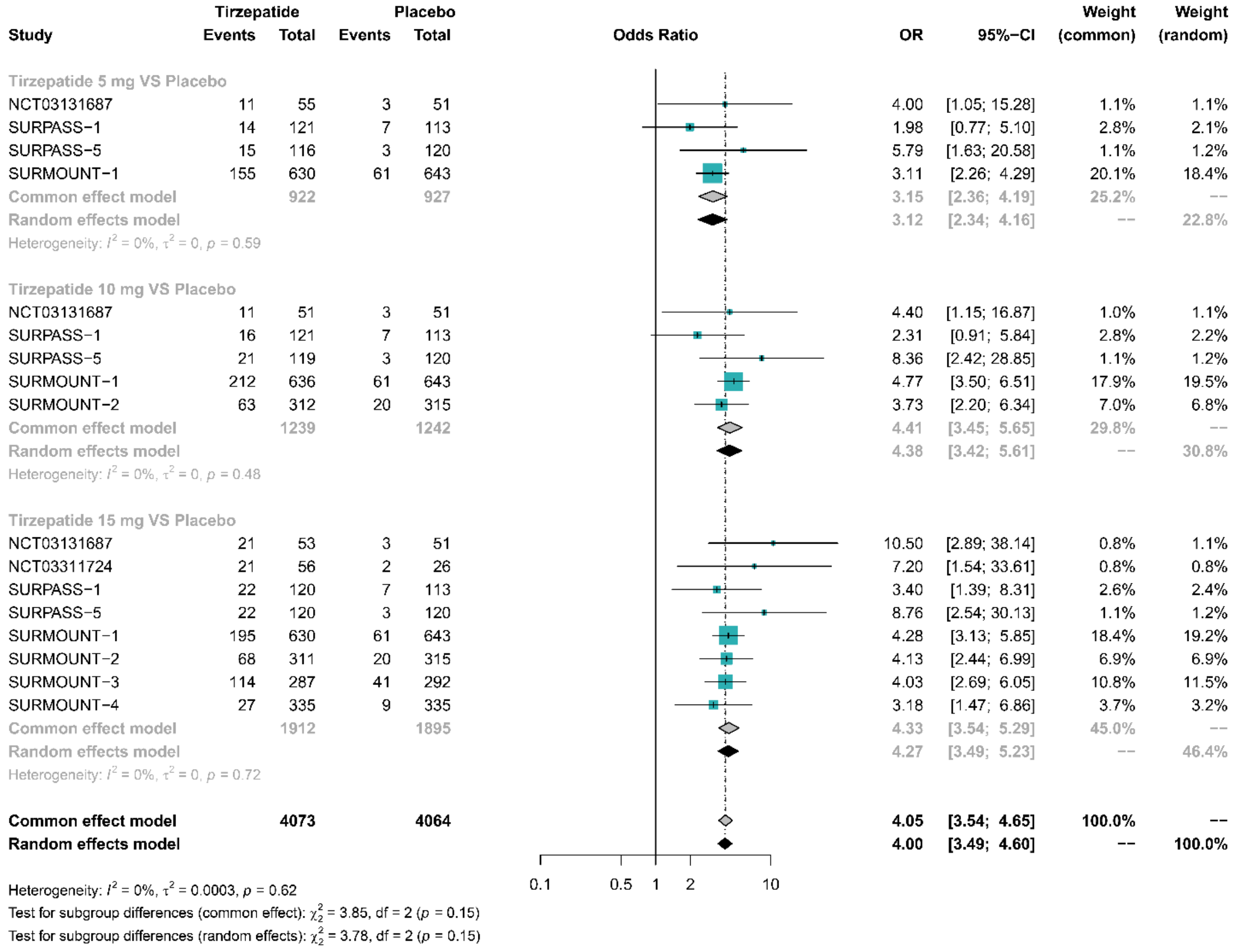

Study
Tirzepatide Events Total
Placebo Events Total
Odds Ratio
OR
95%−CI
Weight (common)
Weight (random)
Tirzepatide 5 mg VS Placebo
NCT03131687 11 55 3 51 4.00 [1.05; 15.28] 1.1% 1.1%
SURPASS−1 14 121 7 113 1.98 [0.77; 5.10] 2.8% 2.1%
SURPASS−5 15 116 3 120 5.79 [1.63; 20.58] 1.1% 1.2%
SURMOUNT−1 155 630 61 643 3.11 [2.26; 4.29] 20.1% 18.4%
Common effect model 922 927 3.15 [2.36; 4.19] 25.2% --
Random effects model 3.12 [2.34; 4.16] -- 22.8%
Heterogeneity: I^2 = 0%, τ^2 = 0, p = 0.59
Tirzepatide 10 mg VS Placebo
NCT03131687 11 51 3 51 4.40 [1.15; 16.87] 1.0% 1.1%
SURPASS−1 16 121 7 113 2.31 [0.91; 5.84] 2.8% 2.2%
SURPASS−5 21 119 3 120 8.36 [2.42; 28.85] 1.1% 1.2%
SURMOUNT−1 212 636 61 643 4.77 [3.50; 6.51] 17.9% 19.5%
SURMOUNT−2 63 312 20 315 3.73 [2.20; 6.34] 7.0% 6.8%
Common effect model 1239 1242 4.41 [3.45; 5.65] 29.8% --
Random effects model 4.38 [3.42; 5.61] -- 30.8%
Heterogeneity: I^2 = 0%, τ^2 = 0, p = 0.48
Tirzepatide 15 mg VS Placebo
NCT03131687 21 53 3 51 10.50 [2.89; 38.14] 0.8% 1.1%
NCT03311724 21 56 2 26 7.20 [1.54; 33.61] 0.8% 0.8%
SURPASS−1 22 120 7 113 3.40 [1.39; 8.31] 2.6% 2.4%
SURPASS−5 22 120 3 120 8.76 [2.54; 30.13] 1.1% 1.2%
SURMOUNT−1 195 630 61 643 4.28 [3.13; 5.85] 18.4% 19.2%
SURMOUNT−2 68 311 20 315 4.13 [2.44; 6.99] 6.9% 6.9%
SURMOUNT−3 114 287 41 292 4.03 [2.69; 6.05] 10.8% 11.5%
SURMOUNT−4 27 335 9 335 3.18 [1.47; 6.86] 3.7% 3.2%
Common effect model 1912 1895 4.33 [3.54; 5.29] 45.0% --
Random effects model 4.27 [3.49; 5.23] -- 46.4%
Heterogeneity: I^2 = 0%, τ^2 = 0, p = 0.72
Common effect model 4073 4064 4.05 [3.54; 4.65] 100.0% --
Random effects model 4.00 [3.49; 4.60] -- 100.0%
0.1 0.5 1 2 10
Heterogeneity: I^2 = 0%, τ^2 = 0.0003, p = 0.62
Test for subgroup differences (common effect): χ^2_2 = 3.85, df = 2 (p = 0.15)
Test for subgroup differences (random effects): χ^2_2 = 3.78, df = 2 (p = 0.15)

B.

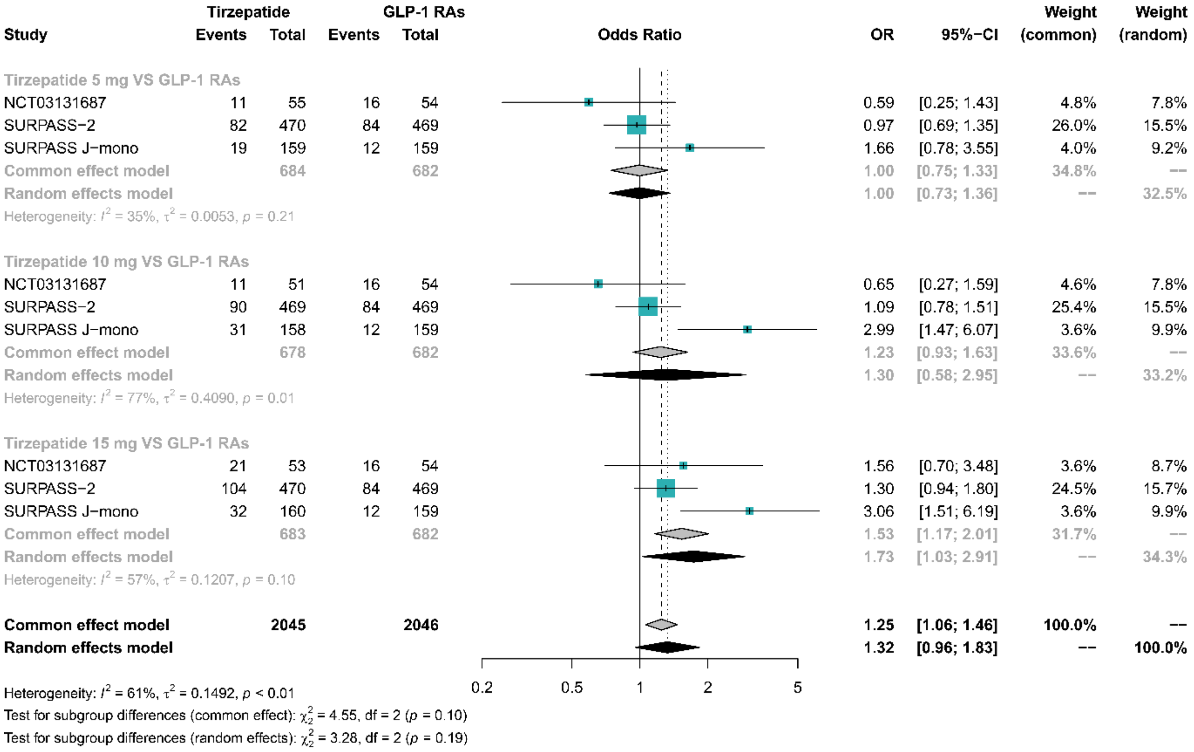

C.

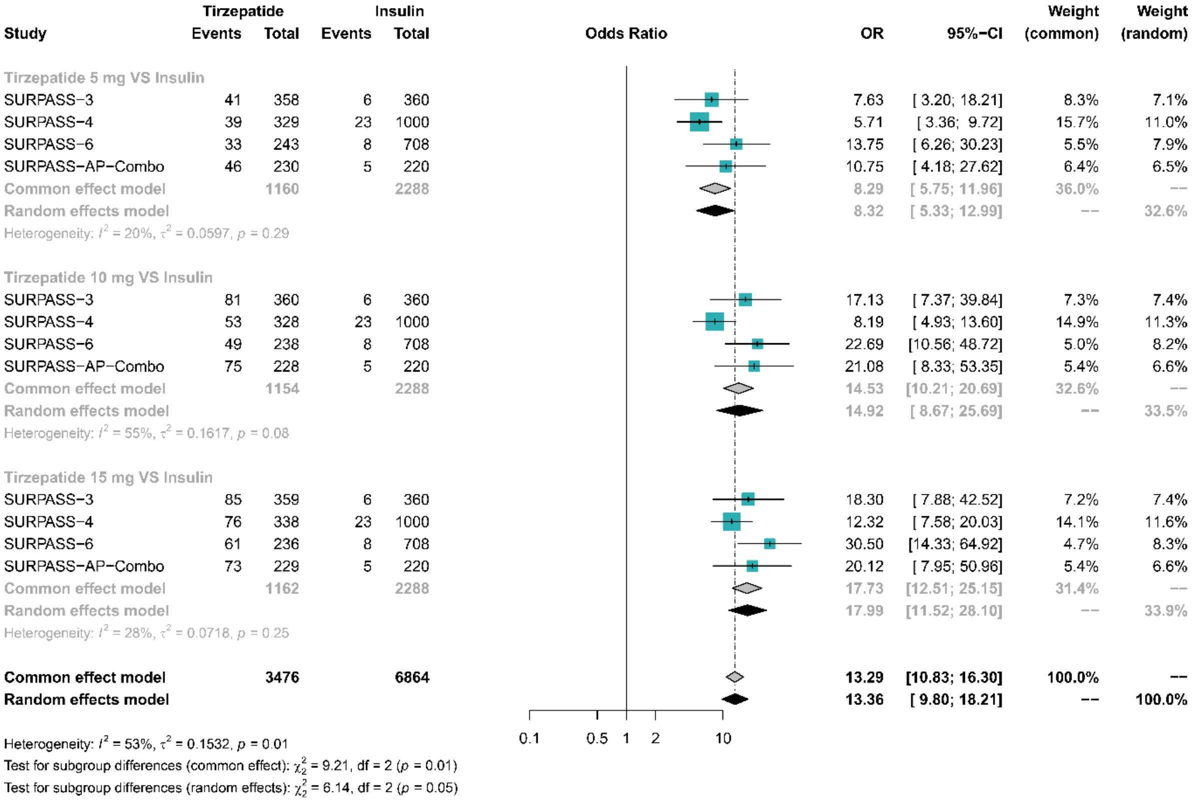

Supplementary Figure S13. Forest plot which demonstrates the proportion of patients with vomiting in different dose of tirzepatide when compared with placebo (A), glucagon like peptide-1 receptor agonist (GLP-1 RAs) (B), and insulin (C).

A.

Study	Tirzepatide Events	Tirzepatide Total	Placebo Events	Placebo Total	OR	95%-CI	Weight (common)	Weight (random)
Tirzepatide 5 mg VS Placebo								
NCT03131687	4	55	1	51	3.92	[0.42; 36.32]	1.3%	1.2%
SURPASS−1	4	121	2	113	1.90	[0.34; 10.57]	2.8%	2.1%
SURPASS−5	8	116	3	120	2.89	[0.75; 11.17]	3.8%	3.3%
SURMOUNT−1	52	630	11	643	5.17	[2.67; 10.00]	14.0%	14.0%
Common effect model		922		927	4.28	[2.49; 7.33]	21.9%	--
Random effects model					4.19	[2.43; 7.21]	--	20.6%
Heterogeneity: I^2 = 0%, τ^2 = 0, p = 0.68								
Tirzepatide 10 mg VS Placebo								
NCT03131687	8	51	1	51	9.30	[1.12; 77.38]	1.2%	1.4%
SURPASS−1	3	121	2	113	1.41	[0.23; 8.60]	2.8%	1.9%
SURPASS−5	9	119	3	120	3.19	[0.84; 12.09]	3.9%	3.4%
SURMOUNT−1	68	636	11	643	6.88	[3.60; 13.14]	13.6%	14.5%
SURMOUNT−2	34	312	10	315	3.73	[1.81; 7.69]	12.4%	11.6%
Common effect model		1239		1242	4.94	[3.23; 7.55]	33.9%	--
Random effects model					4.61	[2.83; 7.49]	--	32.8%
Heterogeneity: I^2 = 4%, τ^2 = 0.0410, p = 0.39								
Tirzepatide 15 mg VS Placebo								
NCT03131687	14	53	1	51	17.95	[2.26; 142.46]	1.0%	1.4%
NCT03311724	10	56	1	26	5.43	[0.66; 44.94]	1.6%	1.4%
SURPASS−1	7	120	2	113	3.44	[0.70; 16.91]	2.7%	2.4%
SURPASS−5	15	120	3	120	5.57	[1.57; 19.79]	3.7%	3.8%
SURMOUNT−1	77	630	11	643	8.00	[4.21; 15.20]	13.3%	14.8%
SURMOUNT−2	41	311	10	315	4.63	[2.28; 9.42]	12.0%	12.1%
SURMOUNT−3	52	287	4	292	15.93	[5.68; 44.69]	4.5%	5.7%
SURMOUNT−4	19	335	4	335	4.98	[1.67; 14.79]	5.3%	5.1%
Common effect model		1912		1895	7.20	[5.04; 10.28]	44.2%	--
Random effects model					6.76	[4.71; 9.70]	--	46.6%
Heterogeneity: I^2 = 0%, τ^2 = 0, p = 0.54								
**Common effect model**		**4073**		**4064**	**5.79**	**[4.54; 7.38]**	**100.0%**	**--**
**Random effects model**					**5.45**	**[4.26; 6.97]**	**--**	**100.0%**

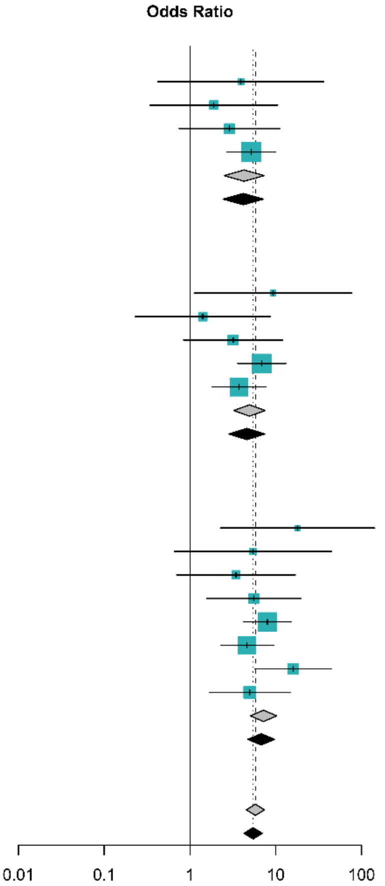

Heterogeneity: I^2 = 0%, τ^2 = 0, p = 0.57
Test for subgroup differences (common effect): χ^2_2 = 3.18, df = 2 (p = 0.20)
Test for subgroup differences (random effects): χ^2_2 = 2.73, df = 2 (p = 0.26)

B.

Study	Tirzepatide Events	Tirzepatide Total	GLP-RAs Events	GLP-RAs Total	OR	95%-CI	Weight (common)	Weight (random)
**Tirzepatide 5 mg VS GLP-1 RAs**								
NCT03131687	4	55	5	54	0.77	[0.19; 3.03]	3.7%	9.0%
SURPASS-2	27	470	39	469	0.67	[0.40; 1.12]	29.3%	15.0%
SURPASS J-mono	13	159	2	159	6.99	[1.55; 31.50]	1.5%	8.2%
Common effect model		684		682	0.95	[0.62; 1.46]	34.5%	--
Random effects model					1.37	[0.34; 5.56]	--	32.2%
Heterogeneity: $I^2 = 76\%$, $\tau^2 = 1.1805$, $p = 0.02$								
**Tirzepatide 10 mg VS GLP-1 RAs**								
NCT03131687	8	51	5	54	1.82	[0.55; 5.99]	3.3%	10.2%
SURPASS-2	40	469	39	469	1.03	[0.65; 1.63]	28.4%	15.3%
SURPASS J-mono	8	158	2	159	4.19	[0.87; 20.03]	1.5%	7.9%
Common effect model		678		682	1.25	[0.83; 1.88]	33.2%	--
Random effects model					1.51	[0.72; 3.17]	--	33.3%
Heterogeneity: $I^2 = 40\%$, $\tau^2 = 0.1924$, $p = 0.19$								
**Tirzepatide 15 mg VS GLP-1 RAs**								
NCT03131687	14	53	5	54	3.52	[1.17; 10.61]	2.9%	10.8%
SURPASS-2	46	470	39	469	1.20	[0.76; 1.87]	28.0%	15.3%
SURPASS J-mono	19	160	2	159	10.58	[2.42; 46.22]	1.4%	8.4%
Common effect model		683		682	1.81	[1.24; 2.65]	32.3%	--
Random effects model					3.03	[0.88; 10.42]	--	34.5%
Heterogeneity: $I^2 = 80\%$, $\tau^2 = 0.9133$, $p < 0.01$								
**Common effect model**		**2045**		**2046**	**1.33**	**[1.05; 1.67]**	**100.0%**	**--**
**Random effects model**					**1.85**	**[1.01; 3.39]**	**--**	**100.0%**

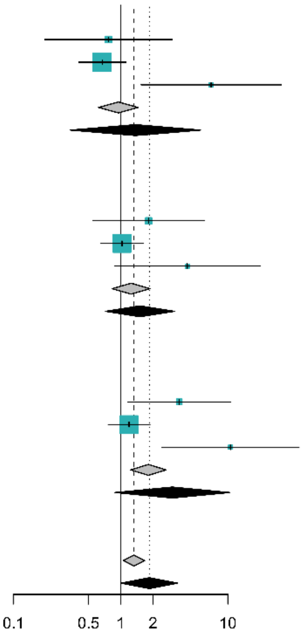

Heterogeneity: $I^2 = 69\%$, $\tau^2 = 0.5708$, $p < 0.01$
Test for subgroup differences (common effect): $\chi^2_2 = 5.02$, df = 2 ($p = 0.08$)
Test for subgroup differences (random effects): $\chi^2_2 = 1.03$, df = 2 ($p = 0.60$)

C.

Study	Tirzepatide Events	Tirzepatide Total	Insulin Events	Insulin Total	OR	95%-CI	Weight (common)	Weight (random)
**Tirzepatide 5 mg VS Insulin**								
SURPASS-3	21	358	4	360	5.55	[1.88; 16.32]	8.0%	7.2%
SURPASS-4	16	329	16	1000	3.14	[1.55; 6.36]	16.1%	11.9%
SURPASS-6	11	243	4	708	8.34	[2.63; 26.46]	4.2%	6.5%
SURPASS-AP-Combo	21	230	3	220	7.27	[2.14; 24.73]	6.0%	6.0%
Common effect model		1160		2288	5.06	[3.13; 8.17]	34.3%	--
Random effects model					4.92	[2.89; 8.36]	--	31.6%
Heterogeneity: $I^2 = 0\%$, $\tau^2 = 0.0419$, $p = 0.43$								
**Tirzepatide 10 mg VS Insulin**								
SURPASS-3	34	360	4	360	9.28	[3.26; 26.44]	7.7%	7.5%
SURPASS-4	27	328	16	1000	5.52	[2.93; 10.38]	15.5%	13.1%
SURPASS-6	21	238	4	708	17.03	[5.78; 50.16]	3.9%	7.2%
SURPASS-AP-Combo	34	228	3	220	12.68	[3.83; 41.93]	5.6%	6.2%
Common effect model		1154		2288	9.00	[5.73; 14.14]	32.8%	--
Random effects model					9.00	[5.11; 15.86]	--	34.0%
Heterogeneity: $I^2 = 22\%$, $\tau^2 = 0.1029$, $p = 0.28$								
**Tirzepatide 15 mg VS Insulin**								
SURPASS-3	36	359	4	360	9.92	[3.49; 28.17]	7.7%	7.5%
SURPASS-4	29	338	16	1000	5.77	[3.09; 10.77]	15.8%	13.3%
SURPASS-6	30	236	4	708	25.63	[8.93; 73.59]	3.7%	7.4%
SURPASS-AP-Combo	29	229	3	220	10.49	[3.15; 34.97]	5.7%	6.2%
Common effect model		1162		2288	9.81	[6.29; 15.29]	33.0%	--
Random effects model					10.23	[5.27; 19.88]	--	34.4%
Heterogeneity: $I^2 = 49\%$, $\tau^2 = 0.2210$, $p = 0.12$								
**Common effect model**		**3476**		**6864**	**7.92**	**[6.09; 10.29]**	**100.0%**	**--**
**Random effects model**					**7.85**	**[5.54; 11.12]**	**--**	**100.0%**

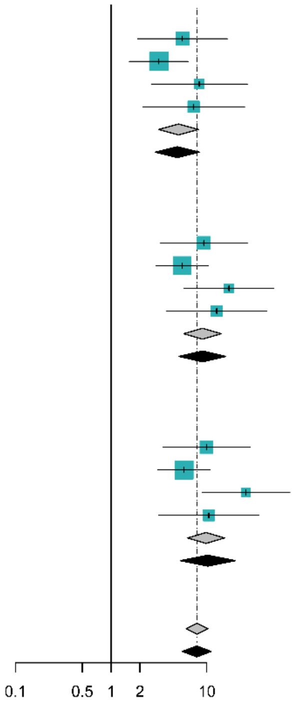

Heterogeneity: $I^2 = 34\%$, $\tau^2 = 0.1372$, $p = 0.12$
Test for subgroup differences (common effect): $\chi^2_2 = 4.55$, df = 2 ($p = 0.10$)
Test for subgroup differences (random effects): $\chi^2_2 = 3.65$, df = 2 ($p = 0.16$)

Supplementary Figure S14. Forest plot which demonstrates the proportion of patients with diarrhea in different dose of tirzepatide when compared with placebo (A), glucagon like peptide-1 receptor agonist (GLP-1 RAs) (B), and insulin (C).

A.

Study	Tirzepatide Events	Tirzepatide Total	Placebo Events	Placebo Total	OR	95%-CI	Weight (common)	Weight (random)
Tirzepatide 5 mg VS Placebo								
NCT03131687	13	55	2	51	7.58	[1.62; 35.54]	0.6%	1.3%
SURPASS−1	14	121	9	113	1.51	[0.63; 3.64]	3.3%	3.7%
SURPASS−5	14	116	12	120	1.24	[0.55; 2.80]	4.2%	4.2%
SURMOUNT−1	118	630	47	643	2.92	[2.04; 4.18]	15.2%	12.1%
Common effect model		922		927	2.55	[1.89; 3.43]	23.3%	--
Random effects model					2.25	[1.28; 3.97]	--	21.2%
Heterogeneity: $I^2 = 56\%$, $\tau^2 = 0.1670$, $p = 0.08$								
Tirzepatide 10 mg VS Placebo								
NCT03131687	12	51	2	51	7.54	[1.59; 35.69]	0.6%	1.3%
SURPASS−1	17	121	9	113	1.89	[0.81; 4.43]	3.2%	3.9%
SURPASS−5	15	119	12	120	1.30	[0.58; 2.90]	4.2%	4.3%
SURMOUNT−1	135	636	47	643	3.42	[2.40; 4.86]	14.8%	12.2%
SURMOUNT−2	62	312	28	315	2.54	[1.58; 4.10]	9.0%	8.9%
Common effect model		1239		1242	2.82	[2.19; 3.61]	31.9%	--
Random effects model					2.59	[1.79; 3.76]	--	30.6%
Heterogeneity: $I^2 = 45\%$, $\tau^2 = 0.0661$, $p = 0.12$								
Tirzepatide 15 mg VS Placebo								
NCT03131687	17	53	2	51	11.57	[2.51; 53.27]	0.6%	1.4%
NCT03311724	19	56	2	26	6.16	[1.31; 28.89]	0.7%	1.3%
SURPASS−1	14	120	9	113	1.53	[0.63; 3.68]	3.3%	3.7%
SURPASS−5	25	120	12	120	2.37	[1.13; 4.97]	3.8%	4.8%
SURMOUNT−1	145	630	47	643	3.79	[2.67; 5.38]	14.4%	12.3%
SURMOUNT−2	67	311	28	315	2.81	[1.75; 4.52]	8.8%	9.0%
SURMOUNT−3	89	287	27	292	4.41	[2.76; 7.05]	7.4%	9.1%
SURMOUNT−4	36	335	16	335	2.40	[1.30; 4.42]	5.7%	6.5%
Common effect model		1912		1895	3.37	[2.75; 4.13]	44.8%	--
Random effects model					3.26	[2.57; 4.14]	--	48.2%
Heterogeneity: $I^2 = 33\%$, $\tau^2 = 0.0200$, $p = 0.16$								
**Common effect model**		**4073**		**4064**	**3.00**	**[2.61; 3.45]**	**100.0%**	**--**
**Random effects model**					**2.83**	**[2.35; 3.41]**	**--**	**100.0%**

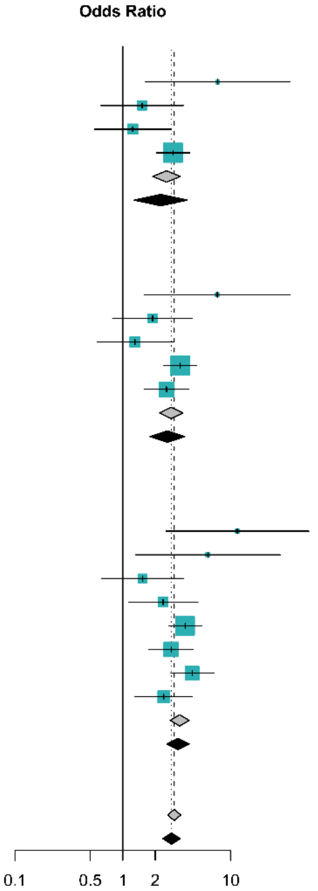

Heterogeneity: $I^2 = 41\%$, $\tau^2 = 0.0405$, $p = 0.04$
Test for subgroup differences (common effect): $\chi^2_2 = 2.68$, df = 2 ($p = 0.26$)
Test for subgroup differences (random effects): $\chi^2_2 = 2.00$, df = 2 ($p = 0.37$)

B.

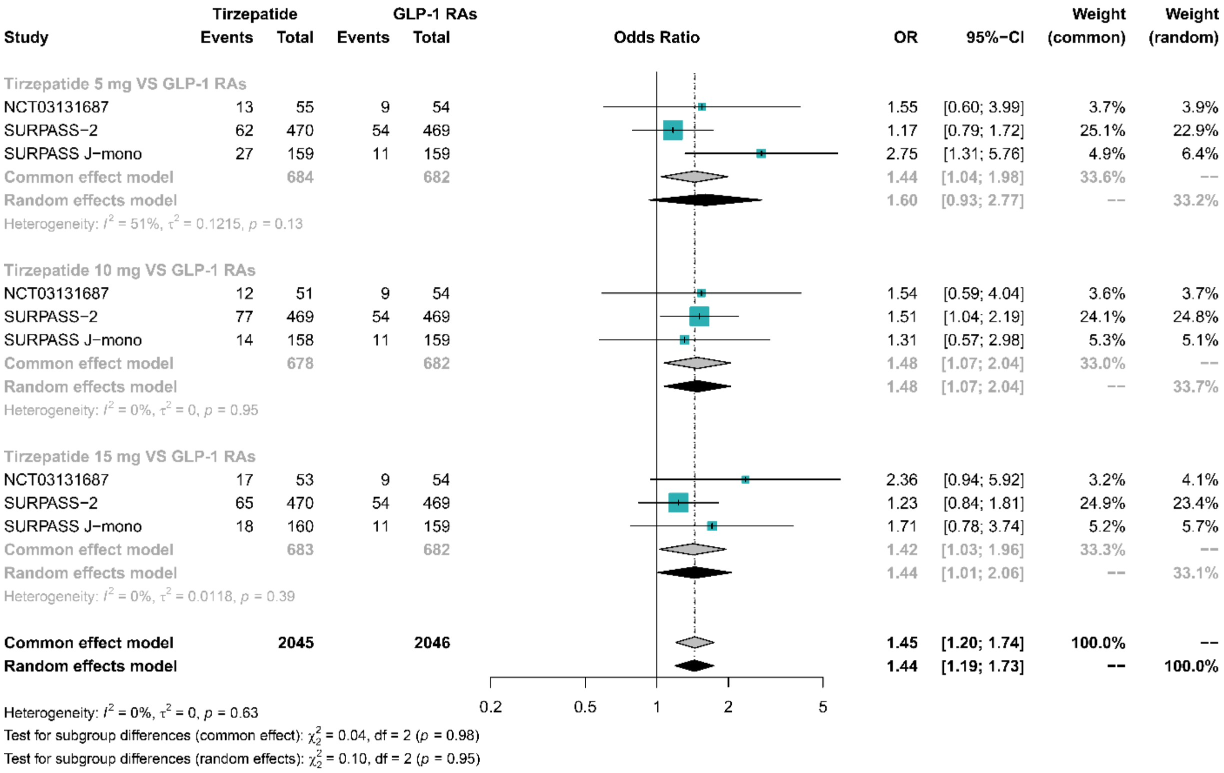

C.

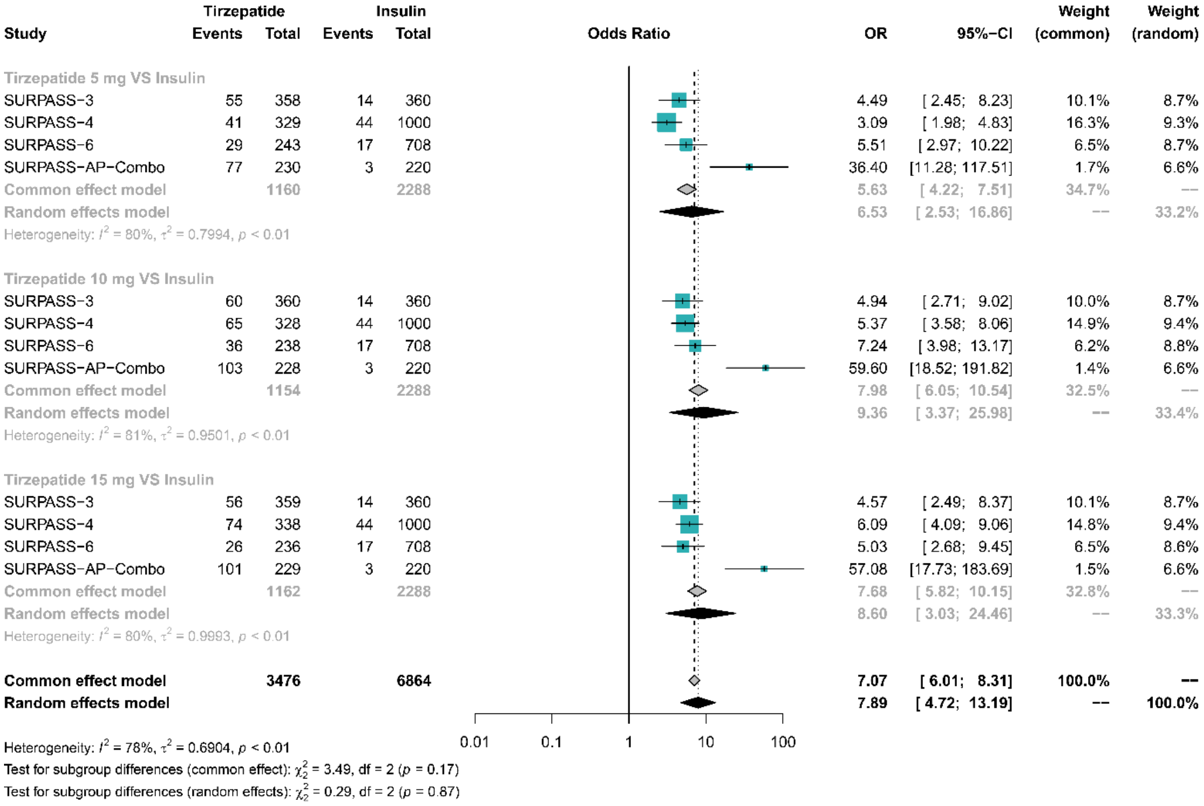

Supplementary Figure S15. Forest plot which demonstrates the proportion of patients with decreased appetite in different dose of tirzepatide when compared with placebo (A), glucagon like peptide-1 receptor agonist (GLP-1 RAs) (B), and insulin (C).

A.

Study	Tirzepatide Events	Tirzepatide Total	Placebo Events	Placebo Total	OR	95%-CI	Weight (common)	Weight (random)
**Tirzepatide 5 mg VS Placebo**								
NCT03131687	11	55	1	51	12.50	[1.55; 100.74]	0.9%	1.2%
SURPASS−1	5	121	1	113	4.83	[0.56; 41.97]	1.1%	1.1%
SURPASS−5	8	116	2	120	4.37	[0.91; 21.03]	2.0%	2.1%
SURMOUNT−1	59	630	21	643	3.06	[1.84; 5.10]	20.8%	19.9%
Common effect model		922		927	3.59	[2.27; 5.68]	24.8%	--
Random effects model					3.45	[2.18; 5.48]	--	24.3%
Heterogeneity: I^2 = 0%, τ^2 = 0, p = 0.60								
**Tirzepatide 10 mg VS Placebo**								
NCT03131687	13	51	1	51	17.11	[2.14; 136.54]	0.8%	1.2%
SURPASS−1	8	121	1	113	7.93	[0.98; 64.44]	1.1%	1.2%
SURPASS−5	15	119	2	120	8.51	[1.90; 38.09]	1.9%	2.3%
SURMOUNT−1	73	636	21	643	3.84	[2.33; 6.32]	20.4%	20.9%
SURMOUNT−2	30	312	7	315	4.68	[2.02; 10.83]	6.9%	7.4%
Common effect model		1239		1242	4.81	[3.24; 7.12]	31.1%	--
Random effects model					4.60	[3.09; 6.84]	--	32.9%
Heterogeneity: I^2 = 0%, τ^2 = 0, p = 0.57								
**Tirzepatide 15 mg VS Placebo**								
NCT03131687	10	53	1	51	11.63	[1.43; 94.54]	0.9%	1.2%
NCT03311724	14	56	0	26	18.08	[1.03; 315.94]	0.6%	0.6%
SURPASS−1	10	120	1	113	10.18	[1.28; 80.88]	1.0%	1.2%
SURPASS−5	17	120	2	120	9.74	[2.20; 43.16]	1.9%	2.3%
SURMOUNT−1	54	630	21	643	2.78	[1.66; 4.65]	20.9%	19.4%
SURMOUNT−2	31	311	7	315	4.87	[2.11; 11.24]	6.9%	7.4%
SURMOUNT−3	27	287	12	292	2.42	[1.20; 4.88]	11.9%	10.6%
Common effect model		1577		1560	3.86	[2.75; 5.42]	44.1%	--
Random effects model					4.00	[2.49; 6.41]	--	42.8%
Heterogeneity: I^2 = 23%, τ^2 = 0.1022, p = 0.25								
**Common effect model**		**3738**		**3729**	**4.09**	**[3.27; 5.11]**	**100.0%**	**--**
**Random effects model**					**3.83**	**[3.05; 4.80]**	**--**	**100.0%**

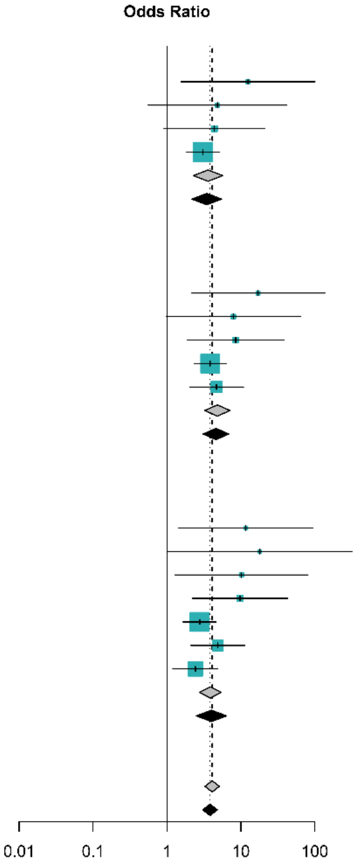

Heterogeneity: I^2 = 0%, τ^2 = 0, p = 0.54
Test for subgroup differences (common effect): χ^2_2 = 1.07, df = 2 (p = 0.59)
Test for subgroup differences (random effects): χ^2_2 = 0.86, df = 2 (p = 0.65)

B.

Study	Tirzepatide Events	Tirzepatide Total	GLP-1 RAs Events	GLP-1 RAs Total	OR	95%-CI	Weight (common)	Weight (random)
**Tirzepatide 5 mg VS GLP-1 RAs**								
NCT03131687	11	55	3	54	4.25	[1.11; 16.21]	2.6%	6.5%
SURPASS−2	35	470	25	469	1.43	[0.84; 2.43]	24.7%	15.8%
SURPASS J−mono	22	159	7	159	3.49	[1.44; 8.42]	6.4%	10.8%
Common effect model		684		682	2.04	[1.34; 3.11]	33.7%	−−
Random effects model					2.38	[1.15; 4.94]	−−	33.1%
Heterogeneity: $I^2 = 53\%$, $\tau^2 = 0.2205$, $p = 0.12$								
**Tirzepatide 10 mg VS GLP-1 RAs**								
NCT03131687	13	51	3	54	5.82	[1.55; 21.85]	2.3%	6.7%
SURPASS−2	34	469	25	469	1.39	[0.81; 2.37]	24.7%	15.7%
SURPASS J−mono	21	158	7	159	3.33	[1.37; 8.07]	6.5%	10.7%
Common effect model		678		682	2.07	[1.36; 3.15]	33.5%	−−
Random effects model					2.57	[1.12; 5.89]	−−	33.1%
Heterogeneity: $I^2 = 64\%$, $\tau^2 = 0.3327$, $p = 0.06$								
**Tirzepatide 15 mg VS GLP-1 RAs**								
NCT03131687	10	53	3	54	3.95	[1.02; 15.29]	2.6%	6.5%
SURPASS−2	42	470	25	469	1.74	[1.04; 2.91]	24.3%	16.1%
SURPASS J−mono	35	160	7	159	6.08	[2.61; 14.16]	5.9%	11.2%
Common effect model		683		682	2.69	[1.79; 4.04]	32.7%	−−
Random effects model					3.21	[1.37; 7.48]	−−	33.8%
Heterogeneity: $I^2 = 70\%$, $\tau^2 = 0.3610$, $p = 0.04$								
**Common effect model**		**2045**		**2046**	**2.26**	**[1.78; 2.88]**	**100.0%**	**−−**
**Random effects model**					**2.62**	**[1.74; 3.96]**	**−−**	**100.0%**

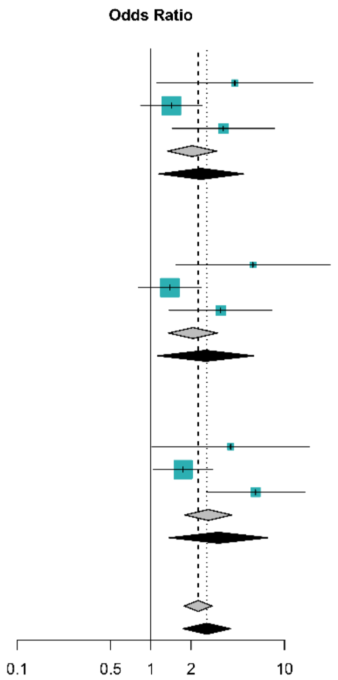

Heterogeneity: $I^2 = 54\%$, $\tau^2 = 0.2058$, $p = 0.03$
Test for subgroup differences (common effect): $\chi^2_2 = 1.11$, df = 2 ($p = 0.57$)
Test for subgroup differences (random effects): $\chi^2_2 = 0.28$, df = 2 ($p = 0.87$)

C.

Study	Tirzepatide Events	Tirzepatide Total	Insulin Events	Insulin Total	OR	95%-CI	Weight (common)	Weight (random)
**Tirzepatide 5 mg VS Insulin**								
SURPASS−3	22	358	2	360	11.72	[2.74; 50.22]	13.0%	8.0%
SURPASS−4	29	329	5	1000	19.24	[7.38; 50.13]	15.7%	18.4%
SURPASS−6	20	243	1	708	63.41	[8.46; 475.13]	3.3%	4.2%
SURPASS−AP−Combo	74	230	0	220	209.93	[12.91; 3413.24]	2.4%	2.2%
Common effect model		1160		2288	33.93	[16.38; 70.30]	34.4%	−−
Random effects model					23.25	[11.34; 47.70]	−−	32.7%
Heterogeneity: $I^2 = 31\%$, $\tau^2 = < 0.0001$, $p = 0.23$								
**Tirzepatide 10 mg VS Insulin**								
SURPASS−3	37	360	2	360	20.50	[4.90; 85.75]	12.5%	8.3%
SURPASS−4	36	328	5	1000	24.53	[9.54; 63.09]	15.3%	18.9%
SURPASS−6	28	238	1	708	94.27	[12.75; 696.96]	3.1%	4.2%
SURPASS−AP−Combo	109	228	0	220	404.10	[24.89; 6559.62]	1.8%	2.2%
Common effect model		1154		2288	51.01	[25.06; 103.83]	32.7%	−−
Random effects model					38.06	[15.43; 93.90]	−−	33.6%
Heterogeneity: $I^2 = 40\%$, $\tau^2 = 0.2293$, $p = 0.17$								
**Tirzepatide 15 mg VS Insulin**								
SURPASS−3	43	359	2	360	24.36	[5.85; 101.36]	12.2%	8.3%
SURPASS−4	35	338	5	1000	22.99	[8.93; 59.19]	15.8%	18.9%
SURPASS−6	40	236	1	708	144.29	[19.71; 1056.17]	2.9%	4.3%
SURPASS−AP−Combo	102	229	0	220	354.53	[21.84; 5755.41]	2.0%	2.2%
Common effect model		1162		2288	54.02	[26.58; 109.82]	32.9%	−−
Random effects model					46.87	[15.57; 141.07]	−−	33.6%
Heterogeneity: $I^2 = 46\%$, $\tau^2 = 0.5704$, $p = 0.13$								
**Common effect model**		**3476**		**6864**	**46.12**	**[30.53; 69.67]**	**100.0%**	**−−**
**Random effects model**					**30.15**	**[19.99; 45.49]**	**−−**	**100.0%**

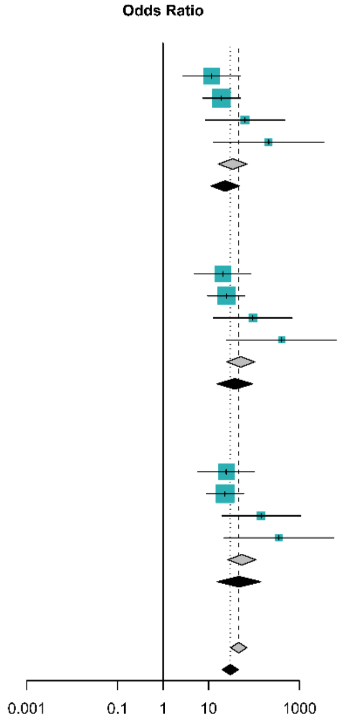

Heterogeneity: $I^2 = 30\%$, $\tau^2 < 0.0001$, $p = 0.15$
Test for subgroup differences (common effect): $\chi^2_2 = 0.95$, df = 2 ($p = 0.62$)
Test for subgroup differences (random effects): $\chi^2_2 = 1.35$, df = 2 ($p = 0.51$)
